# Supplementary material for: The NUPHAC-EU Framework for Nurses’ Role in Interprofessional Pharmaceutical Care: Cross-Sectional Evaluation in Europe
Source: Int J Environ Res Public Health. 2021 Jul 25;18(15):7862. doi: 10.3390/ijerph18157862 (PMC8345454; doi:10.3390/ijerph18157862)
Supplement: Supplementary file 1 [file ijerph-18-07862-s001.zip › Supplementary File_S1_STROBE.pdf]

STROBE Statement—checklist of items that should be included in reports of observational studies

	Item No.	Recommendation	**Relevant text from manuscript** 'The NUPHAC-EU framework about NUrses' role in interprofessional PHArmaceutical care: Cross-sectional evaluation in Europe'
**Title and abstract**	1	(*a*) Indicate the study's design with a commonly used term in the title or the abstract	Cross-sectional design
		(*b*) Provide in the abstract an informative and balanced summary of what was done and what was found	See full abstract
Background/rationale	2	Explain the scientific background and rationale for the investigation being reported	See introduction
Objectives	3	State specific objectives, including any prespecified hypotheses	To create and evaluate a framework describing potential tasks for nurses in PC and to evaluate to what extent physicians, pharmacists and nurses from 14 European countries consider PC-related tasks beyond preparation and administration of medicines as nurses' responsibility in an ideal healthcare situation with best quality of interprofessional care and patient outcomes .
Study design	4	Present key elements of study design early in the paper	Quantitative, cross-sectional study
Setting	5	Describe the setting, locations, and relevant dates, including periods of recruitment, exposure, follow-up, and data collection	14 European countries.<br>Clinical practice, education, research and policy making.<br>December 2019 – August 2020
Participants	6	~~(*a*) *Cohort study*—Give the eligibility criteria, and the sources and methods of selection of participants. Describe methods of follow-up~~	Nurses, physicians and pharmacists employed in clinical practice (community care, residential care, hospital care and mental healthcare), education, research, and policy

		Case-control study—Give the eligibility criteria, and the sources and methods of case ascertainment and control selection. Give the rationale for the choice of cases and controls<br>*Cross-sectional study*—Give the eligibility criteria, and the sources and methods of selection of participants	making. Professionals in training and students were excluded.<br>Weblink to questionnaire was emailed to key stakeholders, professional associations, healthcare facilities and professional networks of the researchers in all countries. Nursing faculties as well as interprofesional colleagues initiated data collection. The weblink was placed on university websites, webpages of professional associations and on social media.
		(*b*) *Cohort study*—For matched studies, give matching criteria and number of exposed and unexposed<br>*Case-control study*—For matched studies, give matching criteria and the number of controls per case	
Variables	7	Clearly define all outcomes, exposures, predictors, potential confounders, and effect modifiers. Give diagnostic criteria, if applicable	The main outcome variable was the level of responsibility in PC tasks (not allowed, under supervision, with shared responsibility or fully autonomous) that would be assigned to nurses in een ideal situation with best quality of interprofessional care and patient outcomes
Data sources/ measurement	8*	For each variable of interest, give sources of data and details of methods of assessment (measurement). Describe comparability of assessment methods if there is more than one group	Online survey in nurses, physicians and pharmacist. All three groups were questioned in the same way. Online survey in 14 countries: the questionnaire was translated into all languages of the participating countries
Bias	9	Describe any efforts to address potential sources of bias	Selection bias: inclusion of healthcare workers in 14 countries (own language), different settings.<br>Informing a wide range of organisations about the research.<br>Present a shorter survey by random selection of 4 of the 7 responsibilities to avoid drop-out of respondents.
Study size	10	Explain how the study size was arrived at	14 countries: the countries were selected in an earlier phase of the overarching DeMoPhaC project of which this study is part.

			Informing a wide range of organisations about the research. Weblink to questionnaire was emailed to key stakeholders, professional associations, healthcare facilities and professional networks of the researchers in all countries. Nursing faculties as well as interprofessional colleagues initiated data collection. The weblink was placed on university websites, webpages of professional associations and on social media.<br>Data collection period of 9 months to reach the current sample size.
Quantitative variables	11	Explain how quantitative variables were handled in the analyses. If applicable, describe which groupings were chosen and why	Discontinuous data were described using frequency distributions; continuous data were described using a mean value, a minimum and a maximum.<br>Groupings: 3 professional groups, 14 countries (or less if a country had less than 28 responses. This number was calculated, using a power analysis)
Statistical methods	12	(*a*) Describe all statistical methods, including those used to control for confounding	To evaluate the statistical significance of the differences between the three professional groups or between the 14 countries, $\chi 2$ test for nominal variables, and Kruskal-Wallis test for ordinal variables were used.
		(*b*) Describe any methods used to examine subgroups and interactions	idem
		(*c*) Explain how missing data were addressed	Respondents who ended the survey during or immediately after the first part of the questionnaire (demographics, employment, education) were excluded from the data analysis because they did not provide data relevant to the research question. All other data were reported using valid percentages + presentation of n-values
		(*d*) *Cohort study*—If applicable, explain how loss to follow-up was addressed	NA

		Case-control study—If applicable, explain how matching of cases and controls was addressed<br>*Cross-sectional study*—If applicable, describe analytical methods taking account of sampling strategy	
		(*e*) Describe any sensitivity analyses	NA
Participants	13*	(a) Report numbers of individuals at each stage of study—eg numbers potentially eligible, examined for eligibility, confirmed eligible, included in the study, completing follow-up, and analysed	Cross-sectional study – 1 stage. Numbers are reported in all sections.
		(b) Give reasons for non-participation at each stage	NA
		(c) Consider use of a flow diagram	NA
Descriptive data	14*	(a) Give characteristics of study participants (eg demographic, clinical, social) and information on exposures and potential confounders	Description of ‘research population to evaluate the NuPhaC-EU framework + table 2. Population characteristics
		(b) Indicate number of participants with missing data for each variable of interest	NA
		~~(c) *Cohort study*—Summarise follow-up time (eg, average and total amount)~~	
Outcome data	15*	~~*Cohort study*—Report numbers of outcome events or summary measures over time~~	
		~~*Case-control study*—Report numbers in each exposure category, or summary measures of exposure~~	
		Cross-sectional study—Report numbers of outcome events or summary measures	Yes, see results
Main results	16	(*a*) Give unadjusted estimates and, if applicable, confounder-adjusted estimates and their precision (eg, 95% confidence interval). Make clear which confounders were adjusted for and why they were included	Only descriptive results. No estimates.
		(*b*) Report category boundaries when continuous variables were categorized	NA

		(*c*) If relevant, consider translating estimates of relative risk into absolute risk for a meaningful time period	NA
Other analyses	17	Report other analyses done—eg analyses of subgroups and interactions, and sensitivity analyses	No other analyses
Key results	18	Summarise key results with reference to study objectives	First paragraph of discussion
Limitations	19	Discuss limitations of the study, taking into account sources of potential bias or imprecision. Discuss both direction and magnitude of any potential bias	This internet survey had limitations. The inclusion or exclusion of countries and respondents was determined by whether they were included in the overarching Erasmus+ project. Also, this self-selected sample with an unknown response rate might have led to a distortion of the results due to only the most motivated professionals participating. The enormous workload of healthcare workers at the time of the COVID-19 pandemic forced a huge part of the professionals to neglect activities such as completing scientific surveys. The sample also favoured more educated computer-literate professionals, because of the internet recruitment. Finally, as with all self-reports, we cannot discount acquiescence response bias. The views of 1385 professionals are important, yet, we have to assume some might have been biased by socially desirable responding.
Interpretation	20	Give a cautious overall interpretation of results considering objectives, limitations, multiplicity of analyses, results from similar studies, and other relevant evidence	See discussion
Generalisability	21	Discuss the generalisability (external validity) of the study results	-When interpreting the results of this study, it is of major importance to recognize that more than half of the participants were nurses. This might have distorted our results. Nevertheless, we are convinced of the great value of the NuPhaC-EU framework, which aimed to offer healthcare professionals a discussion tool in a wide range of interprofessional PC situations.<br>-Despite the limited number of participants at the national level in some countries, the overall sample size was satisfactory and

			provided interesting insights in the extent to what European healthcare professionals consider PC-related tasks as nurses' responsibility in an ideal healthcare situation with best quality of interprofessional care and patient outcomes.
**Other information**			
Funding	22	Give the source of funding and the role of the funders for the present study and, if applicable, for the original study on which the present article is based	This study was supported by the Erasmus+ Programme of the European Union [grant number 2018-1-BE02-KA203-046861] and MDMJ accountants, Belgium. The funders had no role in study design, data collection and analysis, decision to publish, or preparation of the manuscript.

*Give information separately for cases and controls in case-control studies and, if applicable, for exposed and unexposed groups in cohort and cross-sectional studies.

Note: An Explanation and Elaboration article discusses each checklist item and gives methodological background and published examples of transparent reporting. The STROBE checklist is best used in conjunction with this article (freely available on the Web sites of PLoS Medicine at http://www.plosmedicine.org/, Annals of Internal Medicine at http://www.annals.org/, and Epidemiology at http://www.epidem.com/). Information on the STROBE Initiative is available at www.strobe-statement.org.
