# Supplementary material for: The NUPHAC-EU Framework for Nurses’ Role in Interprofessional Pharmaceutical Care: Cross-Sectional Evaluation in Europe
Source: Int J Environ Res Public Health. 2021 Jul 25;18(15):7862. doi: 10.3390/ijerph18157862 (PMC8345454; doi:10.3390/ijerph18157862)
Supplement: Supplementary file 1 [file ijerph-18-07862-s001.zip › Supplementary File_S2_ survey with code book.pdf]

Nurses’ roles in interprofessional pharmaceutical care - survey

SURVEY WITH CODES AND VALUES USED IN SPSS DATABASE

ResponseId
Progress (completeness rate of the survey)

Q1/ In which country are you currently working?

- Belgium 1
- Czech Republic 2
- Germany 3
- Greece4
- Hungary 5
- Italy6
- The Netherlands7
- Norway8
- Portugal9
- Republic of North Macedonia 10
- Slovakia11
- Slovenia12
- Spain13
- UK (England) 14
- UK (Wales) 15
- Other16:Q1_16_TEXT

Qextra/ question only for Belgium (province)
Q32/question only for Czech Republic: Uveďte kraj, ve kterém pracujete: Hlavní město Praha(1), Středočeský kraj(4), Pardubický kraj(5), Královehradecký kraj(6), Liberecký kraj(7), Ústecký kraj(8), Karlovarský kraj (9), Plzeňský kraj (10), Jihočeský kraj (11), Kraj Vysočina (12), Jihomoravský kraj (13), Zlínský kraj (14), Olomoucký kraj (15), Moravskoslezský kraj (16), Preferuji neuvádět (17)
Q33/ question only for Czech Republic: Uveďte typ poskytovatele péče: Fakultní nemocnice(1), Soukromé zařízení(4), Státní (krajské, oblastní) zařízení (5), Preferuji neuvádět (6), Řízený krajem (8)

Q2/What is your gender?

- Male1
- Female2
- Other3
- Prefer not to say4

Q3/What is your age? …. Years Q3_1

Q4/ How many years of work experience do you have in healthcare? …. Years Q4_1

Q5/What is your profession? Q5

- Physician1
- Pharmacist2
- Nurse3
- Other (by clicking this option the survey will end for you) 4

Q35/ question only for Germany: Welches höchste pflegerische Kompetenzniveau haben Sie? Niveau 2oder3(1), niveau 4(4), niveau 5(5), niveau 6 (6), niveau 7 (9), niveau 8 (10)

Q6/Question only for nurses: What is your highest level of nursing education?

- Level 4 (option not shown in all countries) 1
- Level 5 (not in the Netherlands) 2
- Level 6 3
- Level 74
- Level 8 5
- Other 6: …. Q_6_TEXT

Q34/ only for UK: Have you successfully completed an M-level prescribing module to qualify you to prescribe? Yes=2, no=1

Q7/In which healthcare setting do you have experience? (please tick all that apply)

- Q7_1Community or primary care yes=1,no=0
- Q7_2Residential care yes=1,no=0
- Q7_3Hospital care yes=1,no=0
- Q7_5Mental healthcare yes=1,no=0
- Q7_4No experience in clinical practice yes=1,no=0
- Only UK: Q7_6Other=1,no=0:… Q7_6_TEXT

Q8/Which of these best describes your employment at the moment? (please tick all that apply)

- Q7_1Clinical practice yes=1,no=0
- Q8_2Research yes=1,no=0
- Q8_3Education yes=1,no=0
- Q8_4Policy making yes=1,no=0

Q9/**Can you make a distinction between nurse responsibilities based on their educational level?**

- 1Yes, I can (-> only questions Q10b Q11b Q12 Q13b Q14b Q17b Q18b Q19b will be shown)
- 2No, I can't (-> only questions Q10a Q11a Q12a Q13a Q14a Q17a Q18a Q19b will be shown)

Q10a/ RESPONSIBILITY 1: Management of therapeutic and adverse effects of medicines.

	**Task NOT RELEVANT** within 'management of therapeutic and adverse effects of medicines'. (skip following columns, when this options is ticked)	**Task PROHIBTED** for nurses within 'management of therapeutic and adverse effects of medicines'. (skip following columns, when this options is ticked)	Task **PERMITTED** for **all levels** of nurses within 'management of therapeutic and adverse effects of medicines'. Specify the level of responsibility. (skip following columns, when this options is ticked)	Specify the level of responsibility for **level 4** nurses performing this task within 'management of therapeutic and adverse effects of medicines'. Variable only applicable in Germany and the Netherlands	Specify the level of responsibility for **level 5** nurses performing this task within 'management of therapeutic and adverse effects of medicines'.	Specify the level of responsibility for **level 6** nurses performing this task within 'management of therapeutic and adverse effects of medicines'.	Specify the level of responsibility for **level 7** nurses performing this task within 'management of therapeutic and adverse effects of medicines.'	Specify the level of responsibility for **level 8** nurses performing this task within 'management of therapeutic and adverse effects of medicines.'
observation, documentation, registration, reporting	**Q10a_4_1_1**<br>Ticked = 1<br>Not ticked = 0	**Q10a_2_1_1**<br>Ticked = 1<br>Not ticked = 0	**Q10a_3_1**<br>1: under supervision<br>2: shared responsibility<br>3: full autonomy	**Q36_5_1**<br>1: not allowed<br>2: under supervision<br>3: shared responsibility<br>4: full autonomy	**Q10a_5_1**<br>1: not allowed<br>2: under supervision<br>3: shared responsibility<br>4: full autonomy	**Q10a_1_1**<br>1: not allowed<br>2: under supervision<br>3: shared responsibility<br>4: full autonomy	**Q10a_6_1**<br>1: not allowed<br>2: under supervision<br>3: shared responsibility<br>4: full autonomy	**Q10a_7_1**<br>1: not allowed<br>2: under supervision<br>3: shared responsibility<br>4: full autonomy
assessing patients' competences	**Q10a_4_2_1**<br>Ticked = 1<br>Not ticked = 0	**Q10a_2_2_1**<br>Ticked = 1<br>Not ticked = 0	**Q10a_3_2**<br>1: under supervision<br>2: shared responsibility<br>3: full autonomy	**Q36_5_2**<br>1: not allowed<br>2: under supervision<br>3: shared responsibility<br>4: full autonomy	**Q10a_5_2**<br>1: not allowed<br>2: under supervision<br>3: shared responsibility<br>4: full autonomy	**Q10a_1_2**<br>1: not allowed<br>2: under supervision<br>3: shared responsibility<br>4: full autonomy	**Q10a_6_2**<br>1: not allowed<br>2: under supervision<br>3: shared responsibility<br>4: full autonomy	**Q10a_7_2**<br>1: not allowed<br>2: under supervision<br>3: shared responsibility<br>4: full autonomy
assessment and addressing patient/family needs	**Q10a_4_3_1**<br>Ticked = 1<br>Not ticked = 0	**Q10a_2_3_1**<br>Ticked = 1<br>Not ticked = 0	**Q10a_3_3**<br>1: under supervision<br>2: shared responsibility<br>3: full autonomy	**Q36_5_3**<br>1: not allowed<br>2: under supervision<br>3: shared responsibility	**Q10a_5_3**<br>1: not allowed<br>2: under supervision<br>3: shared responsibility	**Q10a_1_3**<br>1: not allowed<br>2: under supervision<br>3: shared responsibility	**Q10a_6_3**<br>1: not allowed<br>2: under supervision<br>3: shared responsibility	**Q10a_7_3**<br>1: not allowed<br>2: under supervision<br>3: shared responsibility

				4: full autonomy	4: full autonomy	4: full autonomy	4: full autonomy	4: full autonomy
recognising & preventing risks / complications / medication errors	Q10a_4_4_1<br>Ticked = 1<br>Not ticked = 0	Q10a_2_4_1<br>Ticked = 1<br>Not ticked = 0	Q10a_3_4<br>1: under supervision<br>2: shared responsibility<br>3: full autonomy	Q36_5_4<br>1: not allowed<br>2: under supervision<br>3: shared responsibility<br>4: full autonomy	Q10a_5_4<br>1: not allowed<br>2: under supervision<br>3: shared responsibility<br>4: full autonomy	Q10a_1_4<br>1: not allowed<br>2: under supervision<br>3: shared responsibility<br>4: full autonomy	Q10a_6_4<br>1: not allowed<br>2: under supervision<br>3: shared responsibility<br>4: full autonomy	Q10a_7_4<br>1: not allowed<br>2: under supervision<br>3: shared responsibility<br>4: full autonomy
identification, reporting and addressing contra-indications to administration, identification of drug related problems	Q10a_4_5_1<br>Ticked = 1<br>Not ticked = 0	Q10a_2_5_1<br>Ticked = 1<br>Not ticked = 0	Q10a_3_5<br>1: under supervision<br>2: shared responsibility<br>3: full autonomy	Q36_5_5<br>1: not allowed<br>2: under supervision<br>3: shared responsibility<br>4: full autonomy	Q10a_5_5<br>1: not allowed<br>2: under supervision<br>3: shared responsibility<br>4: full autonomy	Q10a_1_5<br>1: not allowed<br>2: under supervision<br>3: shared responsibility<br>4: full autonomy	Q10a_6_5<br>1: not allowed<br>2: under supervision<br>3: shared responsibility<br>4: full autonomy	Q10a_7_5<br>1: not allowed<br>2: under supervision<br>3: shared responsibility<br>4: full autonomy
follow-up	Q10a_4_6_1<br>Ticked = 1<br>Not ticked = 0	Q10a_2_6_1<br>Ticked = 1<br>Not ticked = 0	Q10a_3_6<br>1: under supervision<br>2: shared responsibility<br>3: full autonomy	Q36_5_6<br>1: not allowed<br>2: under supervision<br>3: shared responsibility<br>4: full autonomy	Q10a_5_6<br>1: not allowed<br>2: under supervision<br>3: shared responsibility<br>4: full autonomy	Q10a_1_6<br>1: not allowed<br>2: under supervision<br>3: shared responsibility<br>4: full autonomy	Q10a_6_6<br>1: not allowed<br>2: under supervision<br>3: shared responsibility<br>4: full autonomy	Q10a_7_6<br>1: not allowed<br>2: under supervision<br>3: shared responsibility<br>4: full autonomy
Adhering to evidence based practice as described in clinical guidelines	Q10a_4_7_1<br>Ticked = 1<br>Not ticked = 0	Q10a_2_7_1<br>Ticked = 1<br>Not ticked = 0	Q10a_3_7<br>1: under supervision<br>2: shared responsibility<br>3: full autonomy	Q36_5_7<br>1: not allowed<br>2: under supervision<br>3: shared responsibility<br>4: full autonomy	Q10a_5_7<br>1: not allowed<br>2: under supervision<br>3: shared responsibility<br>4: full autonomy	Q10a_1_7<br>1: not allowed<br>2: under supervision<br>3: shared responsibility<br>4: full autonomy	Q10a_6_7<br>1: not allowed<br>2: under supervision<br>3: shared responsibility<br>4: full autonomy	Q10a_7_7<br>1: not allowed<br>2: under supervision<br>3: shared responsibility<br>4: full autonomy
decision making	Q10a_4_8_1<br>Ticked = 1<br>Not ticked = 0	Q10a_2_8_1<br>Ticked = 1<br>Not ticked = 0	Q10a_3_8<br>1: under supervision<br>2: shared responsibility<br>3: full autonomy	Q36_5_8<br>1: not allowed<br>2: under supervision<br>3: shared responsibility<br>4: full autonomy	Q10a_5_8<br>1: not allowed<br>2: under supervision<br>3: shared responsibility<br>4: full autonomy	Q10a_1_8<br>1: not allowed<br>2: under supervision<br>3: shared responsibility<br>4: full autonomy	Q10a_6_8<br>1: not allowed<br>2: under supervision<br>3: shared responsibility<br>4: full autonomy	Q10a_7_8<br>1: not allowed<br>2: under supervision<br>3: shared responsibility<br>4: full autonomy
communication /discussion with patient/family	Q10a_4_9_1<br>Ticked = 1<br>Not ticked = 0	Q10a_2_9_1<br>Ticked = 1<br>Not ticked = 0	Q10a_3_9<br>1: under supervision<br>2: shared responsibility<br>3: full autonomy	Q36_5_9<br>1: not allowed<br>2: under supervision<br>3: shared responsibility<br>4: full autonomy	Q10a_5_9<br>1: not allowed<br>2: under supervision<br>3: shared responsibility<br>4: full autonomy	Q10a_1_9<br>1: not allowed<br>2: under supervision<br>3: shared responsibility<br>4: full autonomy	Q10a_6_9<br>1: not allowed<br>2: under supervision<br>3: shared responsibility<br>4: full autonomy	Q10a_7_9<br>1: not allowed<br>2: under supervision<br>3: shared responsibility<br>4: full autonomy
Inter / intraprofessional referrals	Q10a_4_10_1<br>Ticked = 1<br>Not ticked = 0	Q10a_2_10_1<br>Ticked = 1<br>Not ticked = 0	Q10a_3_10<br>1: under supervision<br>2: shared responsibility<br>3: full autonomy	Q36_5_10<br>1: not allowed<br>2: under supervision<br>3: shared responsibility<br>4: full autonomy	Q10a_5_10<br>1: not allowed<br>2: under supervision<br>3: shared responsibility<br>4: full autonomy	Q10a_1_10<br>1: not allowed<br>2: under supervision<br>3: shared responsibility<br>4: full autonomy	Q10a_6_10<br>1: not allowed<br>2: under supervision<br>3: shared responsibility<br>4: full autonomy	Q10a_7_10<br>1: not allowed<br>2: under supervision<br>3: shared responsibility<br>4: full autonomy
facilitation of medication management	Q10a_4_12_1<br>Ticked = 1<br>Not ticked = 0	Q10a_2_12_1<br>Ticked = 1<br>Not ticked = 0	Q10a_3_12<br>1: under supervision<br>2: shared responsibility<br>3: full autonomy	Q36_5_12<br>1: not allowed<br>2: under supervision<br>3: shared responsibility<br>4: full autonomy	Q10a_5_12<br>1: not allowed<br>2: under supervision<br>3: shared responsibility<br>4: full autonomy	Q10a_1_12<br>1: not allowed<br>2: under supervision<br>3: shared responsibility<br>4: full autonomy	Q10a_6_12<br>1: not allowed<br>2: under supervision<br>3: shared responsibility<br>4: full autonomy	Q10a_7_12<br>1: not allowed<br>2: under supervision<br>3: shared responsibility<br>4: full autonomy
self-care support and education of patients	Q10a_4_15_1<br>Ticked = 1<br>Not ticked = 0	Q10a_2_15_1<br>Ticked = 1<br>Not ticked = 0	Q10a_3_15<br>1: under supervision<br>2: shared responsibility<br>3: full autonomy	Q36_5_15<br>1: not allowed<br>2: under supervision<br>3: shared responsibility<br>4: full autonomy	Q10a_5_15<br>1: not allowed<br>2: under supervision<br>3: shared responsibility<br>4: full autonomy	Q10a_1_15<br>1: not allowed<br>2: under supervision<br>3: shared responsibility<br>4: full autonomy	Q10a_6_15<br>1: not allowed<br>2: under supervision<br>3: shared responsibility<br>4: full autonomy	Q10a_7_15<br>1: not allowed<br>2: under supervision<br>3: shared responsibility<br>4: full autonomy

advice (to patient or other healthcare professional)	Q10a_4_16_1<br>Ticked = 1<br>Not ticked = 0	Q10a_2_16_1<br>Ticked = 1<br>Not ticked = 0	Q10a_3_16<br>1: under supervision<br>2: shared responsibility<br>3: full autonomy	Q36_5_16<br>1: not allowed<br>2: under supervision<br>3: shared responsibility<br>4: full autonomy	Q10a_5_16<br>1: not allowed<br>2: under supervision<br>3: shared responsibility<br>4: full autonomy	Q10a_1_16<br>1: not allowed<br>2: under supervision<br>3: shared responsibility<br>4: full autonomy	Q10a_6_16<br>1: not allowed<br>2: under supervision<br>3: shared responsibility<br>4: full autonomy	Q10a_7_16<br>1: not allowed<br>2: under supervision<br>3: shared responsibility<br>4: full autonomy
transitional care communication, inter / intraprofessional collaboration / communication including reporting, advising, informing, alerting and discussing	Q10a_4_17_1<br>Ticked = 1<br>Not ticked = 0	Q10a_2_17_1<br>Ticked = 1<br>Not ticked = 0	Q10a_3_17<br>1: under supervision<br>2: shared responsibility<br>3: full autonomy	Q36_5_17<br>1: not allowed<br>2: under supervision<br>3: shared responsibility<br>4: full autonomy	Q10a_5_17<br>1: not allowed<br>2: under supervision<br>3: shared responsibility<br>4: full autonomy	Q10a_1_17<br>1: not allowed<br>2: under supervision<br>3: shared responsibility<br>4: full autonomy	Q10a_6_17<br>1: not allowed<br>2: under supervision<br>3: shared responsibility<br>4: full autonomy	Q10a_7_17<br>1: not allowed<br>2: under supervision<br>3: shared responsibility<br>4: full autonomy
mentoring colleagues	Q10a_4_18_1<br>Ticked = 1<br>Not ticked = 0	Q10a_2_18_1<br>Ticked = 1<br>Not ticked = 0	Q10a_3_18<br>1: under supervision<br>2: shared responsibility<br>3: full autonomy	Q36_5_18<br>1: not allowed<br>2: under supervision<br>3: shared responsibility<br>4: full autonomy	Q10a_5_18<br>1: not allowed<br>2: under supervision<br>3: shared responsibility<br>4: full autonomy	Q10a_1_18<br>1: not allowed<br>2: under supervision<br>3: shared responsibility<br>4: full autonomy	Q10a_6_18<br>1: not allowed<br>2: under supervision<br>3: shared responsibility<br>4: full autonomy	Q10a_7_18<br>1: not allowed<br>2: under supervision<br>3: shared responsibility<br>4: full autonomy
other task within management of therapeutic and adverse effects of medicines: PLEASE SPECIFY	Q10a_4_19_1<br>Ticked = 1<br>Not ticked = 0<br>Q10a_4_19_TEXT	Q10a_2_19_1<br>Ticked = 1<br>Not ticked = 0<br>Q10a_2_19_TEXT	Q10a_3_19<br>1: under supervision<br>2: shared responsibility<br>3: full autonomy<br>Q10a_3_19_TEXT	Q36_5_19<br>1: not allowed<br>2: under supervision<br>3: shared responsibility<br>4: full autonomy<br>Q36_5_19_TEXT	Q10a_5_19<br>1: not allowed<br>2: under supervision<br>3: shared responsibility<br>4: full autonomy<br>Q10a_5_19_TEXT	Q10a_1_19<br>1: not allowed<br>2: under supervision<br>3: shared responsibility<br>4: full autonomy<br>Q10a_1_19_TEXT	Q10a_6_19<br>1: not allowed<br>2: under supervision<br>3: shared responsibility<br>4: full autonomy<br>Q10a_6_19_TEXT	Q10a_7_19<br>1: not allowed<br>2: under supervision<br>3: shared responsibility<br>4: full autonomy<br>Q10a_7_19_TEXT

Q11a/ RESPONSIBILITY 2: management of medicines adherence.

	**Task not relevant** within management of medicines adherence. (skip following columns, when this options is ticked)	**Task not allowed** for nurses within management of medicines adherence. (skip following columns, when this options is ticked)	Task allowed for **all levels** of nurses within management of medicines adherence. Specify the level of responsibility. (skip following columns, when this options is ticked)	Specify the level of responsibility for **level 4** nurses performing this task within management of medicines adherence Variable only applicable in Germany and the Netherlands	Specify the level of responsibility for **level 5** nurses performing this task within management of medicines adherence	Specify the level of responsibility for **level 6** nurses performing this task within management of medicines adherence	Specify the level of responsibility for **level 7** nurses performing this task within management of medicines adherence	Specify the level of responsibility for **level 8** nurses performing this task within management of medicines adherence
observation, documentation, registration, report	Q11a_4_1_1<br>Ticked = 1<br>Not ticked = 0	Q11a_2_1_1<br>Ticked = 1<br>Not ticked = 0	Q11a_3_1<br>1: under supervision<br>2: shared responsibility<br>3: full autonomy	Q42_5_1<br>1: not allowed<br>2: under supervision<br>3: shared responsibility	Q11a_5_1<br>1: not allowed<br>2: under supervision<br>3: shared responsibility	Q11a_1_1<br>1: not allowed<br>2: under supervision<br>3: shared responsibility	Q11a_6_1<br>1: not allowed<br>2: under supervision<br>3: shared responsibility	Q11a_7_1<br>1: not allowed<br>2: under supervision<br>3: shared responsibility

				4: full autonomy	4: full autonomy	4: full autonomy	4: full autonomy	4: full autonomy
assessing patients competences	Q11a_4_2_1<br>Ticked = 1<br>Not ticked = 0	Q11a_2_2_1<br>Ticked = 1<br>Not ticked = 0	Q11a_3_2<br>1: under supervision<br>2: shared responsibility<br>3: full autonomy	Q42_5_2<br>1: not allowed<br>2: under supervision<br>3: shared responsibility<br>4: full autonomy	Q11a_5_2<br>1: not allowed<br>2: under supervision<br>3: shared responsibility<br>4: full autonomy	Q11a_1_2<br>1: not allowed<br>2: under supervision<br>3: shared responsibility<br>4: full autonomy	Q11a_6_2<br>1: not allowed<br>2: under supervision<br>3: shared responsibility<br>4: full autonomy	Q11a_7_2<br>1: not allowed<br>2: under supervision<br>3: shared responsibility<br>4: full autonomy
assessment and addressing patient/family needs	Q11a_4_3_1<br>Ticked = 1<br>Not ticked = 0	Q11a_2_3_1<br>Ticked = 1<br>Not ticked = 0	Q11a_3_3<br>1: under supervision<br>2: shared responsibility<br>3: full autonomy	Q42_5_3<br>1: not allowed<br>2: under supervision<br>3: shared responsibility<br>4: full autonomy	Q11a_5_3<br>1: not allowed<br>2: under supervision<br>3: shared responsibility<br>4: full autonomy	Q11a_1_3<br>1: not allowed<br>2: under supervision<br>3: shared responsibility<br>4: full autonomy	Q11a_6_3<br>1: not allowed<br>2: under supervision<br>3: shared responsibility<br>4: full autonomy	Q11a_7_3<br>1: not allowed<br>2: under supervision<br>3: shared responsibility<br>4: full autonomy
recognising & preventing risks / complications / medication errors	Q11a_4_4_1<br>Ticked = 1<br>Not ticked = 0	Q11a_2_4_1<br>Ticked = 1<br>Not ticked = 0	Q11a_3_4<br>1: under supervision<br>2: shared responsibility<br>3: full autonomy	Q42_5_4<br>1: not allowed<br>2: under supervision<br>3: shared responsibility<br>4: full autonomy	Q11a_5_4<br>1: not allowed<br>2: under supervision<br>3: shared responsibility<br>4: full autonomy	Q11a_1_4<br>1: not allowed<br>2: under supervision<br>3: shared responsibility<br>4: full autonomy	Q11a_6_4<br>1: not allowed<br>2: under supervision<br>3: shared responsibility<br>4: full autonomy	Q11a_7_4<br>1: not allowed<br>2: under supervision<br>3: shared responsibility<br>4: full autonomy
identification, reporting and addressing contra-indications, drug related problems	Q11a_4_5_1<br>Ticked = 1<br>Not ticked = 0	Q11a_2_5_1<br>Ticked = 1<br>Not ticked = 0	Q11a_3_5<br>1: under supervision<br>2: shared responsibility<br>3: full autonomy	Q42_5_5<br>1: not allowed<br>2: under supervision<br>3: shared responsibility<br>4: full autonomy	Q11a_5_5<br>1: not allowed<br>2: under supervision<br>3: shared responsibility<br>4: full autonomy	Q11a_1_5<br>1: not allowed<br>2: under supervision<br>3: shared responsibility<br>4: full autonomy	Q11a_6_5<br>1: not allowed<br>2: under supervision<br>3: shared responsibility<br>4: full autonomy	Q11a_7_5<br>1: not allowed<br>2: under supervision<br>3: shared responsibility<br>4: full autonomy
follow-up	Q11a_4_6_1<br>Ticked = 1<br>Not ticked = 0	Q11a_2_6_1<br>Ticked = 1<br>Not ticked = 0	Q11a_3_6<br>1: under supervision<br>2: shared responsibility<br>3: full autonomy	Q42_5_6<br>1: not allowed<br>2: under supervision<br>3: shared responsibility<br>4: full autonomy	Q11a_5_6<br>1: not allowed<br>2: under supervision<br>3: shared responsibility<br>4: full autonomy	Q11a_1_6<br>1: not allowed<br>2: under supervision<br>3: shared responsibility<br>4: full autonomy	Q11a_6_6<br>1: not allowed<br>2: under supervision<br>3: shared responsibility<br>4: full autonomy	Q11a_7_6<br>1: not allowed<br>2: under supervision<br>3: shared responsibility<br>4: full autonomy
evidence based practice	Q11a_4_7_1<br>Ticked = 1<br>Not ticked = 0	Q11a_2_7_1<br>Ticked = 1<br>Not ticked = 0	Q11a_3_7<br>1: under supervision<br>2: shared responsibility<br>3: full autonomy	Q42_5_7<br>1: not allowed<br>2: under supervision<br>3: shared responsibility<br>4: full autonomy	Q11a_5_7<br>1: not allowed<br>2: under supervision<br>3: shared responsibility<br>4: full autonomy	Q11a_1_7<br>1: not allowed<br>2: under supervision<br>3: shared responsibility<br>4: full autonomy	Q11a_6_7<br>1: not allowed<br>2: under supervision<br>3: shared responsibility<br>4: full autonomy	Q11a_7_7<br>1: not allowed<br>2: under supervision<br>3: shared responsibility<br>4: full autonomy
decision making	Q11a_4_8_1<br>Ticked = 1<br>Not ticked = 0	Q11a_2_8_1<br>Ticked = 1<br>Not ticked = 0	Q11a_3_8<br>1: under supervision<br>2: shared responsibility<br>3: full autonomy	Q42_5_8<br>1: not allowed<br>2: under supervision<br>3: shared responsibility<br>4: full autonomy	Q11a_5_8<br>1: not allowed<br>2: under supervision<br>3: shared responsibility<br>4: full autonomy	Q11a_1_8<br>1: not allowed<br>2: under supervision<br>3: shared responsibility<br>4: full autonomy	Q11a_6_8<br>1: not allowed<br>2: under supervision<br>3: shared responsibility<br>4: full autonomy	Q11a_7_8<br>1: not allowed<br>2: under supervision<br>3: shared responsibility<br>4: full autonomy
communication /discussion with patient/family	Q11a_4_9_1<br>Ticked = 1<br>Not ticked = 0	Q11a_2_9_1<br>Ticked = 1<br>Not ticked = 0	Q11a_3_9<br>1: under supervision<br>2: shared responsibility<br>3: full autonomy	Q42_5_9<br>1: not allowed<br>2: under supervision<br>3: shared responsibility<br>4: full autonomy	Q11a_5_9<br>1: not allowed<br>2: under supervision<br>3: shared responsibility<br>4: full autonomy	Q11a_1_9<br>1: not allowed<br>2: under supervision<br>3: shared responsibility<br>4: full autonomy	Q11a_6_9<br>1: not allowed<br>2: under supervision<br>3: shared responsibility<br>4: full autonomy	Q11a_7_9<br>1: not allowed<br>2: under supervision<br>3: shared responsibility<br>4: full autonomy
detection of non-adherence, drug abuse/misuse	Q11a_4_10_1<br>Ticked = 1<br>Not ticked = 0	Q11a_2_10_1<br>Ticked = 1<br>Not ticked = 0	Q11a_3_10<br>1: under supervision<br>2: shared responsibility<br>3: full autonomy	Q42_5_10<br>1: not allowed<br>2: under supervision<br>3: shared responsibility<br>4: full autonomy	Q11a_5_10<br>1: not allowed<br>2: under supervision<br>3: shared responsibility<br>4: full autonomy	Q11a_1_10<br>1: not allowed<br>2: under supervision<br>3: shared responsibility<br>4: full autonomy	Q11a_6_10<br>1: not allowed<br>2: under supervision<br>3: shared responsibility<br>4: full autonomy	Q11a_7_10<br>1: not allowed<br>2: under supervision<br>3: shared responsibility<br>4: full autonomy
motivational interviewing	Q11a_4_11_1<br>Ticked = 1<br>Not ticked = 0	Q11a_2_11_1<br>Ticked = 1<br>Not ticked = 0	Q11a_3_11<br>1: under supervision<br>2: shared responsibility<br>3: full autonomy	Q42_5_11<br>1: not allowed<br>2: under supervision<br>3: shared responsibility	Q11a_5_11<br>1: not allowed<br>2: under supervision<br>3: shared responsibility	Q11a_1_11<br>1: not allowed<br>2: under supervision<br>3: shared responsibility	Q11a_6_11<br>1: not allowed<br>2: under supervision<br>3: shared responsibility	Q11a_7_11<br>1: not allowed<br>2: under supervision<br>3: shared responsibility

				4: full autonomy	4: full autonomy	4: full autonomy	4: full autonomy	4: full autonomy
Inter / intraprofessional referrals	**Q11a_4_12_1**<br>Ticked = 1<br>Not ticked = 0	**Q11a_2_12_1**<br>Ticked = 1<br>Not ticked = 0	**Q11a_3_12**<br>1: under supervision<br>2: shared responsibility<br>3: full autonomy	**Q42_5_12**<br>1: not allowed<br>2: under supervision<br>3: shared responsibility<br>4: full autonomy	**Q11a_5_12**<br>1: not allowed<br>2: under supervision<br>3: shared responsibility<br>4: full autonomy	**Q11a_1_12**<br>1: not allowed<br>2: under supervision<br>3: shared responsibility<br>4: full autonomy	**Q11a_6_12**<br>1: not allowed<br>2: under supervision<br>3: shared responsibility<br>4: full autonomy	**Q11a_7_12**<br>1: not allowed<br>2: under supervision<br>3: shared responsibility<br>4: full autonomy
facilitation of medication management	**Q11a_4_14_1**<br>Ticked = 1<br>Not ticked = 0	**Q11a_2_14_1**<br>Ticked = 1<br>Not ticked = 0	**Q11a_3_14**<br>1: under supervision<br>2: shared responsibility<br>3: full autonomy	**Q42_5_14**<br>1: not allowed<br>2: under supervision<br>3: shared responsibility<br>4: full autonomy	**Q11a_5_14**<br>1: not allowed<br>2: under supervision<br>3: shared responsibility<br>4: full autonomy	**Q11a_1_14**<br>1: not allowed<br>2: under supervision<br>3: shared responsibility<br>4: full autonomy	**Q11a_6_14**<br>1: not allowed<br>2: under supervision<br>3: shared responsibility<br>4: full autonomy	**Q11a_7_14**<br>1: not allowed<br>2: under supervision<br>3: shared responsibility<br>4: full autonomy
selfcare support and therapeutic education	**Q11a_4_17_1**<br>Ticked = 1<br>Not ticked = 0	**Q11a_2_17_1**<br>Ticked = 1<br>Not ticked = 0	**Q11a_3_17**<br>1: under supervision<br>2: shared responsibility<br>3: full autonomy	**Q42_5_17**<br>1: not allowed<br>2: under supervision<br>3: shared responsibility<br>4: full autonomy	**Q11a_5_17**<br>1: not allowed<br>2: under supervision<br>3: shared responsibility<br>4: full autonomy	**Q11a_1_17**<br>1: not allowed<br>2: under supervision<br>3: shared responsibility<br>4: full autonomy	**Q11a_6_17**<br>1: not allowed<br>2: under supervision<br>3: shared responsibility<br>4: full autonomy	**Q11a_7_17**<br>1: not allowed<br>2: under supervision<br>3: shared responsibility<br>4: full autonomy
advice (patient or other healthcare professional)	**Q11a_4_18_1**<br>Ticked = 1<br>Not ticked = 0	**Q11a_2_18_1**<br>Ticked = 1<br>Not ticked = 0	**Q11a_3_18**<br>1: under supervision<br>2: shared responsibility<br>3: full autonomy	**Q42_5_18**<br>1: not allowed<br>2: under supervision<br>3: shared responsibility<br>4: full autonomy	**Q11a_5_18**<br>1: not allowed<br>2: under supervision<br>3: shared responsibility<br>4: full autonomy	**Q11a_1_18**<br>1: not allowed<br>2: under supervision<br>3: shared responsibility<br>4: full autonomy	**Q11a_6_18**<br>1: not allowed<br>2: under supervision<br>3: shared responsibility<br>4: full autonomy	**Q11a_7_18**<br>1: not allowed<br>2: under supervision<br>3: shared responsibility<br>4: full autonomy
transitional care communication, inter / intraprofessional collaboration / communication including reporting, advising, informing, alerting and discussing	**Q11a_4_19_1**<br>Ticked = 1<br>Not ticked = 0	**Q11a_2_19_1**<br>Ticked = 1<br>Not ticked = 0	**Q11a_3_19**<br>1: under supervision<br>2: shared responsibility<br>3: full autonomy	**Q42_5_19**<br>1: not allowed<br>2: under supervision<br>3: shared responsibility<br>4: full autonomy	**Q11a_5_19**<br>1: not allowed<br>2: under supervision<br>3: shared responsibility<br>4: full autonomy	**Q11a_1_19**<br>1: not allowed<br>2: under supervision<br>3: shared responsibility<br>4: full autonomy	**Q11a_6_19**<br>1: not allowed<br>2: under supervision<br>3: shared responsibility<br>4: full autonomy	**Q11a_7_19**<br>1: not allowed<br>2: under supervision<br>3: shared responsibility<br>4: full autonomy
collegial mentoring	**Q11a_4_20_1**<br>Ticked = 1<br>Not ticked = 0	**Q11a_2_20_1**<br>Ticked = 1<br>Not ticked = 0	**Q11a_3_20**<br>1: under supervision<br>2: shared responsibility<br>3: full autonomy	**Q42_5_20**<br>1: not allowed<br>2: under supervision<br>3: shared responsibility<br>4: full autonomy	**Q11a_5_20**<br>1: not allowed<br>2: under supervision<br>3: shared responsibility<br>4: full autonomy	**Q11a_1_20**<br>1: not allowed<br>2: under supervision<br>3: shared responsibility<br>4: full autonomy	**Q11a_6_20**<br>1: not allowed<br>2: under supervision<br>3: shared responsibility<br>4: full autonomy	**Q11a_7_20**<br>1: not allowed<br>2: under supervision<br>3: shared responsibility<br>4: full autonomy
other task within management of medicines adherence:	**Q11a_4_21_1**<br>Ticked = 1<br>Not ticked = 0<br>**Q11a_4_21_T EXT**	**Q11a_2_21_1**<br>Ticked = 1<br>Not ticked = 0<br>**Q11a_2_21_T EXT**	**Q11a_3_21**<br>1: under supervision<br>2: shared responsibility<br>3: full autonomy<br>**Q11a_3_21_T EXT**	**Q42_5_21**<br>1: not allowed<br>2: under supervision<br>3: shared responsibility<br>4: full autonomy<br>**Q42_5_21_T EXT**	**Q11a_5_21**<br>1: not allowed<br>2: under supervision<br>3: shared responsibility<br>4: full autonomy<br>**Q11a_5_21_ TEXT**	**Q11a_1_21**<br>1: not allowed<br>2: under supervision<br>3: shared responsibility<br>4: full autonomy<br>**Q11a_1_21_ TEXT**	**Q11a_6_21**<br>1: not allowed<br>2: under supervision<br>3: shared responsibility<br>4: full autonomy<br>**Q11a_6_21_ TEXT**	**Q11a_7_21**<br>1: not allowed<br>2: under supervision<br>3: shared responsibility<br>4: full autonomy<br>**Q11a_7_21_ TEXT**

Q12a/ RESPONSIBILITY 3: management of patient medication self-management.

	**Task not relevant** within management of patient medication self-management.	**Task not allowed** for nurses within management of patient medication self-management. (skip following columns, when this options is ticked)	Task allowed for **all levels** of nurses within management of patient medication self-management. Specify the level of responsibility. (skip following columns, when this options is ticked)	Specify the level of responsibility for **level 4** nurses performing this task within patient medication self-management Variable only applicable in Germany and the Netherlands	Specify the level of responsibility for **level 5** nurses performing this task within patient medication self-management	Specify the level of responsibility for **level 6** nurses performing this task within patient medication self-management	Specify the level of responsibility for **level 7** nurses performing this task within patient medication self-management	Specify the level of responsibility for **level 8** nurses performing this task within patient medication self-management
observation, documentation, registration, report	**Q12a_4_1_1**<br>Ticked = 1<br>Not ticked = 0	**Q12a_2_1_1**<br>Ticked = 1<br>Not ticked = 0	**Q12a_3_1**<br>1: under supervision<br>2: shared responsibility<br>3: full autonomy	**Q41_5_1**<br>1: not allowed<br>2: under supervision<br>3: shared responsibility<br>4: full autonomy	**Q12a_5_1**<br>1: not allowed<br>2: under supervision<br>3: shared responsibility<br>4: full autonomy	**Q12a_1_1**<br>1: not allowed<br>2: under supervision<br>3: shared responsibility<br>4: full autonomy	**Q12a_6_1**<br>1: not allowed<br>2: under supervision<br>3: shared responsibility<br>4: full autonomy	**Q12a_7_1**<br>1: not allowed<br>2: under supervision<br>3: shared responsibility<br>4: full autonomy
assessing patients competences	**Q12a_4_2_1**<br>Ticked = 1<br>Not ticked = 0	**Q12a_2_2_1**<br>Ticked = 1<br>Not ticked = 0	**Q12a_3_2**<br>1: under supervision<br>2: shared responsibility<br>3: full autonomy	**Q41_5_2**<br>1: not allowed<br>2: under supervision<br>3: shared responsibility<br>4: full autonomy	**Q12a_5_2**<br>1: not allowed<br>2: under supervision<br>3: shared responsibility<br>4: full autonomy	**Q12a_1_2**<br>1: not allowed<br>2: under supervision<br>3: shared responsibility<br>4: full autonomy	**Q12a_6_2**<br>1: not allowed<br>2: under supervision<br>3: shared responsibility<br>4: full autonomy	**Q12a_7_2**<br>1: not allowed<br>2: under supervision<br>3: shared responsibility<br>4: full autonomy
assessment and addressing patient/family needs	**Q12a_4_3_1**<br>Ticked = 1<br>Not ticked = 0	**Q12a_2_3_1**<br>Ticked = 1<br>Not ticked = 0	**Q12a_3_3**<br>1: under supervision<br>2: shared responsibility<br>3: full autonomy	**Q41_5_3**<br>1: not allowed<br>2: under supervision<br>3: shared responsibility<br>4: full autonomy	**Q12a_5_3**<br>1: not allowed<br>2: under supervision<br>3: shared responsibility<br>4: full autonomy	**Q12a_1_3**<br>1: not allowed<br>2: under supervision<br>3: shared responsibility<br>4: full autonomy	**Q12a_6_3**<br>1: not allowed<br>2: under supervision<br>3: shared responsibility<br>4: full autonomy	**Q12a_7_3**<br>1: not allowed<br>2: under supervision<br>3: shared responsibility<br>4: full autonomy
recognising & preventing risks / complications / medication errors	**Q12a_4_4_1**<br>Ticked = 1<br>Not ticked = 0	**Q12a_2_4_1**<br>Ticked = 1<br>Not ticked = 0	**Q12a_3_4**<br>1: under supervision<br>2: shared responsibility<br>3: full autonomy	**Q41_5_4**<br>1: not allowed<br>2: under supervision<br>3: shared responsibility<br>4: full autonomy	**Q12a_5_4**<br>1: not allowed<br>2: under supervision<br>3: shared responsibility<br>4: full autonomy	**Q12a_1_4**<br>1: not allowed<br>2: under supervision<br>3: shared responsibility<br>4: full autonomy	**Q12a_6_4**<br>1: not allowed<br>2: under supervision<br>3: shared responsibility<br>4: full autonomy	**Q12a_7_4**<br>1: not allowed<br>2: under supervision<br>3: shared responsibility<br>4: full autonomy
identification, reporting and addressing contra-indications, drug related problems	**Q12a_4_5_1**<br>Ticked = 1<br>Not ticked = 0	**Q12a_2_5_1**<br>Ticked = 1<br>Not ticked = 0	**Q12a_3_5**<br>1: under supervision<br>2: shared responsibility<br>3: full autonomy	**Q41_5_5**<br>1: not allowed<br>2: under supervision<br>3: shared responsibility<br>4: full autonomy	**Q12a_5_5**<br>1: not allowed<br>2: under supervision<br>3: shared responsibility<br>4: full autonomy	**Q12a_1_5**<br>1: not allowed<br>2: under supervision<br>3: shared responsibility<br>4: full autonomy	**Q12a_6_5**<br>1: not allowed<br>2: under supervision<br>3: shared responsibility<br>4: full autonomy	**Q12a_7_5**<br>1: not allowed<br>2: under supervision<br>3: shared responsibility<br>4: full autonomy
follow-up	**Q12a_4_6_1**<br>Ticked = 1<br>Not ticked = 0	**Q12a_2_6_1**<br>Ticked = 1<br>Not ticked = 0	**Q12a_3_6**<br>1: under supervision<br>2: shared responsibility<br>3: full autonomy	**Q41_5_6**<br>1: not allowed<br>2: under supervision<br>3: shared responsibility<br>4: full autonomy	**Q12a_5_6**<br>1: not allowed<br>2: under supervision<br>3: shared responsibility<br>4: full autonomy	**Q12a_1_6**<br>1: not allowed<br>2: under supervision<br>3: shared responsibility<br>4: full autonomy	**Q12a_6_6**<br>1: not allowed<br>2: under supervision<br>3: shared responsibility<br>4: full autonomy	**Q12a_7_6**<br>1: not allowed<br>2: under supervision<br>3: shared responsibility<br>4: full autonomy
evidence based practice	**Q12a_4_7_1**<br>Ticked = 1<br>Not ticked = 0	**Q12a_2_7_1**<br>Ticked = 1<br>Not ticked = 0	**Q12a_3_7**<br>1: under supervision<br>2: shared responsibility<br>3: full autonomy	**Q41_5_7**<br>1: not allowed<br>2: under supervision<br>3: shared responsibility	**Q12a_5_7**<br>1: not allowed<br>2: under supervision<br>3: shared responsibility	**Q12a_1_7**<br>1: not allowed<br>2: under supervision<br>3: shared responsibility	**Q12a_6_7**<br>1: not allowed<br>2: under supervision<br>3: shared responsibility	**Q12a_7_7**<br>1: not allowed<br>2: under supervision<br>3: shared responsibility

				4: full autonomy	4: full autonomy	4: full autonomy	4: full autonomy	4: full autonomy
decision making	**Q12a_4_8_1**<br>Ticked = 1<br>Not ticked = 0	**Q12a_2_8_1**<br>Ticked = 1<br>Not ticked = 0	**Q12a_3_8**<br>1: under supervision<br>2: shared responsibility<br>3: full autonomy	**Q41_5_8**<br>1: not allowed<br>2: under supervision<br>3: shared responsibility<br>4: full autonomy	**Q12a_5_8**<br>1: not allowed<br>2: under supervision<br>3: shared responsibility<br>4: full autonomy	**Q12a_1_8**<br>1: not allowed<br>2: under supervision<br>3: shared responsibility<br>4: full autonomy	**Q12a_6_8**<br>1: not allowed<br>2: under supervision<br>3: shared responsibility<br>4: full autonomy	**Q12a_7_8**<br>1: not allowed<br>2: under supervision<br>3: shared responsibility<br>4: full autonomy
communication /discussion with patient/family	**Q12a_4_9_1**<br>Ticked = 1<br>Not ticked = 0	**Q12a_2_9_1**<br>Ticked = 1<br>Not ticked = 0	**Q12a_3_9**<br>1: under supervision<br>2: shared responsibility<br>3: full autonomy	**Q41_5_9**<br>1: not allowed<br>2: under supervision<br>3: shared responsibility<br>4: full autonomy	**Q12a_5_9**<br>1: not allowed<br>2: under supervision<br>3: shared responsibility<br>4: full autonomy	**Q12a_1_9**<br>1: not allowed<br>2: under supervision<br>3: shared responsibility<br>4: full autonomy	**Q12a_6_9**<br>1: not allowed<br>2: under supervision<br>3: shared responsibility<br>4: full autonomy	**Q12a_7_9**<br>1: not allowed<br>2: under supervision<br>3: shared responsibility<br>4: full autonomy
motivational interviewing	**Q12a_4_10_1**<br>Ticked = 1<br>Not ticked = 0	**Q12a_2_10_1**<br>Ticked = 1<br>Not ticked = 0	**Q12a_3_10**<br>1: under supervision<br>2: shared responsibility<br>3: full autonomy	**Q41_5_10**<br>1: not allowed<br>2: under supervision<br>3: shared responsibility<br>4: full autonomy	**Q12a_5_10**<br>1: not allowed<br>2: under supervision<br>3: shared responsibility<br>4: full autonomy	**Q12a_1_10**<br>1: not allowed<br>2: under supervision<br>3: shared responsibility<br>4: full autonomy	**Q12a_6_10**<br>1: not allowed<br>2: under supervision<br>3: shared responsibility<br>4: full autonomy	**Q12a_7_10**<br>1: not allowed<br>2: under supervision<br>3: shared responsibility<br>4: full autonomy
Inter / intraprofessional referrals	**Q12a_4_11_1**<br>Ticked = 1<br>Not ticked = 0	**Q12a_2_11_1**<br>Ticked = 1<br>Not ticked = 0	**Q12a_3_11**<br>1: under supervision<br>2: shared responsibility<br>3: full autonomy	**Q41_5_11**<br>1: not allowed<br>2: under supervision<br>3: shared responsibility<br>4: full autonomy	**Q12a_5_11**<br>1: not allowed<br>2: under supervision<br>3: shared responsibility<br>4: full autonomy	**Q12a_1_11**<br>1: not allowed<br>2: under supervision<br>3: shared responsibility<br>4: full autonomy	**Q12a_6_11**<br>1: not allowed<br>2: under supervision<br>3: shared responsibility<br>4: full autonomy	**Q12a_7_11**<br>1: not allowed<br>2: under supervision<br>3: shared responsibility<br>4: full autonomy
facilitation of medication management	**Q12a_4_13_1**<br>Ticked = 1<br>Not ticked = 0	**Q12a_2_13_1**<br>Ticked = 1<br>Not ticked = 0	**Q12a_3_13**<br>1: under supervision<br>2: shared responsibility<br>3: full autonomy	**Q41_5_13**<br>1: not allowed<br>2: under supervision<br>3: shared responsibility<br>4: full autonomy	**Q12a_5_13**<br>1: not allowed<br>2: under supervision<br>3: shared responsibility<br>4: full autonomy	**Q12a_1_13**<br>1: not allowed<br>2: under supervision<br>3: shared responsibility<br>4: full autonomy	**Q12a_6_13**<br>1: not allowed<br>2: under supervision<br>3: shared responsibility<br>4: full autonomy	**Q12a_7_13**<br>1: not allowed<br>2: under supervision<br>3: shared responsibility<br>4: full autonomy
selfcare support and therapeutic education	**Q12a_4_16_1**<br>Ticked = 1<br>Not ticked = 0	**Q12a_2_16_1**<br>Ticked = 1<br>Not ticked = 0	**Q12a_3_16**<br>1: under supervision<br>2: shared responsibility<br>3: full autonomy	**Q41_5_16**<br>1: not allowed<br>2: under supervision<br>3: shared responsibility<br>4: full autonomy	**Q12a_5_16**<br>1: not allowed<br>2: under supervision<br>3: shared responsibility<br>4: full autonomy	**Q12a_1_16**<br>1: not allowed<br>2: under supervision<br>3: shared responsibility<br>4: full autonomy	**Q12a_6_16**<br>1: not allowed<br>2: under supervision<br>3: shared responsibility<br>4: full autonomy	**Q12a_7_16**<br>1: not allowed<br>2: under supervision<br>3: shared responsibility<br>4: full autonomy
advice (patient or other healthcare professional)	**Q12a_4_17_1**<br>Ticked = 1<br>Not ticked = 0	**Q12a_2_17_1**<br>Ticked = 1<br>Not ticked = 0	**Q12a_3_17**<br>1: under supervision<br>2: shared responsibility<br>3: full autonomy	**Q41_5_17**<br>1: not allowed<br>2: under supervision<br>3: shared responsibility<br>4: full autonomy	**Q12a_5_17**<br>1: not allowed<br>2: under supervision<br>3: shared responsibility<br>4: full autonomy	**Q12a_1_17**<br>1: not allowed<br>2: under supervision<br>3: shared responsibility<br>4: full autonomy	**Q12a_6_17**<br>1: not allowed<br>2: under supervision<br>3: shared responsibility<br>4: full autonomy	**Q12a_7_17**<br>1: not allowed<br>2: under supervision<br>3: shared responsibility<br>4: full autonomy
transitional care communication, inter / intraprofessional collaboration / communication including reporting, advising, informing, alerting and discussing	**Q12a_4_18_1**<br>Ticked = 1<br>Not ticked = 0	**Q12a_2_18_1**<br>Ticked = 1<br>Not ticked = 0	**Q12a_3_18**<br>1: under supervision<br>2: shared responsibility<br>3: full autonomy	**Q41_5_18**<br>1: not allowed<br>2: under supervision<br>3: shared responsibility<br>4: full autonomy	**Q12a_5_18**<br>1: not allowed<br>2: under supervision<br>3: shared responsibility<br>4: full autonomy	**Q12a_1_18**<br>1: not allowed<br>2: under supervision<br>3: shared responsibility<br>4: full autonomy	**Q12a_6_18**<br>1: not allowed<br>2: under supervision<br>3: shared responsibility<br>4: full autonomy	**Q12a_7_18**<br>1: not allowed<br>2: under supervision<br>3: shared responsibility<br>4: full autonomy
collegial mentoring	**Q12a_4_19_1**<br>Ticked = 1<br>Not ticked = 0	**Q12a_2_19_1**<br>Ticked = 1<br>Not ticked = 0	**Q12a_3_19**<br>1: under supervision<br>2: shared responsibility<br>3: full autonomy	**Q41_5_19**<br>1: not allowed<br>2: under supervision<br>3: shared responsibility<br>4: full autonomy	**Q12a_5_19**<br>1: not allowed<br>2: under supervision<br>3: shared responsibility<br>4: full autonomy	**Q12a_1_19**<br>1: not allowed<br>2: under supervision<br>3: shared responsibility<br>4: full autonomy	**Q12a_6_19**<br>1: not allowed<br>2: under supervision<br>3: shared responsibility<br>4: full autonomy	**Q12a_7_19**<br>1: not allowed<br>2: under supervision<br>3: shared responsibility<br>4: full autonomy

other task within management of patient medication self-management:	**Q12a_4_20_1**<br>Ticked = 1<br>Not ticked = 0<br>**Q12a_4_20_TEXT**	**Q12a_2_20_1**<br>Ticked = 1<br>Not ticked = 0<br>**Q12a_2_20_TEXT**	**Q12a_3_20**<br>1: under supervision<br>2: shared responsibility<br>3: full autonomy<br>**Q12a_3_20_TEXT**	**Q41_5_20**<br>1: not allowed<br>2: under supervision<br>3: shared responsibility<br>4: full autonomy<br>**Q41_5_20_TEXT**	**Q12a_5_20**<br>1: not allowed<br>2: under supervision<br>3: shared responsibility<br>4: full autonomy<br>**Q12a_5_20_TEXT**	**Q12a_1_20**<br>1: not allowed<br>2: under supervision<br>3: shared responsibility<br>4: full autonomy<br>**Q12a_1_20_TEXT**	**Q12a_6_20**<br>1: not allowed<br>2: under supervision<br>3: shared responsibility<br>4: full autonomy<br>**Q12a_6_20_TEXT**	**Q12a_7_20**<br>1: not allowed<br>2: under supervision<br>3: shared responsibility<br>4: full autonomy<br>**Q12a_7_20_TEXT**

Q13a/ RESPONSIBILITY 4: management of patient education and information.

	**Task not relevant** within management of patient education and information. (skip following columns, when this options is ticked)	**Task not allowed** for nurses within management of patient education and information. (skip following columns, when this options is ticked)	Task allowed for **all levels** of nurses within management of patient education and information. Specify the level of responsibility. (skip following columns, when this options is ticked)	Specify the level of responsibility for **level 4** nurses performing this task within management of patient education and information Variable only applicable in Germany and the Netherlands	Specify the level of responsibility for **level 5** nurses performing this task within management of patient education and information	Specify the level of responsibility for **level 6** nurses performing this task within management of patient education and information	Specify the level of responsibility for **level 7** nurses performing this task within management of patient education and information	Specify the level of responsibility for **level 8** nurses performing this task within management of patient education and information
observation, documentation, registration, report	**Q13a_4_1_1**<br>Ticked = 1<br>Not ticked = 0	**Q13a_2_1_1**<br>Ticked = 1<br>Not ticked = 0	**Q13a_3_1**<br>1: under supervision<br>2: shared responsibility<br>3: full autonomy	**Q40_5_1**<br>1: not allowed<br>2: under supervision<br>3: shared responsibility<br>4: full autonomy	**Q13a_5_1**<br>1: not allowed<br>2: under supervision<br>3: shared responsibility<br>4: full autonomy	**Q13a_1_1**<br>1: not allowed<br>2: under supervision<br>3: shared responsibility<br>4: full autonomy	**Q12a_6_1**<br>1: not allowed<br>2: under supervision<br>3: shared responsibility<br>4: full autonomy	**Q12a_7_1**<br>1: not allowed<br>2: under supervision<br>3: shared responsibility<br>4: full autonomy
assessing patients competences	**Q13a_4_2_1**<br>Ticked = 1<br>Not ticked = 0	**Q13a_2_2_1**<br>Ticked = 1<br>Not ticked = 0	**Q13a_3_2**<br>1: under supervision<br>2: shared responsibility<br>3: full autonomy	**Q40_5_2**<br>1: not allowed<br>2: under supervision<br>3: shared responsibility<br>4: full autonomy	**Q13a_5_2**<br>1: not allowed<br>2: under supervision<br>3: shared responsibility<br>4: full autonomy	**Q13a_1_2**<br>1: not allowed<br>2: under supervision<br>3: shared responsibility<br>4: full autonomy	**Q13a_6_2**<br>1: not allowed<br>2: under supervision<br>3: shared responsibility<br>4: full autonomy	**Q12a_7_2**<br>1: not allowed<br>2: under supervision<br>3: shared responsibility<br>4: full autonomy
assessment and addressing patient/family needs	**Q13a_4_3_1**<br>Ticked = 1<br>Not ticked = 0	**Q13a_2_3_1**<br>Ticked = 1<br>Not ticked = 0	**Q13a_3_3**<br>1: under supervision<br>2: shared responsibility<br>3: full autonomy	**Q40_5_3**<br>1: not allowed<br>2: under supervision<br>3: shared responsibility<br>4: full autonomy	**Q13a_5_3**<br>1: not allowed<br>2: under supervision<br>3: shared responsibility<br>4: full autonomy	**Q13a_1_3**<br>1: not allowed<br>2: under supervision<br>3: shared responsibility<br>4: full autonomy	**Q12a_6_3**<br>1: not allowed<br>2: under supervision<br>3: shared responsibility<br>4: full autonomy	**Q12a_7_3**<br>1: not allowed<br>2: under supervision<br>3: shared responsibility<br>4: full autonomy
follow-up	**Q13a_4_4_1**<br>Ticked = 1<br>Not ticked = 0	**Q13a_2_4_1**<br>Ticked = 1<br>Not ticked = 0	**Q13a_3_4**<br>1: under supervision<br>2: shared responsibility<br>3: full autonomy	**Q40_5_4**<br>1: not allowed<br>2: under supervision<br>3: shared responsibility<br>4: full autonomy	**Q13a_5_4**<br>1: not allowed<br>2: under supervision<br>3: shared responsibility<br>4: full autonomy	**Q13a_1_4**<br>1: not allowed<br>2: under supervision<br>3: shared responsibility<br>4: full autonomy	**Q12a_6_4**<br>1: not allowed<br>2: under supervision<br>3: shared responsibility<br>4: full autonomy	**Q12a_7_4**<br>1: not allowed<br>2: under supervision<br>3: shared responsibility<br>4: full autonomy
evidence based practice	**Q13a_4_5_1**	**Q13a_2_5_1**	**Q13a_3_5**	**Q40_5_5**	**Q13a_5_5**	**Q13a_1_5**	**Q13a_6_5**	**Q13a_7_5**

	Ticked = 1<br>Not ticked = 0	Ticked = 1<br>Not ticked = 0	1: under supervision<br>2: shared responsibility<br>3: full autonomy	1: not allowed<br>2: under supervision<br>3: shared responsibility<br>4: full autonomy	1: not allowed<br>2: under supervision<br>3: shared responsibility<br>4: full autonomy	1: not allowed<br>2: under supervision<br>3: shared responsibility<br>4: full autonomy	1: not allowed<br>2: under supervision<br>3: shared responsibility<br>4: full autonomy	1: not allowed<br>2: under supervision<br>3: shared responsibility<br>4: full autonomy
decision making	**Q13a_4_6_1**<br>Ticked = 1<br>Not ticked = 0	**Q13a_2_6_1**<br>Ticked = 1<br>Not ticked = 0	**Q13a_3_6**<br>1: under supervision<br>2: shared responsibility<br>3: full autonomy	**Q40_5_6**<br>1: not allowed<br>2: under supervision<br>3: shared responsibility<br>4: full autonomy	**Q13a_5_6**<br>1: not allowed<br>2: under supervision<br>3: shared responsibility<br>4: full autonomy	**Q13a_1_6**<br>1: not allowed<br>2: under supervision<br>3: shared responsibility<br>4: full autonomy	**Q13a_6_6**<br>1: not allowed<br>2: under supervision<br>3: shared responsibility<br>4: full autonomy	**Q13a_7_6**<br>1: not allowed<br>2: under supervision<br>3: shared responsibility<br>4: full autonomy
communication /discussion with patient/family	**Q13a_4_7_1**<br>Ticked = 1<br>Not ticked = 0	**Q13a_2_7_1**<br>Ticked = 1<br>Not ticked = 0	**Q13a_3_7**<br>1: under supervision<br>2: shared responsibility<br>3: full autonomy	**Q40_5_7**<br>1: not allowed<br>2: under supervision<br>3: shared responsibility<br>4: full autonomy	**Q13a_5_7**<br>1: not allowed<br>2: under supervision<br>3: shared responsibility<br>4: full autonomy	**Q13a_1_7**<br>1: not allowed<br>2: under supervision<br>3: shared responsibility<br>4: full autonomy	**Q13a_6_7**<br>1: not allowed<br>2: under supervision<br>3: shared responsibility<br>4: full autonomy	**Q13a_7_7**<br>1: not allowed<br>2: under supervision<br>3: shared responsibility<br>4: full autonomy
motivational interviewing	**Q13a_4_8_1**<br>Ticked = 1<br>Not ticked = 0	**Q13a_2_8_1**<br>Ticked = 1<br>Not ticked = 0	**Q13a_3_8**<br>1: under supervision<br>2: shared responsibility<br>3: full autonomy	**Q40_5_8**<br>1: not allowed<br>2: under supervision<br>3: shared responsibility<br>4: full autonomy	**Q13a_5_8**<br>1: not allowed<br>2: under supervision<br>3: shared responsibility<br>4: full autonomy	**Q13a_1_8**<br>1: not allowed<br>2: under supervision<br>3: shared responsibility<br>4: full autonomy	**Q13a_6_8**<br>1: not allowed<br>2: under supervision<br>3: shared responsibility<br>4: full autonomy	**Q13a_7_8**<br>1: not allowed<br>2: under supervision<br>3: shared responsibility<br>4: full autonomy
Inter / intraprofessional referrals	**Q13a_4_9_1**<br>Ticked = 1<br>Not ticked = 0	**Q13a_2_9_1**<br>Ticked = 1<br>Not ticked = 0	**Q13a_3_9**<br>1: under supervision<br>2: shared responsibility<br>3: full autonomy	**Q40_5_9**<br>1: not allowed<br>2: under supervision<br>3: shared responsibility<br>4: full autonomy	**Q13a_5_9**<br>1: not allowed<br>2: under supervision<br>3: shared responsibility<br>4: full autonomy	**Q13a_1_9**<br>1: not allowed<br>2: under supervision<br>3: shared responsibility<br>4: full autonomy	**Q13a_6_9**<br>1: not allowed<br>2: under supervision<br>3: shared responsibility<br>4: full autonomy	**Q13a_7_9**<br>1: not allowed<br>2: under supervision<br>3: shared responsibility<br>4: full autonomy
facilitation of medication management	**Q13a_4_11_1**<br>Ticked = 1<br>Not ticked = 0	**Q13a_2_11_<br>1**<br>Ticked = 1<br>Not ticked = 0	**Q13a_3_11**<br>1: under supervision<br>2: shared responsibility<br>3: full autonomy	**Q40_5_11**<br>1: not allowed<br>2: under supervision<br>3: shared responsibility<br>4: full autonomy	**Q13a_5_11**<br>1: not allowed<br>2: under supervision<br>3: shared responsibility<br>4: full autonomy	**Q13a_1_11**<br>1: not allowed<br>2: under supervision<br>3: shared responsibility<br>4: full autonomy	**Q13a_6_11**<br>1: not allowed<br>2: under supervision<br>3: shared responsibility<br>4: full autonomy	**Q13a_7_11**<br>1: not allowed<br>2: under supervision<br>3: shared responsibility<br>4: full autonomy
selfcare support and therapeutic education	**Q13a_4_14_1**<br>Ticked = 1<br>Not ticked = 0	**Q13a_2_14_<br>1**<br>Ticked = 1<br>Not ticked = 0	**Q13a_3_14**<br>1: under supervision<br>2: shared responsibility<br>3: full autonomy	**Q40_5_14**<br>1: not allowed<br>2: under supervision<br>3: shared responsibility<br>4: full autonomy	**Q13a_5_14**<br>1: not allowed<br>2: under supervision<br>3: shared responsibility<br>4: full autonomy	**Q13a_1_14**<br>1: not allowed<br>2: under supervision<br>3: shared responsibility<br>4: full autonomy	**Q13a_6_14**<br>1: not allowed<br>2: under supervision<br>3: shared responsibility<br>4: full autonomy	**Q13a_7_14**<br>1: not allowed<br>2: under supervision<br>3: shared responsibility<br>4: full autonomy
advice (patient or other healthcare professional)	**Q13a_4_15_1**<br>Ticked = 1<br>Not ticked = 0	**Q13a_2_15_<br>1**<br>Ticked = 1<br>Not ticked = 0	**Q13a_3_15**<br>1: under supervision<br>2: shared responsibility<br>3: full autonomy	**Q40_5_15**<br>1: not allowed<br>2: under supervision<br>3: shared responsibility<br>4: full autonomy	**Q13a_5_15**<br>1: not allowed<br>2: under supervision<br>3: shared responsibility<br>4: full autonomy	**Q13a_1_15**<br>1: not allowed<br>2: under supervision<br>3: shared responsibility<br>4: full autonomy	**Q13a_6_15**<br>1: not allowed<br>2: under supervision<br>3: shared responsibility<br>4: full autonomy	**Q13a_7_15**<br>1: not allowed<br>2: under supervision<br>3: shared responsibility<br>4: full autonomy
transitional care communication, inter / intraprofessional collaboration / communication including reporting, advising, informing, alerting and discussing	**Q13a_4_16_1**<br>Ticked = 1<br>Not ticked = 0	**Q13a_2_16_<br>1**<br>Ticked = 1<br>Not ticked = 0	**Q13a_3_16**<br>1: under supervision<br>2: shared responsibility<br>3: full autonomy	**Q40_5_16**<br>1: not allowed<br>2: under supervision<br>3: shared responsibility<br>4: full autonomy	**Q13a_5_16**<br>1: not allowed<br>2: under supervision<br>3: shared responsibility<br>4: full autonomy	**Q13a_1_16**<br>1: not allowed<br>2: under supervision<br>3: shared responsibility<br>4: full autonomy	**Q13a_6_16**<br>1: not allowed<br>2: under supervision<br>3: shared responsibility<br>4: full autonomy	**Q13a_7_16**<br>1: not allowed<br>2: under supervision<br>3: shared responsibility<br>4: full autonomy
collegial mentoring	**Q13a_4_17_1**<br>Ticked = 1	**Q13a_2_17_<br>1**	**Q13a_3_17**<br>1: under supervision<br>2: shared responsibility	**Q40_5_17**<br>1: not allowed<br>2: under supervision	**Q13a_5_17**<br>1: not allowed<br>2: under supervision	**Q13a_1_17**<br>1: not allowed<br>2: under supervision	**Q13a_6_17**<br>1: not allowed<br>2: under supervision	**Q13a_7_17**<br>1: not allowed<br>2: under supervision

	Not ticked = 0	Ticked = 1<br>Not ticked = 0	3: full autonomy	3: shared responsibility<br>4: full autonomy	3: shared responsibility<br>4: full autonomy	3: shared responsibility<br>4: full autonomy	3: shared responsibility<br>4: full autonomy	3: shared responsibility<br>4: full autonomy
other task within management of patient education and information:	**Q13a_4_18_1**<br>Ticked = 1<br>Not ticked = 0<br>**Q13a_4_18_TEXT**	**Q13a_2_18_1**<br>Ticked = 1<br>Not ticked = 0<br>**Q13a_2_18_TEXT**	**Q13a_3_18**<br>1: under supervision<br>2: shared responsibility<br>3: full autonomy<br>**Q13a_2_18_TEXT**	**Q40_5_18**<br>1: not allowed<br>2: under supervision<br>3: shared responsibility<br>4: full autonomy<br>**Q40_5_18_TEXT**	**Q13a_5_18**<br>1: not allowed<br>2: under supervision<br>3: shared responsibility<br>4: full autonomy<br>**Q13a_5_18_TEXT**	**Q13a_1_18**<br>1: not allowed<br>2: under supervision<br>3: shared responsibility<br>4: full autonomy<br>**Q13a_1_18_TEXT**	**Q13a_6_18**<br>1: not allowed<br>2: under supervision<br>3: shared responsibility<br>4: full autonomy<br>**Q13a_6_18_TEXT**	**Q13a_7_18**<br>1: not allowed<br>2: under supervision<br>3: shared responsibility<br>4: full autonomy<br>**Q13a_7_18_TEXT**

Q14a/ RESPONSIBILITY 5: prescription management.

	**Task not relevant** within prescription management. (skip following columns, when this options is ticked)	**Task not allowed** for nurses within prescription management. (skip following columns, when this options is ticked)	Task allowed for **all levels** of nurses within prescription management. Specify the level of responsibility. (skip following columns, when this options is ticked)	Specify the level of responsibility for **level 4** nurses performing this task within prescription management Variable only applicable in Germany and the Netherlands	Specify the level of responsibility for **level 5** nurses performing this task within prescription management	Specify the level of responsibility for **level 6** nurses performing this task within prescription management	Specify the level of responsibility for **level 7** nurses performing this task within prescription management	Specify the level of responsibility for **level 8** nurses performing this task within prescription management
observation, documentation, registration, report	**Q14a_4_1_1**<br>Ticked = 1<br>Not ticked = 0	**Q14a_2_1_1**<br>Ticked = 1<br>Not ticked = 0	**Q14a_3_1**<br>1: under supervision<br>2: shared responsibility<br>3: full autonomy	**Q39_5_1**<br>1: not allowed<br>2: under supervision<br>3: shared responsibility<br>4: full autonomy	**Q14a_5_1**<br>1: not allowed<br>2: under supervision<br>3: shared responsibility<br>4: full autonomy	**Q14a_1_1**<br>1: not allowed<br>2: under supervision<br>3: shared responsibility<br>4: full autonomy	**Q14a_6_1**<br>1: not allowed<br>2: under supervision<br>3: shared responsibility<br>4: full autonomy	**Q14a_7_1**<br>1: not allowed<br>2: under supervision<br>3: shared responsibility<br>4: full autonomy
assessing patients competences	**Q14a_4_2_1**<br>Ticked = 1<br>Not ticked = 0	**Q14a_2_2_1**<br>Ticked = 1<br>Not ticked = 0	**Q14a_3_2**<br>1: under supervision<br>2: shared responsibility<br>3: full autonomy	**Q39_5_2**<br>1: not allowed<br>2: under supervision<br>3: shared responsibility<br>4: full autonomy	**Q14a_5_2**<br>1: not allowed<br>2: under supervision<br>3: shared responsibility<br>4: full autonomy	**Q14a_1_2**<br>1: not allowed<br>2: under supervision<br>3: shared responsibility<br>4: full autonomy	**Q14a_6_2**<br>1: not allowed<br>2: under supervision<br>3: shared responsibility<br>4: full autonomy	**Q14a_7_2**<br>1: not allowed<br>2: under supervision<br>3: shared responsibility<br>4: full autonomy
assessment and addressing patient/family needs	**Q14a_4_4_1**<br>Ticked = 1<br>Not ticked = 0	**Q14a_2_4_1**<br>Ticked = 1<br>Not ticked = 0	**Q14a_3_4**<br>1: under supervision<br>2: shared responsibility<br>3: full autonomy	**Q39_5_4**<br>1: not allowed<br>2: under supervision<br>3: shared responsibility<br>4: full autonomy	**Q14a_5_4**<br>1: not allowed<br>2: under supervision<br>3: shared responsibility<br>4: full autonomy	**Q14a_1_4**<br>1: not allowed<br>2: under supervision<br>3: shared responsibility<br>4: full autonomy	**Q14a_6_4**<br>1: not allowed<br>2: under supervision<br>3: shared responsibility<br>4: full autonomy	**Q14a_7_4**<br>1: not allowed<br>2: under supervision<br>3: shared responsibility<br>4: full autonomy
recognising & preventing risks / complications / medication errors	**Q14a_4_5_1**<br>Ticked = 1<br>Not ticked = 0	**Q14a_2_5_1**<br>Ticked = 1<br>Not ticked = 0	**Q14a_3_5**<br>1: under supervision<br>2: shared responsibility<br>3: full autonomy	**Q39_5_5**<br>1: not allowed<br>2: under supervision<br>3: shared responsibility<br>4: full autonomy	**Q14a_5_5**<br>1: not allowed<br>2: under supervision<br>3: shared responsibility<br>4: full autonomy	**Q14a_1_5**<br>1: not allowed<br>2: under supervision<br>3: shared responsibility<br>4: full autonomy	**Q14a_6_5**<br>1: not allowed<br>2: under supervision<br>3: shared responsibility<br>4: full autonomy	**Q14a_7_5**<br>1: not allowed<br>2: under supervision<br>3: shared responsibility<br>4: full autonomy

identification, reporting and addressing contra-indications, drug related problems	Q14a_4_6_1<br>Ticked = 1<br>Not ticked = 0	Q14a_2_6_1<br>Ticked = 1<br>Not ticked = 0	Q14a_3_6<br>1: under supervision<br>2: shared responsibility<br>3: full autonomy	Q39_5_6<br>1: not allowed<br>2: under supervision<br>3: shared responsibility<br>4: full autonomy	Q14a_5_6<br>1: not allowed<br>2: under supervision<br>3: shared responsibility<br>4: full autonomy	Q14a_1_6<br>1: not allowed<br>2: under supervision<br>3: shared responsibility<br>4: full autonomy	Q14a_6_6<br>1: not allowed<br>2: under supervision<br>3: shared responsibility<br>4: full autonomy	Q14a_7_6<br>1: not allowed<br>2: under supervision<br>3: shared responsibility<br>4: full autonomy
follow-up	Q14a_4_7_1<br>Ticked = 1<br>Not ticked = 0	Q14a_2_7_1<br>Ticked = 1<br>Not ticked = 0	Q14a_3_7<br>1: under supervision<br>2: shared responsibility<br>3: full autonomy	Q39_5_7<br>1: not allowed<br>2: under supervision<br>3: shared responsibility<br>4: full autonomy	Q14a_5_7<br>1: not allowed<br>2: under supervision<br>3: shared responsibility<br>4: full autonomy	Q14a_1_7<br>1: not allowed<br>2: under supervision<br>3: shared responsibility<br>4: full autonomy	Q14a_6_7<br>1: not allowed<br>2: under supervision<br>3: shared responsibility<br>4: full autonomy	Q14a_7_7<br>1: not allowed<br>2: under supervision<br>3: shared responsibility<br>4: full autonomy
evidence based practice	Q14a_4_8_1<br>Ticked = 1<br>Not ticked = 0	Q14a_2_8_1<br>Ticked = 1<br>Not ticked = 0	Q14a_3_8<br>1: under supervision<br>2: shared responsibility<br>3: full autonomy	Q39_5_8<br>1: not allowed<br>2: under supervision<br>3: shared responsibility<br>4: full autonomy	Q14a_5_8<br>1: not allowed<br>2: under supervision<br>3: shared responsibility<br>4: full autonomy	Q14a_1_8<br>1: not allowed<br>2: under supervision<br>3: shared responsibility<br>4: full autonomy	Q14a_6_8<br>1: not allowed<br>2: under supervision<br>3: shared responsibility<br>4: full autonomy	Q14a_7_8<br>1: not allowed<br>2: under supervision<br>3: shared responsibility<br>4: full autonomy
decision making	Q14a_4_9_1<br>Ticked = 1<br>Not ticked = 0	Q14a_2_9_1<br>Ticked = 1<br>Not ticked = 0	Q14a_3_9<br>1: under supervision<br>2: shared responsibility<br>3: full autonomy	Q39_5_9<br>1: not allowed<br>2: under supervision<br>3: shared responsibility<br>4: full autonomy	Q14a_5_9<br>1: not allowed<br>2: under supervision<br>3: shared responsibility<br>4: full autonomy	Q14a_1_9<br>1: not allowed<br>2: under supervision<br>3: shared responsibility<br>4: full autonomy	Q14a_6_9<br>1: not allowed<br>2: under supervision<br>3: shared responsibility<br>4: full autonomy	Q14a_7_9<br>1: not allowed<br>2: under supervision<br>3: shared responsibility<br>4: full autonomy
communication /discussion with patient/family	Q14a_4_10_1<br>Ticked = 1<br>Not ticked = 0	Q14a_2_10_1<br>Ticked = 1<br>Not ticked = 0	Q14a_3_10<br>1: under supervision<br>2: shared responsibility<br>3: full autonomy	Q39_5_10<br>1: not allowed<br>2: under supervision<br>3: shared responsibility<br>4: full autonomy	Q14a_5_10<br>1: not allowed<br>2: under supervision<br>3: shared responsibility<br>4: full autonomy	Q14a_1_10<br>1: not allowed<br>2: under supervision<br>3: shared responsibility<br>4: full autonomy	Q14a_6_10<br>1: not allowed<br>2: under supervision<br>3: shared responsibility<br>4: full autonomy	Q14a_7_10<br>1: not allowed<br>2: under supervision<br>3: shared responsibility<br>4: full autonomy
Inter / intraprofessional referrals	Q14a_4_11_1<br>Ticked = 1<br>Not ticked = 0	Q14a_2_11_1<br>Ticked = 1<br>Not ticked = 0	Q14a_3_11<br>1: under supervision<br>2: shared responsibility<br>3: full autonomy	Q39_5_11<br>1: not allowed<br>2: under supervision<br>3: shared responsibility<br>4: full autonomy	Q14a_5_11<br>1: not allowed<br>2: under supervision<br>3: shared responsibility<br>4: full autonomy	Q14a_1_11<br>1: not allowed<br>2: under supervision<br>3: shared responsibility<br>4: full autonomy	Q14a_6_11<br>1: not allowed<br>2: under supervision<br>3: shared responsibility<br>4: full autonomy	Q14a_7_11<br>1: not allowed<br>2: under supervision<br>3: shared responsibility<br>4: full autonomy
facilitation of medication management	Q14a_4_13_1<br>Ticked = 1<br>Not ticked = 0	Q14a_2_13_1<br>Ticked = 1<br>Not ticked = 0	Q14a_3_13<br>1: under supervision<br>2: shared responsibility<br>3: full autonomy	Q39_5_13<br>1: not allowed<br>2: under supervision<br>3: shared responsibility<br>4: full autonomy	Q14a_5_13<br>1: not allowed<br>2: under supervision<br>3: shared responsibility<br>4: full autonomy	Q14a_1_13<br>1: not allowed<br>2: under supervision<br>3: shared responsibility<br>4: full autonomy	Q14a_6_13<br>1: not allowed<br>2: under supervision<br>3: shared responsibility<br>4: full autonomy	Q14a_7_13<br>1: not allowed<br>2: under supervision<br>3: shared responsibility<br>4: full autonomy
advice (patient or other healthcare professional)	Q14a_4_15_1<br>Ticked = 1<br>Not ticked = 0	Q14a_2_15_1<br>Ticked = 1<br>Not ticked = 0	Q14a_3_15<br>1: under supervision<br>2: shared responsibility<br>3: full autonomy	Q39_5_15<br>1: not allowed<br>2: under supervision<br>3: shared responsibility<br>4: full autonomy	Q14a_5_15<br>1: not allowed<br>2: under supervision<br>3: shared responsibility<br>4: full autonomy	Q14a_1_15<br>1: not allowed<br>2: under supervision<br>3: shared responsibility<br>4: full autonomy	Q14a_6_15<br>1: not allowed<br>2: under supervision<br>3: shared responsibility<br>4: full autonomy	Q14a_7_15<br>1: not allowed<br>2: under supervision<br>3: shared responsibility<br>4: full autonomy
determination of type/dosage	Q14a_4_16_1<br>Ticked = 1<br>Not ticked = 0	Q14a_2_16_1<br>Ticked = 1<br>Not ticked = 0	Q14a_3_16<br>1: under supervision<br>2: shared responsibility<br>3: full autonomy	Q39_5_16<br>1: not allowed<br>2: under supervision<br>3: shared responsibility<br>4: full autonomy	Q14a_5_16<br>1: not allowed<br>2: under supervision<br>3: shared responsibility<br>4: full autonomy	Q14a_1_16<br>1: not allowed<br>2: under supervision<br>3: shared responsibility<br>4: full autonomy	Q14a_6_16<br>1: not allowed<br>2: under supervision<br>3: shared responsibility<br>4: full autonomy	Q14a_7_16<br>1: not allowed<br>2: under supervision<br>3: shared responsibility<br>4: full autonomy
initiation of medication (reactive / proactive)	Q14a_4_17_1<br>Ticked = 1<br>Not ticked = 0	Q14a_2_17_1<br>Ticked = 1<br>Not ticked = 0	Q14a_3_17<br>1: under supervision<br>2: shared responsibility<br>3: full autonomy	Q39_5_17<br>1: not allowed<br>2: under supervision<br>3: shared responsibility<br>4: full autonomy	Q14a_5_17<br>1: not allowed<br>2: under supervision<br>3: shared responsibility<br>4: full autonomy	Q14a_1_17<br>1: not allowed<br>2: under supervision<br>3: shared responsibility<br>4: full autonomy	Q14a_6_17<br>1: not allowed<br>2: under supervision<br>3: shared responsibility<br>4: full autonomy	Q14a_7_17<br>1: not allowed<br>2: under supervision<br>3: shared responsibility<br>4: full autonomy

adaptation of dose, dose titration	**Q14a_4_18_1**<br>Ticked = 1<br>Not ticked = 0	**Q14a_2_18_1**<br>Ticked = 1<br>Not ticked = 0	**Q14a_3_18**<br>1: under supervision<br>2: shared responsibility<br>3: full autonomy	**Q39_5_18**<br>1: not allowed<br>2: under supervision<br>3: shared responsibility<br>4: full autonomy	**Q14a_5_18**<br>1: not allowed<br>2: under supervision<br>3: shared responsibility<br>4: full autonomy	**Q14a_1_18**<br>1: not allowed<br>2: under supervision<br>3: shared responsibility<br>4: full autonomy	**Q14a_6_18**<br>1: not allowed<br>2: under supervision<br>3: shared responsibility<br>4: full autonomy	**Q14a_7_18**<br>1: not allowed<br>2: under supervision<br>3: shared responsibility<br>4: full autonomy
decision on continuation /cessation of medication	**Q14a_4_19_1**<br>Ticked = 1<br>Not ticked = 0	**Q14a_2_19_1**<br>Ticked = 1<br>Not ticked = 0	**Q14a_3_19**<br>1: under supervision<br>2: shared responsibility<br>3: full autonomy	**Q39_5_19**<br>1: not allowed<br>2: under supervision<br>3: shared responsibility<br>4: full autonomy	**Q14a_5_19**<br>1: not allowed<br>2: under supervision<br>3: shared responsibility<br>4: full autonomy	**Q14a_1_19**<br>1: not allowed<br>2: under supervision<br>3: shared responsibility<br>4: full autonomy	**Q14a_6_19**<br>1: not allowed<br>2: under supervision<br>3: shared responsibility<br>4: full autonomy	**Q14a_7_19**<br>1: not allowed<br>2: under supervision<br>3: shared responsibility<br>4: full autonomy
PRN (pro re nata, 'if needed' medication) / standing prescription renewal order	**Q14a_4_20_1**<br>Ticked = 1<br>Not ticked = 0	**Q14a_2_20_1**<br>Ticked = 1<br>Not ticked = 0	**Q14a_3_20**<br>1: under supervision<br>2: shared responsibility<br>3: full autonomy	**Q39_5_20**<br>1: not allowed<br>2: under supervision<br>3: shared responsibility<br>4: full autonomy	**Q14a_5_20**<br>1: not allowed<br>2: under supervision<br>3: shared responsibility<br>4: full autonomy	**Q14a_1_20**<br>1: not allowed<br>2: under supervision<br>3: shared responsibility<br>4: full autonom	**Q14a_6_20**<br>1: not allowed<br>2: under supervision<br>3: shared responsibility<br>4: full autonomy	**Q14a_7_20**<br>1: not allowed<br>2: under supervision<br>3: shared responsibility<br>4: full autonomy
medication reconciliation	**Q14a_4_21_1**<br>Ticked = 1<br>Not ticked = 0	**Q14a_2_21_1**<br>Ticked = 1<br>Not ticked = 0	**Q14a_3_21**<br>1: under supervision<br>2: shared responsibility<br>3: full autonomy	**Q39_5_21**<br>1: not allowed<br>2: under supervision<br>3: shared responsibility<br>4: full autonomy	**Q14a_5_21**<br>1: not allowed<br>2: under supervision<br>3: shared responsibility<br>4: full autonomy	**Q14a_1_21**<br>1: not allowed<br>2: under supervision<br>3: shared responsibility<br>4: full autonom	**Q14a_6_21**<br>1: not allowed<br>2: under supervision<br>3: shared responsibility<br>4: full autonomy	**Q14a_7_21**<br>1: not allowed<br>2: under supervision<br>3: shared responsibility<br>4: full autonomy
medication review	**Q14a_4_22_1**<br>Ticked = 1<br>Not ticked = 0	**Q14a_2_22_1**<br>Ticked = 1<br>Not ticked = 0	**Q14a_3_22**<br>1: under supervision<br>2: shared responsibility<br>3: full autonomy	**Q39_5_22**<br>1: not allowed<br>2: under supervision<br>3: shared responsibility<br>4: full autonomy	**Q14a_5_22**<br>1: not allowed<br>2: under supervision<br>3: shared responsibility<br>4: full autonomy	**Q14a_1_22**<br>1: not allowed<br>2: under supervision<br>3: shared responsibility<br>4: full autonomy	**Q14a_6_22**<br>1: not allowed<br>2: under supervision<br>3: shared responsibility<br>4: full autonomy	**Q14a_7_22**<br>1: not allowed<br>2: under supervision<br>3: shared responsibility<br>4: full autonomy
intervention in case of emergency	**Q14a_4_23_1**<br>Ticked = 1<br>Not ticked = 0	**Q14a_2_23_1**<br>Ticked = 1<br>Not ticked = 0	**Q14a_3_23**<br>1: under supervision<br>2: shared responsibility<br>3: full autonomy	**Q39_5_23**<br>1: not allowed<br>2: under supervision<br>3: shared responsibility<br>4: full autonomy	**Q14a_5_23**<br>1: not allowed<br>2: under supervision<br>3: shared responsibility<br>4: full autonomy	**Q14a_1_23**<br>1: not allowed<br>2: under supervision<br>3: shared responsibility<br>4: full autonomy	**Q14a_6_23**<br>1: not allowed<br>2: under supervision<br>3: shared responsibility<br>4: full autonomy	**Q14a_7_23**<br>1: not allowed<br>2: under supervision<br>3: shared responsibility<br>4: full autonomy
transitional care communication, inter / intraprofessional collaboration / communication including reporting, advising, informing, alerting and discussing	**Q14a_4_24_1**<br>Ticked = 1<br>Not ticked = 0	**Q14a_2_24_1**<br>Ticked = 1<br>Not ticked = 0	**Q14a_3_24**<br>1: under supervision<br>2: shared responsibility<br>3: full autonomy	**Q39_5_24**<br>1: not allowed<br>2: under supervision<br>3: shared responsibility<br>4: full autonomy	**Q14a_5_24**<br>1: not allowed<br>2: under supervision<br>3: shared responsibility<br>4: full autonomy	**Q14a_1_24**<br>1: not allowed<br>2: under supervision<br>3: shared responsibility<br>4: full autonomy	**Q14a_6_24**<br>1: not allowed<br>2: under supervision<br>3: shared responsibility<br>4: full autonomy	**Q14a_7_24**<br>1: not allowed<br>2: under supervision<br>3: shared responsibility<br>4: full autonomy
collegial mentoring	**Q14a_4_25_1**<br>Ticked = 1<br>Not ticked = 0	**Q14a_2_25_1**<br>Ticked = 1<br>Not ticked = 0	**Q14a_3_25**<br>1: under supervision<br>2: shared responsibility<br>3: full autonomy	**Q39_5_25**<br>1: not allowed<br>2: under supervision<br>3: shared responsibility<br>4: full autonomy	**Q14a_5_25**<br>1: not allowed<br>2: under supervision<br>3: shared responsibility<br>4: full autonomy	**Q14a_1_25**<br>1: not allowed<br>2: under supervision<br>3: shared responsibility<br>4: full autonom	**Q14a_6_25**<br>1: not allowed<br>2: under supervision<br>3: shared responsibility<br>4: full autonomy	**Q14a_7_25**<br>1: not allowed<br>2: under supervision<br>3: shared responsibility<br>4: full autonomy
other task within prescription management:	**Q14a_4_26_1**<br>Ticked = 1<br>Not ticked = 0<br>**Q14a_4_26_TEXT**	**Q14a_2_26_1**<br>Ticked = 1<br>Not ticked = 0<br>**Q14a_2_26_TEXT**	**Q14a_3_26**<br>1: under supervision<br>2: shared responsibility<br>3: full autonomy	**Q39_5_26**<br>1: not allowed<br>2: under supervision<br>3: shared responsibility<br>4: full autonomy	**Q14a_5_26**<br>1: not allowed<br>2: under supervision<br>3: shared responsibility<br>4: full autonomy	**Q14a_1_26**<br>1: not allowed<br>2: under supervision<br>3: shared responsibility<br>4: full autonomy	**Q14a_6_26**<br>1: not allowed<br>2: under supervision<br>3: shared responsibility<br>4: full autonomy	**Q14a_7_26**<br>1: not allowed<br>2: under supervision<br>3: shared responsibility<br>4: full autonomy

			Q14a_3_26_TEXT	Q39_5_26_TEXT	Q14a_5_26_TEXT	Q14a_1_26_TEXT	Q14a_6_26_TEXT	Q14a_7_26_TEXT

Q15/ In order to obtain best quality of care and patient outcomes (= ideal situation, which can be different from the current situation)**, to what extent should nurses be allowed to prescribe medicines?**

- No nurse prescribing *(info: next question will not be shown)* **1**
- Dependent prescribing **2**
- Independent prescribing **3**

Q16/ In an ideal interprofessional healthcare situation, which restrictions would be needed to optimise nurse prescribing? (tick all that apply)

- **Q16_1** no restrictions **= 1; restrictions = 0**
- **Q16_2** only a restricted list of medicines **=1; this restriction is not applicable =0**
- **Q16_3** only in a specific context, pathology / specialisation **=1; this restriction is not applicable =0**
- **Q16_4** only after specific training **=1; this restriction is not applicable =0**
- **Q16_6** only long-term chronic medicines **=1; this restriction is not applicable =0**
- **Q16_7** only low risk medicines such as? **=1; this restriction is not applicable =0**
- **Q16_8** prescription-only medicines only **=1; this restriction is not applicable =0**
- **Q16_9** only in emergency**=1; this restriction is not applicable =0**
- **Q16_10** only within an individual patient clinical management plan **=1; this restriction is not applicable =0**
- **Q16_5** other: **=1; no other restrictions applicable =0** … **Q16_5_TEXT**

Q17a/ RESPONSIBILITY 6: medication safety management.

	**Task not relevant** within medication safety management. (skip following columns, when	**Task not allowed** for nurses within medication safety management. (skip following columns, when	Task allowed for **all levels of** nurses within medication safety management. Specify the level	Specify the level of responsibility for **level 4** nurses performing this task within medication	Specify the level of responsibility for **level 5** nurses performing this task within medication	Specify the level of responsibility for **level 6** nurses performing this task within medication	Specify the level of responsibility for **level 7** nurses performing this task within medication	Specify the level of responsibility for **level 8** nurses performing this task within medication

	this options is ticked)	this options is ticked)	of responsibility. (skip following columns, when this options is ticked)	safety management Variable only applicable in Germany and the Netherlands	safety management	safety management	safety management	safety management
observation, documentation, registration, report	Q17a_4_1_1<br>Ticked = 1<br>Not ticked = 0	Q17a_2_1_1<br>Ticked = 1<br>Not ticked = 0	Q17a_3_1<br>1: under supervision<br>2: shared responsibility<br>3: full autonomy	Q38_5_1<br>1: not allowed<br>2: under supervision<br>3: shared responsibility<br>4: full autonomy	Q17a_5_1<br>1: not allowed<br>2: under supervision<br>3: shared responsibility<br>4: full autonomy	Q17a_1_1<br>1: not allowed<br>2: under supervision<br>3: shared responsibility<br>4: full autonomy	Q17a_6_1<br>1: not allowed<br>2: under supervision<br>3: shared responsibility<br>4: full autonomy	Q17a_7_1<br>1: not allowed<br>2: under supervision<br>3: shared responsibility<br>4: full autonomy
assessing patients competences	Q17a_4_2_1Tic<br>ked = 1<br>Not ticked = 0	Q17a_2_2_1<br>Ticked = 1<br>Not ticked = 0	Q17a_3_2<br>1: under supervision<br>2: shared responsibility<br>3: full autonomy	Q38_5_2<br>1: not allowed<br>2: under supervision<br>3: shared responsibility<br>4: full autonomy	Q17a_5_2<br>1: not allowed<br>2: under supervision<br>3: shared responsibility<br>4: full autonomy	Q17a_1_2<br>1: not allowed<br>2: under supervision<br>3: shared responsibility<br>4: full autonomy	Q17a_6_2<br>1: not allowed<br>2: under supervision<br>3: shared responsibility<br>4: full autonomy	Q17a_7_2<br>1: not allowed<br>2: under supervision<br>3: shared responsibility<br>4: full autonomy
assessment and addressing patient/family needs	Q17a_4_3_1<br>Ticked = 1<br>Not ticked = 0	Q17a_2_3_1<br>Ticked = 1<br>Not ticked = 0	Q17a_3_3<br>1: under supervision<br>2: shared responsibility<br>3: full autonomy	Q38_5_3<br>1: not allowed<br>2: under supervision<br>3: shared responsibility<br>4: full autonomy	Q17a_5_3<br>1: not allowed<br>2: under supervision<br>3: shared responsibility<br>4: full autonomy	Q17a_1_3<br>1: not allowed<br>2: under supervision<br>3: shared responsibility<br>4: full autonomy	Q17a_6_3<br>1: not allowed<br>2: under supervision<br>3: shared responsibility<br>4: full autonomy	Q17a_7_3<br>1: not allowed<br>2: under supervision<br>3: shared responsibility<br>4: full autonomy
recognising & preventing risks / complications / medication errors	Q17a_4_4_1<br>Ticked = 1<br>Not ticked = 0	Q17a_2_4_1<br>Ticked = 1<br>Not ticked = 0	Q17a_3_4<br>1: under supervision<br>2: shared responsibility<br>3: full autonomy	Q38_5_4<br>1: not allowed<br>2: under supervision<br>3: shared responsibility<br>4: full autonomy	Q17a_5_4<br>1: not allowed<br>2: under supervision<br>3: shared responsibility<br>4: full autonomy	Q17a_1_4<br>1: not allowed<br>2: under supervision<br>3: shared responsibility<br>4: full autonomy	Q17a_6_4<br>1: not allowed<br>2: under supervision<br>3: shared responsibility<br>4: full autonomy	Q17a_7_4<br>1: not allowed<br>2: under supervision<br>3: shared responsibility<br>4: full autonomy
identification, reporting and addressing contra-indications, drug related problems	Q17a_4_5_1<br>Ticked = 1<br>Not ticked = 0	Q17a_2_5_1<br>Ticked = 1<br>Not ticked = 0	Q17a_3_5<br>1: under supervision<br>2: shared responsibility<br>3: full autonomy	Q38_5_5<br>1: not allowed<br>2: under supervision<br>3: shared responsibility<br>4: full autonomy	Q17a_5_5<br>1: not allowed<br>2: under supervision<br>3: shared responsibility<br>4: full autonomy	Q17a_1_5<br>1: not allowed<br>2: under supervision<br>3: shared responsibility<br>4: full autonomy	Q17a_6_5<br>1: not allowed<br>2: under supervision<br>3: shared responsibility<br>4: full autonomy	Q17a_7_5<br>1: not allowed<br>2: under supervision<br>3: shared responsibility<br>4: full autonomy
follow-up	Q17a_4_6_1<br>Ticked = 1<br>Not ticked = 0	Q17a_2_6_1<br>Ticked = 1<br>Not ticked = 0	Q17a_3_6<br>1: under supervision<br>2: shared responsibility<br>3: full autonomy	Q38_5_6<br>1: not allowed<br>2: under supervision<br>3: shared responsibility<br>4: full autonomy	Q17a_5_6<br>1: not allowed<br>2: under supervision<br>3: shared responsibility<br>4: full autonomy	Q17a_1_6<br>1: not allowed<br>2: under supervision<br>3: shared responsibility<br>4: full autonomy	Q17a_6_6<br>1: not allowed<br>2: under supervision<br>3: shared responsibility<br>4: full autonomy	Q17a_7_6<br>1: not allowed<br>2: under supervision<br>3: shared responsibility<br>4: full autonomy
evidence based practice	Q17a_4_7_1<br>Ticked = 1<br>Not ticked = 0	Q17a_2_7_1<br>Ticked = 1<br>Not ticked = 0	Q17a_3_7<br>1: under supervision<br>2: shared responsibility<br>3: full autonomy	Q38_5_7<br>1: not allowed<br>2: under supervision<br>3: shared responsibility<br>4: full autonomy	Q17a_5_7<br>1: not allowed<br>2: under supervision<br>3: shared responsibility<br>4: full autonomy	Q17a_1_7<br>1: not allowed<br>2: under supervision<br>3: shared responsibility<br>4: full autonomy	Q17a_6_7<br>1: not allowed<br>2: under supervision<br>3: shared responsibility<br>4: full autonomy	Q17a_7_7<br>1: not allowed<br>2: under supervision<br>3: shared responsibility<br>4: full autonomy
decision making	Q17a_4_8_1<br>Ticked = 1<br>Not ticked = 0	Q17a_2_8_1<br>Ticked = 1<br>Not ticked = 0	Q17a_3_8<br>1: under supervision<br>2: shared responsibility<br>3: full autonomy	Q38_5_8<br>1: not allowed<br>2: under supervision<br>3: shared responsibility<br>4: full autonomy	Q17a_5_8<br>1: not allowed<br>2: under supervision<br>3: shared responsibility<br>4: full autonomy	Q17a_1_8<br>1: not allowed<br>2: under supervision<br>3: shared responsibility<br>4: full autonomy	Q17a_6_8<br>1: not allowed<br>2: under supervision<br>3: shared responsibility<br>4: full autonomy	Q17a_7_8<br>1: not allowed<br>2: under supervision<br>3: shared responsibility<br>4: full autonomy
communication /discussion with patient/family	Q17a_4_9_1<br>Ticked = 1<br>Not ticked = 0	Q17a_2_9_1<br>Ticked = 1<br>Not ticked = 0	Q17a_3_9<br>1: under supervision<br>2: shared responsibility<br>3: full autonomy	Q38_5_9<br>1: not allowed<br>2: under supervision<br>3: shared responsibility	Q17a_5_9<br>1: not allowed<br>2: under supervision<br>3: shared responsibility	Q17a_1_9<br>1: not allowed<br>2: under supervision<br>3: shared responsibility	Q17a_6_9<br>1: not allowed<br>2: under supervision<br>3: shared responsibility	Q17a_7_9<br>1: not allowed<br>2: under supervision<br>3: shared responsibility

				4: full autonomy	4: full autonomy	4: full autonomy	4: full autonomy	4: full autonomy	
Inter / intraprofessional referrals	**Q17a_4_10_1**<br>Ticked = 1<br>Not ticked = 0	**Q17a_2_10_1**<br>Ticked = 1<br>Not ticked = 0	**Q17a_3_10**<br>1: under supervision<br>2: shared responsibility<br>3: full autonomy	**Q38_5_10**<br>1: not allowed<br>2: under supervision<br>3: shared responsibility<br>4: full autonomy	**Q17a_5_10**<br>1: not allowed<br>2: under supervision<br>3: shared responsibility<br>4: full autonomy	**Q17a_1_10**<br>1: not allowed<br>2: under supervision<br>3: shared responsibility<br>4: full autonomy	**Q17a_6_10**<br>1: not allowed<br>2: under supervision<br>3: shared responsibility<br>4: full autonomy	**Q17a_7_10**<br>1: not allowed<br>2: under supervision<br>3: shared responsibility<br>4: full autonomy	
facilitation of medication management	**Q17a_4_12_1**<br>Ticked = 1<br>Not ticked = 0	**Q17a_2_12_1**<br>Ticked = 1<br>Not ticked = 0	**Q17a_3_12**<br>1: under supervision<br>2: shared responsibility<br>3: full autonomy	**Q38_5_12**<br>1: not allowed<br>2: under supervision<br>3: shared responsibility<br>4: full autonomy	**Q17a_5_12**<br>1: not allowed<br>2: under supervision<br>3: shared responsibility<br>4: full autonomy	**Q17a_1_12**<br>1: not allowed<br>2: under supervision<br>3: shared responsibility<br>4: full autonomy	**Q17a_6_12**<br>1: not allowed<br>2: under supervision<br>3: shared responsibility<br>4: full autonomy	**Q17a_7_12**<br>1: not allowed<br>2: under supervision<br>3: shared responsibility<br>4: full autonomy	
selfcare support and therapeutic education	**Q17a_4_15_1**<br>Ticked = 1<br>Not ticked = 0	**Q17a_2_15_1**<br>Ticked = 1<br>Not ticked = 0	**Q17a_3_15**<br>1: under supervision<br>2: shared responsibility<br>3: full autonomy	**Q38_5_15**<br>1: not allowed<br>2: under supervision<br>3: shared responsibility<br>4: full autonomy	**Q17a_5_15**<br>1: not allowed<br>2: under supervision<br>3: shared responsibility<br>4: full autonomy	**Q17a_1_15**<br>1: not allowed<br>2: under supervision<br>3: shared responsibility<br>4: full autonomy	**Q17a_6_15**<br>1: not allowed<br>2: under supervision<br>3: shared responsibility<br>4: full autonomy	**Q17a_7_15**<br>1: not allowed<br>2: under supervision<br>3: shared responsibility<br>4: full autonomy	
advice (patient or other healthcare professional)	**Q17a_4_16_1**<br>Ticked = 1<br>Not ticked = 0	**Q17a_2_16_1**<br>Ticked = 1<br>Not ticked = 0	**Q17a_3_16**<br>1: under supervision<br>2: shared responsibility<br>3: full autonomy	**Q38_5_16**<br>1: not allowed<br>2: under supervision<br>3: shared responsibility<br>4: full autonomy	**Q17a_5_16**<br>1: not allowed<br>2: under supervision<br>3: shared responsibility<br>4: full autonomy	**Q17a_1_16**<br>1: not allowed<br>2: under supervision<br>3: shared responsibility<br>4: full autonomy	**Q17a_6_16**<br>1: not allowed<br>2: under supervision<br>3: shared responsibility<br>4: full autonomy	**Q17a_7_16**<br>1: not allowed<br>2: under supervision<br>3: shared responsibility<br>4: full autonomy	
intervention in case of emergency	**Q17a_4_17_1**<br>Ticked = 1<br>Not ticked = 0	**Q17a_2_17_1**<br>Ticked = 1<br>Not ticked = 0	**Q17a_3_17**<br>1: under supervision<br>2: shared responsibility<br>3: full autonomy	**Q38_5_17**<br>1: not allowed<br>2: under supervision<br>3: shared responsibility<br>4: full autonomy	**Q17a_5_17**<br>1: not allowed<br>2: under supervision<br>3: shared responsibility<br>4: full autonomy	**Q17a_1_17**<br>1: not allowed<br>2: under supervision<br>3: shared responsibility<br>4: full autonomy	**Q17a_6_17**<br>1: not allowed<br>2: under supervision<br>3: shared responsibility<br>4: full autonomy	**Q1a_7_17**<br>1: not allowed<br>2: under supervision<br>3: shared responsibility<br>4: full autonomy	
transitional care communication, inter / intraprofessional collaboration / communication including reporting, advising, informing, alerting and discussing	**Q17a_4_18_1**<br>Ticked = 1<br>Not ticked = 0	**Q17a_2_18_1**<br>Ticked = 1<br>Not ticked = 0	**Q17a_3_18**<br>1: under supervision<br>2: shared responsibility<br>3: full autonomy	**Q38_5_18**<br>1: not allowed<br>2: under supervision<br>3: shared responsibility<br>4: full autonomy	**Q17a_5_18**<br>1: not allowed<br>2: under supervision<br>3: shared responsibility<br>4: full autonomy	**Q17a_1_18**<br>1: not allowed<br>2: under supervision<br>3: shared responsibility<br>4: full autonomy	**Q17a_6_18**<br>1: not allowed<br>2: under supervision<br>3: shared responsibility<br>4: full autonomy	**Q17a_7_18**<br>1: not allowed<br>2: under supervision<br>3: shared responsibility<br>4: full autonomy	
collegial mentoring	**Q17a_4_19_1**<br>Ticked = 1<br>Not ticked = 0	**Q17a_2_19_1**<br>Ticked = 1<br>Not ticked = 0	**Q17a_3_19**<br>1: under supervision<br>2: shared responsibility<br>3: full autonomy	**Q38_5_19**<br>1: not allowed<br>2: under supervision<br>3: shared responsibility<br>4: full autonomy	**Q17a_5_19**<br>1: not allowed<br>2: under supervision<br>3: shared responsibility<br>4: full autonomy	**Q17a_1_19**<br>1: not allowed<br>2: under supervision<br>3: shared responsibility<br>4: full autonomy	**Q17a_6_19**<br>1: not allowed<br>2: under supervision<br>3: shared responsibility<br>4: full autonomy	**Q17a_7_19**<br>1: not allowed<br>2: under supervision<br>3: shared responsibility<br>4: full autonomy	
other task within medication safety management:	**Q17a_4_20_1**<br>Ticked = 1<br>Not ticked = 0<br>**Q17a_4_20_TEXT**	**Q17a_2_20_1**<br>Ticked = 1<br>Not ticked = 0<br>**Q17a_2_20_TEXT**	**Q17a_3_20**<br>1: under supervision<br>2: shared responsibility<br>3: full autonomy<br>**Q17a_3_20_TEXT**	**Q38_5_20**<br>1: not allowed<br>2: under supervision<br>3: shared responsibility<br>4: full autonomy<br>**Q38_5_20_TEXT**	**Q17a_5_20**<br>1: not allowed<br>2: under supervision<br>3: shared responsibility<br>4: full autonomy<br>**Q17a_5_20_TEXT**	**Q17a_1_20**<br>1: not allowed<br>2: under supervision<br>3: shared responsibility<br>4: full autonom<br>**Q17a_1_20_TEXT**	**Q17a_6_20**<br>1: not allowed<br>2: under supervision<br>3: shared responsibility<br>4: full autonomy<br>**Q17a_6_20_TEXT**	**Q17a_7_20**<br>1: not allowed<br>2: under supervision<br>3: shared responsibility<br>4: full autonomy<br>**Q17a_7_20_TEXT**	

Q18a/ RESPONSIBILITY 7: (transition of) care co-ordination.

	**Task not relevant** within (transition of) care coordination. (skip following columns, when this options is ticked)	**Task not allowed** for nurses within (transition of) care coordination. (skip following columns, when this options is ticked)	Task allowed for **all levels** of nurses within (transition of) care coordination. Specify the level of responsibility. (skip following columns, when this options is ticked)	Specify the level of responsibility for **level 4** nurses performing this task within (transition of) care coordination Variable only applicable in Germany and the Netherlands	Specify the level of responsibility for **level 5** nurses performing this task within (transition of) care coordination	Specify the level of responsibility for **level 6** nurses performing this task within (transition of) care coordination	Specify the level of responsibility for **level 7** nurses performing this task within (transition of) care coordination	Specify the level of responsibility for **level 8** nurses performing this task within (transition of) care coordination
observation, documentation, registration, report	**Q18a_4_1_1**<br>Ticked = 1<br>Not ticked = 0	**Q18a_2_1_1**<br>Ticked = 1<br>Not ticked = 0	**Q18a_3_1**<br>1: under supervision<br>2: shared responsibility<br>3: full autonomy	**Q37_5_1**<br>1: not allowed<br>2: under supervision<br>3: shared responsibility<br>4: full autonomy	**Q18a_5_1**<br>1: not allowed<br>2: under supervision<br>3: shared responsibility<br>4: full autonomy	**Q18a_1_1**<br>1: not allowed<br>2: under supervision<br>3: shared responsibility<br>4: full autonomy	**Q12a_6_1**<br>1: not allowed<br>2: under supervision<br>3: shared responsibility<br>4: full autonomy	**Q12a_7_1**<br>1: not allowed<br>2: under supervision<br>3: shared responsibility<br>4: full autonomy
assessing patients competences	**Q18a_4_2_1**<br>Ticked = 1<br>Not ticked = 0	**Q18a_2_2_1**<br>Ticked = 1<br>Not ticked = 0	**Q18a_3_2**<br>1: under supervision<br>2: shared responsibility<br>3: full autonomy	**Q37_5_2**<br>1: not allowed<br>2: under supervision<br>3: shared responsibility<br>4: full autonomy	**Q18a_5_2**<br>1: not allowed<br>2: under supervision<br>3: shared responsibility<br>4: full autonomy	**Q18a_1_2**<br>1: not allowed<br>2: under supervision<br>3: shared responsibility<br>4: full autonomy	**Q12a_6_2**<br>1: not allowed<br>2: under supervision<br>3: shared responsibility<br>4: full autonomy	**Q12a_7_2**<br>1: not allowed<br>2: under supervision<br>3: shared responsibility<br>4: full autonomy
assessment and addressing patient/family needs	**Q18a_4_3_1**<br>Ticked = 1<br>Not ticked = 0	**Q18a_2_3_1**<br>Ticked = 1<br>Not ticked = 0	**Q18a_3_3**<br>1: under supervision<br>2: shared responsibility<br>3: full autonomy	**Q37_5_3**<br>1: not allowed<br>2: under supervision<br>3: shared responsibility<br>4: full autonomy	**Q18a_5_3**<br>1: not allowed<br>2: under supervision<br>3: shared responsibility<br>4: full autonomy	**Q18a_1_3**<br>1: not allowed<br>2: under supervision<br>3: shared responsibility<br>4: full autonomy	**Q12a_6_3**<br>1: not allowed<br>2: under supervision<br>3: shared responsibility<br>4: full autonomy	**Q12a_7_3**<br>1: not allowed<br>2: under supervision<br>3: shared responsibility<br>4: full autonomy
recognising & preventing risks / complications / medication errors	**Q18a_4_4_1**<br>Ticked = 1<br>Not ticked = 0	**Q18a_2_4_1**<br>Ticked = 1<br>Not ticked = 0	**Q18a_3_4**<br>1: under supervision<br>2: shared responsibility<br>3: full autonomy	**Q37_5_4**<br>1: not allowed<br>2: under supervision<br>3: shared responsibility<br>4: full autonomy	**Q18a_5_4**<br>1: not allowed<br>2: under supervision<br>3: shared responsibility<br>4: full autonomy	**Q18a_1_4**<br>1: not allowed<br>2: under supervision<br>3: shared responsibility<br>4: full autonomy	**Q12a_6_4**<br>1: not allowed<br>2: under supervision<br>3: shared responsibility<br>4: full autonomy	**Q12a_7_4**<br>1: not allowed<br>2: under supervision<br>3: shared responsibility<br>4: full autonomy
identification, reporting and addressing contra-indications, drug related problems	**Q18a_4_5_1**<br>Ticked = 1<br>Not ticked = 0	**Q18a_2_5_1**<br>Ticked = 1<br>Not ticked = 0	**Q18a_3_5**<br>1: under supervision<br>2: shared responsibility<br>3: full autonomy	**Q37_5_5**<br>1: not allowed<br>2: under supervision<br>3: shared responsibility<br>4: full autonomy	**Q18a_5_5**<br>1: not allowed<br>2: under supervision<br>3: shared responsibility<br>4: full autonomy	**Q18a_1_5**<br>1: not allowed<br>2: under supervision<br>3: shared responsibility<br>4: full autonomy	**Q12a_6_5**<br>1: not allowed<br>2: under supervision<br>3: shared responsibility<br>4: full autonomy	**Q12a_7_5**<br>1: not allowed<br>2: under supervision<br>3: shared responsibility<br>4: full autonomy
follow-up	**Q18a_4_6_1**<br>Ticked = 1<br>Not ticked = 0	**Q18a_2_6_1**<br>Ticked = 1<br>Not ticked = 0	**Q18a_3_6**<br>1: under supervision<br>2: shared responsibility<br>3: full autonomy	**Q37_5_6**<br>1: not allowed<br>2: under supervision<br>3: shared responsibility<br>4: full autonomy	**Q18a_5_6**<br>1: not allowed<br>2: under supervision<br>3: shared responsibility<br>4: full autonomy	**Q18a_1_6**<br>1: not allowed<br>2: under supervision<br>3: shared responsibility<br>4: full autonomy	**Q12a_6_6**<br>1: not allowed<br>2: under supervision<br>3: shared responsibility<br>4: full autonomy	**Q12a_7_6**<br>1: not allowed<br>2: under supervision<br>3: shared responsibility<br>4: full autonomy
evidence based practice	**Q18a_4_7_1**<br>Ticked = 1<br>Not ticked = 0	**Q18a_2_7_1**<br>Ticked = 1<br>Not ticked = 0	**Q18a_3_7**<br>1: under supervision<br>2: shared responsibility<br>3: full autonomy	**Q37_5_7**<br>1: not allowed<br>2: under supervision<br>3: shared responsibility<br>4: full autonomy	**Q18a_5_7**<br>1: not allowed<br>2: under supervision<br>3: shared responsibility<br>4: full autonomy	**Q18a_1_7**<br>1: not allowed<br>2: under supervision<br>3: shared responsibility<br>4: full autonomy	**Q12a_6_7**<br>1: not allowed<br>2: under supervision<br>3: shared responsibility<br>4: full autonomy	**Q12a_7_7**<br>1: not allowed<br>2: under supervision<br>3: shared responsibility<br>4: full autonomy

decision making	Q18a_4_8_1<br>Ticked = 1<br>Not ticked = 0	Q18a_2_8_1<br>Ticked = 1<br>Not ticked = 0	Q18a_3_8<br>1: under supervision<br>2: shared responsibility<br>3: full autonomy	Q37_5_8<br>1: not allowed<br>2: under supervision<br>3: shared responsibility<br>4: full autonomy	Q18a_5_8<br>1: not allowed<br>2: under supervision<br>3: shared responsibility<br>4: full autonomy	Q18a_1_8<br>1: not allowed<br>2: under supervision<br>3: shared responsibility<br>4: full autonomy	Q12a_6_8<br>1: not allowed<br>2: under supervision<br>3: shared responsibility<br>4: full autonomy	Q12a_7_8<br>1: not allowed<br>2: under supervision<br>3: shared responsibility<br>4: full autonomy
communication /discussion with patient/family	Q18a_4_9_1<br>Ticked = 1<br>Not ticked = 0	Q18a_2_9_1<br>Ticked = 1<br>Not ticked = 0	Q18a_3_9<br>1: under supervision<br>2: shared responsibility<br>3: full autonomy	Q37_5_9<br>1: not allowed<br>2: under supervision<br>3: shared responsibility<br>4: full autonomy	Q18a_5_9<br>1: not allowed<br>2: under supervision<br>3: shared responsibility<br>4: full autonomy	Q18a_1_9<br>1: not allowed<br>2: under supervision<br>3: shared responsibility<br>4: full autonomy	Q12a_6_9<br>1: not allowed<br>2: under supervision<br>3: shared responsibility<br>4: full autonomy	Q12a_7_9<br>1: not allowed<br>2: under supervision<br>3: shared responsibility<br>4: full autonomy
Inter / intraprofessional referrals	Q18a_4_10_1<br>Ticked = 1<br>Not ticked = 0	Q18a_2_10_1<br>Ticked = 1<br>Not ticked = 0	Q18a_3_10<br>1: under supervision<br>2: shared responsibility<br>3: full autonomy	Q37_5_10<br>1: not allowed<br>2: under supervision<br>3: shared responsibility<br>4: full autonomy	Q18a_5_10<br>1: not allowed<br>2: under supervision<br>3: shared responsibility<br>4: full autonomy	Q18a_1_10<br>1: not allowed<br>2: under supervision<br>3: shared responsibility<br>4: full autonomy	Q12a_6_10<br>1: not allowed<br>2: under supervision<br>3: shared responsibility<br>4: full autonomy	Q12a_7_10<br>1: not allowed<br>2: under supervision<br>3: shared responsibility<br>4: full autonomy
facilitation of medication management	Q18a_4_12_1<br>Ticked = 1<br>Not ticked = 0	Q18a_2_12_1<br>Ticked = 1<br>Not ticked = 0	Q18a_3_12<br>1: under supervision<br>2: shared responsibility<br>3: full autonomy	Q37_5_12<br>1: not allowed<br>2: under supervision<br>3: shared responsibility<br>4: full autonomy	Q18a_5_12<br>1: not allowed<br>2: under supervision<br>3: shared responsibility<br>4: full autonomy	Q18a_1_12<br>1: not allowed<br>2: under supervision<br>3: shared responsibility<br>4: full autonomy	Q12a_6_12<br>1: not allowed<br>2: under supervision<br>3: shared responsibility<br>4: full autonomy	Q12a_7_12<br>1: not allowed<br>2: under supervision<br>3: shared responsibility<br>4: full autonomy
selfcare support and therapeutic education	Q18a_4_15_1<br>Ticked = 1<br>Not ticked = 0	Q18a_2_15_1<br>Ticked = 1<br>Not ticked = 0	Q18a_3_15<br>1: under supervision<br>2: shared responsibility<br>3: full autonomy	Q37_5_15<br>1: not allowed<br>2: under supervision<br>3: shared responsibility<br>4: full autonomy	Q18a_5_15<br>1: not allowed<br>2: under supervision<br>3: shared responsibility<br>4: full autonomy	Q18a_1_15<br>1: not allowed<br>2: under supervision<br>3: shared responsibility<br>4: full autonomy	Q12a_6_15<br>1: not allowed<br>2: under supervision<br>3: shared responsibility<br>4: full autonomy	Q12a_7_15<br>1: not allowed<br>2: under supervision<br>3: shared responsibility<br>4: full autonomy
advice (patient or other healthcare professional)	Q18a_4_16_1<br>Ticked = 1<br>Not ticked = 0	Q18a_2_16_1<br>Ticked = 1<br>Not ticked = 0	Q18a_3_16<br>1: under supervision<br>2: shared responsibility<br>3: full autonomy	Q37_5_16<br>1: not allowed<br>2: under supervision<br>3: shared responsibility<br>4: full autonomy	Q18a_5_16<br>1: not allowed<br>2: under supervision<br>3: shared responsibility<br>4: full autonomy	Q18a_1_16<br>1: not allowed<br>2: under supervision<br>3: shared responsibility<br>4: full autonomy	Q12a_6_16<br>1: not allowed<br>2: under supervision<br>3: shared responsibility<br>4: full autonomy	Q12a_7_16<br>1: not allowed<br>2: under supervision<br>3: shared responsibility<br>4: full autonomy
discharge planning, transition of care planning	Q18a_4_17_1<br>Ticked = 1<br>Not ticked = 0	Q18a_2_17_1<br>Ticked = 1<br>Not ticked = 0	Q18a_3_17<br>1: under supervision<br>2: shared responsibility<br>3: full autonomy	Q37_5_17<br>1: not allowed<br>2: under supervision<br>3: shared responsibility<br>4: full autonomy	Q18a_5_17<br>1: not allowed<br>2: under supervision<br>3: shared responsibility<br>4: full autonomy	Q18a_1_17<br>1: not allowed<br>2: under supervision<br>3: shared responsibility<br>4: full autonomy	Q12a_6_17<br>1: not allowed<br>2: under supervision<br>3: shared responsibility<br>4: full autonomy	Q12a_7_17<br>1: not allowed<br>2: under supervision<br>3: shared responsibility<br>4: full autonomy
transitional care communication, inter / intraprofessional collaboration / communication including reporting, advising, informing, alerting and discussing	Q18a_4_18_1<br>Ticked = 1<br>Not ticked = 0	Q18a_2_18_1<br>Ticked = 1<br>Not ticked = 0	Q18a_3_18<br>1: under supervision<br>2: shared responsibility<br>3: full autonomy	Q37_5_18<br>1: not allowed<br>2: under supervision<br>3: shared responsibility<br>4: full autonomy	Q18a_5_18<br>1: not allowed<br>2: under supervision<br>3: shared responsibility<br>4: full autonomy	Q18a_1_18<br>1: not allowed<br>2: under supervision<br>3: shared responsibility<br>4: full autonomy	Q18a_6_18<br>1: not allowed<br>2: under supervision<br>3: shared responsibility<br>4: full autonomy	Q18a_7_18<br>1: not allowed<br>2: under supervision<br>3: shared responsibility<br>4: full autonomy
collegial mentoring	Q18a_4_19_1<br>Ticked = 1<br>Not ticked = 0	Q18a_2_19_1<br>Ticked = 1<br>Not ticked = 0	Q18a_3_19<br>1: under supervision<br>2: shared responsibility<br>3: full autonomy	Q37_5_19<br>1: not allowed<br>2: under supervision<br>3: shared responsibility<br>4: full autonomy	Q18a_5_19<br>1: not allowed<br>2: under supervision<br>3: shared responsibility<br>4: full autonomy	Q18a_1_19<br>1: not allowed<br>2: under supervision<br>3: shared responsibility<br>4: full autonomy	Q18a_6_19<br>1: not allowed<br>2: under supervision<br>3: shared responsibility<br>4: full autonomy	Q18a_7_19<br>1: not allowed<br>2: under supervision<br>3: shared responsibility<br>4: full autonomy

other task within (transition of) care coordination:	Q18a_4_20_1<br>Ticked = 1<br>Not ticked = 0<br>Q18a_4_20_TEXT	Q18a_2_20_1<br>Ticked = 1<br>Not ticked = 0<br>Q18a_2_20_TEXT	Q18a_3_20<br>1: under supervision<br>2: shared responsibility<br>3: full autonomy<br>Q18a_3_20_TEXT	Q37_5_20<br>1: not allowed<br>2: under supervision<br>3: shared responsibility<br>4: full autonomy<br>Q37_5_20_TEXT	Q18a_5_20<br>1: not allowed<br>2: under supervision<br>3: shared responsibility<br>4: full autonomy<br>Q18a_5_20_TEXT	Q1a_1_20<br>1: not allowed<br>2: under supervision<br>3: shared responsibility<br>4: full autonom<br>Q18a_1_20_TEXT	Q18a_6_20<br>1: not allowed<br>2: under supervision<br>3: shared responsibility<br>4: full autonomy<br>Q18a_6_20_TEXT	Q18a_7_20<br>1: not allowed<br>2: under supervision<br>3: shared responsibility<br>4: full autonomy<br>Q18a_7_20_TEXT

Q10b/Nurses can have different responsibilities within medicines management. We defined several tasks within 7 responsibilities beyond preparation and administration of medicines.

We want you to think about the ideal situation to obtain best quality of interprofessional care and patient outcomes. You do not have to indicate the current nursing situation.

Please indicate for each task, whether you think this could be a nursing task.

RESPONSIBILITY 1: management of therapeutic and adverse effects of medicines.

	**Task not relevant** within management of therapeutic and adverse effects of medicines.	**Task PROHIBITED** for nurses within management of therapeutic and adverse effects of medicines.	**Task PERMITTED** for nurses within management of therapeutic and adverse effects of medicines. Specify the level of responsibility.
observation, documentation, registration, reporting	Q10b_3_1_1<br>Ticked = 1<br>Not ticked = 0	Q10b_1_1_1<br>Ticked = 1<br>Not ticked = 0	Q10b_2_1<br>1: under supervision<br>2: shared responsibility<br>3: full autonomy
assessing patients' competences	Q10b_3_2_1<br>Ticked = 1<br>Not ticked = 0	Q10b_1_2_1<br>Ticked = 1<br>Not ticked = 0	Q10b_2_2<br>1: under supervision<br>2: shared responsibility<br>3: full autonomy
assessment and addressing patient/family needs	Q10b_3_3_1<br>Ticked = 1<br>Not ticked = 0	Q10b_1_3_1<br>Ticked = 1<br>Not ticked = 0	Q10b_2_3<br>1: under supervision<br>2: shared responsibility<br>3: full autonomy
recognising & preventing risks / complications / medication errors	Q10b_3_4_1<br>Ticked = 1<br>Not ticked = 0	Q10b_1_4_1<br>Ticked = 1<br>Not ticked = 0	Q10b_2_4<br>1: under supervision<br>2: shared responsibility<br>3: full autonomy
identification, reporting and addressing contra-indications to administration, identification of drug related problems	Q10b_3_5_1<br>Ticked = 1<br>Not ticked = 0	Q10b_1_5_1<br>Ticked = 1<br>Not ticked = 0	Q10b_2_5<br>1: under supervision<br>2: shared responsibility<br>3: full autonomy
follow-up	Q10b_3_6_1	Q10b_1_6_1	Q10b_2_6

	Ticked = 1<br>Not ticked = 0	Ticked = 1<br>Not ticked = 0	1: under supervision<br>2: shared responsibility<br>3: full autonomy
Adhering to evidence based practice as described in clinical guidelines	Q10b_3_7_1<br>Ticked = 1<br>Not ticked = 0	Q10b_1_7_1<br>Ticked = 1<br>Not ticked = 0	Q10b_2_7<br>1: under supervision<br>2: shared responsibility<br>3: full autonomy
decision making	Q10b_3_8_1<br>Ticked = 1<br>Not ticked = 0	Q10b_1_8_1<br>Ticked = 1<br>Not ticked = 0	Q10b_2_8<br>1: under supervision<br>2: shared responsibility<br>3: full autonomy
communication /discussion with patient/family	Q10b_3_9_1<br>Ticked = 1<br>Not ticked = 0	Q10b_1_9_1<br>Ticked = 1<br>Not ticked = 0	Q10b_2_9<br>1: under supervision<br>2: shared responsibility<br>3: full autonomy
Inter / intraprofessional referrals	Q10b_3_10_1<br>Ticked = 1<br>Not ticked = 0	Q10b_10_1<br>Ticked = 1<br>Not ticked = 0	Q10b_2_10<br>1: under supervision<br>2: shared responsibility<br>3: full autonomy
facilitation of medication management	Q10b_3_12_1<br>Ticked = 1<br>Not ticked = 0	Q10b_1_12_1<br>Ticked = 1<br>Not ticked = 0	Q10b_2_12<br>1: under supervision<br>2: shared responsibility<br>3: full autonomy
self-care support and education of patients	Q10b_3_15_1<br>Ticked = 1<br>Not ticked = 0	Q10b_1_15_1<br>Ticked = 1<br>Not ticked = 0	Q10b_2_15<br>1: under supervision<br>2: shared responsibility<br>3: full autonomy
advice (to patient or other healthcare professional)	Q10b_3_16_1<br>Ticked = 1<br>Not ticked = 0	Q10b_1_16_1<br>Ticked = 1<br>Not ticked = 0	Q10b_2_16<br>1: under supervision<br>2: shared responsibility<br>3: full autonomy
transitional care communication, inter / intraprofessional collaboration / communication including reporting, advising, informing, alerting and discussing	Q10b_3_17_1<br>Ticked = 1<br>Not ticked = 0	Q10b_1_17_1<br>Ticked = 1<br>Not ticked = 0	Q10b_2_17<br>1: under supervision<br>2: shared responsibility<br>3: full autonomy
mentoring colleagues	Q10b_3_18_1<br>Ticked = 1<br>Not ticked = 0	Q10b_1_18_1<br>Ticked = 1<br>Not ticked = 0	Q10b_2_18<br>1: under supervision<br>2: shared responsibility<br>3: full autonomy
other task within management of therapeutic and adverse effects of medicines: PLEASE SPECIFY	Q10b_3_19_1<br>Ticked = 1<br>Not ticked = 0<br>Q10b_3_19_TEXT	Q10b_1_19_1<br>Ticked = 1<br>Not ticked = 0<br>Q10b_1_19_TEXT	Q10b_2_19<br>1: under supervision<br>2: shared responsibility<br>3: full autonomy<br>Q10b_2_19_TEXT

Q11b/ Responsibility 2: management of medicines adherence.

	**Task not relevant** within management of medicines adherence.	**Task not allowed** for nurses within management of medicines adherence.	**Task allowed** for nurses within management of medicines adherence. Specify the level of responsibility.
observation, documentation, registration, report	**Q11b_3_1_1**<br>Ticked = 1<br>Not ticked = 0	**Q11b_1_1_1**<br>Ticked = 1<br>Not ticked = 0	**Q11b_2_1**<br>1: under supervision<br>2: shared responsibility<br>3: full autonomy
assessing patients competences	**Q11b_3_2_1**<br>Ticked = 1<br>Not ticked = 0	**Q11b_1_2_1**<br>Ticked = 1<br>Not ticked = 0	**Q11b_2_2**<br>1: under supervision<br>2: shared responsibility<br>3: full autonomy
assessment and addressing patient/family needs	**Q10b_3_3_1**<br>Ticked = 1<br>Not ticked = 0	**Q11b_1_3_1**<br>Ticked = 1<br>Not ticked = 0	**Q11b_2_3**<br>1: under supervision<br>2: shared responsibility<br>3: full autonomy
recognising & preventing risks / complications / medication errors	**Q10b_3_4_1**<br>Ticked = 1<br>Not ticked = 0	**Q11b_1_4_1**<br>Ticked = 1<br>Not ticked = 0	**Q11b_2_4**<br>1: under supervision<br>2: shared responsibility<br>3: full autonomy
identification, reporting and addressing contra-indications, drug related problems	**Q10b_3_5_1**<br>Ticked = 1<br>Not ticked = 0	**Q11b_1_5_1**<br>Ticked = 1<br>Not ticked = 0	**Q11b_2_5**<br>1: under supervision<br>2: shared responsibility<br>3: full autonomy
follow-up	**Q10b_3_6_1**<br>Ticked = 1<br>Not ticked = 0	**Q11b_1_6_1**<br>Ticked = 1<br>Not ticked = 0	**Q11b_2_6**<br>1: under supervision<br>2: shared responsibility<br>3: full autonomy
evidence based practice	**Q10b_3_7_1**<br>Ticked = 1<br>Not ticked = 0	**Q11b_1_7_1**<br>Ticked = 1<br>Not ticked = 0	**Q11b_2_7**<br>1: under supervision<br>2: shared responsibility<br>3: full autonomy
decision making	**Q10b_3_8_1**<br>Ticked = 1<br>Not ticked = 0	**Q11b_1_8_1**<br>Ticked = 1<br>Not ticked = 0	**Q11b_2_8**<br>1: under supervision<br>2: shared responsibility<br>3: full autonomy
communication /discussion with patient/family	**Q10b_3_9_1**<br>Ticked = 1<br>Not ticked = 0	**Q11b_1_9_1**<br>Ticked = 1<br>Not ticked = 0	**Q11b_2_9**<br>1: under supervision<br>2: shared responsibility<br>3: full autonomy
detection of non-adherence, drug abuse/misuse	**Q11b_3_10_1**<br>Ticked = 1<br>Not ticked = 0	**Q11b_1_10_1**<br>Ticked = 1<br>Not ticked = 0	**Q11b_2_10**<br>1: under supervision<br>2: shared responsibility<br>3: full autonomy
motivational interviewing	**Q11b_3_11_1**<br>Ticked = 1	**Q11b_1_11_1**<br>Ticked = 1	**Q11b_2_11**<br>1: under supervision

	Not ticked = 0	Not ticked = 0	2: shared responsibility<br>3: full autonomy
Inter / intraprofessional referrals	**Q11b_3_12_1**<br>Ticked = 1<br>Not ticked = 0	**Q11b_1_12_1**<br>Ticked = 1<br>Not ticked = 0	**Q11b_2_12**<br>1: under supervision<br>2: shared responsibility<br>3: full autonomy
facilitation of medication management	**Q11b_3_14_1**<br>Ticked = 1<br>Not ticked = 0	**Q11b_1_14_1**<br>Ticked = 1<br>Not ticked = 0	**Q11b_2_14**<br>1: under supervision<br>2: shared responsibility<br>3: full autonomy
selfcare support and therapeutic education	**Q11b_3_17_1**<br>Ticked = 1<br>Not ticked = 0	**Q11b_1_17_1**<br>Ticked = 1<br>Not ticked = 0	**Q11b_2_17**<br>1: under supervision<br>2: shared responsibility<br>3: full autonomy
advice (patient or other healthcare professional)	**Q11b_3_18_1**<br>Ticked = 1<br>Not ticked = 0	**Q11b_1_18_1**<br>Ticked = 1<br>Not ticked = 0	**Q11b_2_18**<br>1: under supervision<br>2: shared responsibility<br>3: full autonomy
transitional care communication, inter / intraprofessional collaboration / communication including reporting, advising, informing, alerting and discussing	**Q11b_3_19_1**<br>Ticked = 1<br>Not ticked = 0	**Q11b_1_19_1**<br>Ticked = 1<br>Not ticked = 0	**Q11b_2_19**<br>1: under supervision<br>2: shared responsibility<br>3: full autonomy
collegial mentoring	**Q11b_3_20_1**<br>Ticked = 1<br>Not ticked = 0	**Q11b_1_20_1**<br>Ticked = 1<br>Not ticked = 0	**Q11b_2_20**<br>1: under supervision<br>2: shared responsibility<br>3: full autonomy
other task within management of medicines adherence:	**Q11b_3_21_1**<br>Ticked = 1<br>Not ticked = 0<br>**Q11b_3_21_TEXT**	**Q11b_1_21_1**<br>Ticked = 1<br>Not ticked = 0<br>**Q11b_1_21_TEXT**	**Q11b_2_21**<br>1: under supervision<br>2: shared responsibility<br>3: full autonomy<br>**Q11b_2_21_TEXT**

Q12b/ Responsibility 3: management of patient medication self-management.

	**Task not relevant** within management of patient medication self-management.	**Task not allowed** for nurses within management of patient medication self-management.	**Task allowed** for nurses within management of patient medication self-management. Specify the level of responsibility.
observation, documentation, registration, report	**Q12b_3_1_1**<br>Ticked = 1<br>Not ticked = 0	**Q12b_1_1_1**<br>Ticked = 1<br>Not ticked = 0	**Q12b_2_1**<br>1: under supervision<br>2: shared responsibility<br>3: full autonomy
assessing patients competences	**Q12b_3_2_1**	**Q12b_1_2_1**	**Q12b_2_2**

	Ticked = 1<br>Not ticked = 0	Ticked = 1<br>Not ticked = 0	1: under supervision<br>2: shared responsibility<br>3: full autonomy
assessment and addressing patient/family needs	Q12b_3_3_1<br>Ticked = 1<br>Not ticked = 0	Q12b_1_3_1<br>Ticked = 1<br>Not ticked = 0	Q12b_2_3<br>1: under supervision<br>2: shared responsibility<br>3: full autonomy
recognising & preventing risks / complications / medication errors	Q12b_3_4_1<br>Ticked = 1<br>Not ticked = 0	Q12b_1_4_1<br>Ticked = 1<br>Not ticked = 0	Q12b_2_4<br>1: under supervision<br>2: shared responsibility<br>3: full autonomy
identification, reporting and addressing contra-indications, drug related problems	Q12b_3_5_1<br>Ticked = 1<br>Not ticked = 0	Q12b_1_5_1<br>Ticked = 1<br>Not ticked = 0	Q12b_2_5<br>1: under supervision<br>2: shared responsibility<br>3: full autonomy
follow-up	Q12b_3_6_1<br>Ticked = 1<br>Not ticked = 0	Q12b_1_6_1<br>Ticked = 1<br>Not ticked = 0	Q12b_2_6<br>1: under supervision<br>2: shared responsibility<br>3: full autonomy
evidence based practice	Q12b_3_7_1<br>Ticked = 1<br>Not ticked = 0	Q12b_1_7_1<br>Ticked = 1<br>Not ticked = 0	Q12b_2_7<br>1: under supervision<br>2: shared responsibility<br>3: full autonomy
decision making	Q12b_3_8_1<br>Ticked = 1<br>Not ticked = 0	Q12b_1_8_1<br>Ticked = 1<br>Not ticked = 0	Q12b_2_8<br>1: under supervision<br>2: shared responsibility<br>3: full autonomy
communication /discussion with patient/family	Q12b_3_9_1<br>Ticked = 1<br>Not ticked = 0	Q12b_1_9_1<br>Ticked = 1<br>Not ticked = 0	Q12b_2_9<br>1: under supervision<br>2: shared responsibility<br>3: full autonomy
motivational interviewing	Q12b_3_10_1<br>Ticked = 1<br>Not ticked = 0	Q12b_1_10_1<br>Ticked = 1<br>Not ticked = 0	Q12b_2_10<br>1: under supervision<br>2: shared responsibility<br>3: full autonomy
Inter / intraprofessional referrals	Q12b_3_11_1<br>Ticked = 1<br>Not ticked = 0	Q12b_1_11_1<br>Ticked = 1<br>Not ticked = 0	Q12b_2_11<br>1: under supervision<br>2: shared responsibility<br>3: full autonomy
facilitation of medication management	Q12b_3_13_1<br>Ticked = 1<br>Not ticked = 0	Q12b_1_13_1<br>Ticked = 1<br>Not ticked = 0	Q12b_2_13<br>1: under supervision<br>2: shared responsibility<br>3: full autonomy
selfcare support and therapeutic education	Q12b_3_16_1<br>Ticked = 1<br>Not ticked = 0	Q12b_1_16_1<br>Ticked = 1<br>Not ticked = 0	Q12b_2_16<br>1: under supervision<br>2: shared responsibility<br>3: full autonomy
advice (patient or other healthcare professional)	Q12b_3_17_1<br>Ticked = 1<br>Not ticked = 0	Q12b_1_17_1<br>Ticked = 1<br>Not ticked = 0	Q12b_2_17<br>1: under supervision<br>2: shared responsibility

			3: full autonomy
transitional care communication, inter / intraprofessional collaboration / communication including reporting, advising, informing, alerting and discussing	Q12b_3_18_1<br>Ticked = 1<br>Not ticked = 0	Q12b_1_18_1<br>Ticked = 1<br>Not ticked = 0	Q12b_2_18<br>1: under supervision<br>2: shared responsibility<br>3: full autonomy
collegial mentoring	Q12b_3_19_1<br>Ticked = 1<br>Not ticked = 0	Q12b_1_19_1<br>Ticked = 1<br>Not ticked = 0	Q12b_2_19<br>1: under supervision<br>2: shared responsibility<br>3: full autonomy
other task within management of patient medication self-management:	Q12b_3_20_1<br>Ticked = 1<br>Not ticked = 0<br>Q12b_3_20_TEXT	Q12b_1_20_1<br>Ticked = 1<br>Not ticked = 0<br>Q12b_1_20_TEXT	Q12b_2_20<br>1: under supervision<br>2: shared responsibility<br>3: full autonomy<br>Q12b_2_20_TEXT

Q13b/ Responsibility 4: management of patient education and information.

	**Task not relevant** within management of patient education and information.	**Task not allowed** for nurses within management of patient education and information.	**Task allowed** for nurses within management of patient education and information. Specify the level of responsibility.
observation, documentation, registration, report	Q13b_3_1_1<br>Ticked = 1<br>Not ticked = 0	Q13b_1_1_1<br>Ticked = 1<br>Not ticked = 0	Q13b_2_1<br>1: under supervision<br>2: shared responsibility<br>3: full autonomy
assessing patients competences	Q13b_3_2_1<br>Ticked = 1<br>Not ticked = 0	Q13b_1_2_1<br>Ticked = 1<br>Not ticked = 0	Q13b_2_2<br>1: under supervision<br>2: shared responsibility<br>3: full autonomy
assessment and addressing patient/family needs	Q13b_3_3_1<br>Ticked = 1<br>Not ticked = 0	Q13b_1_3_1<br>Ticked = 1<br>Not ticked = 0	Q13b_2_3<br>1: under supervision<br>2: shared responsibility<br>3: full autonomy
follow-up	Q13b_3_4_1<br>Ticked = 1<br>Not ticked = 0	Q13b_1_4_1<br>Ticked = 1<br>Not ticked = 0	Q13b_2_4<br>1: under supervision<br>2: shared responsibility<br>3: full autonomy
evidence based practice	Q13b_3_5_1<br>Ticked = 1<br>Not ticked = 0	Q13b_1_5_1<br>Ticked = 1<br>Not ticked = 0	Q13b_2_5<br>1: under supervision<br>2: shared responsibility<br>3: full autonomy
decision making	Q13b_3_6_1	Q13b_1_6_1	Q13b_2_6

	Ticked = 1<br>Not ticked = 0	Ticked = 1<br>Not ticked = 0	1: under supervision<br>2: shared responsibility<br>3: full autonomy
communication /discussion with patient/family	Q13b_3_7_1<br>Ticked = 1<br>Not ticked = 0	Q13b_1_7_1<br>Ticked = 1<br>Not ticked = 0	Q13b_2_7<br>1: under supervision<br>2: shared responsibility<br>3: full autonomy
motivational interviewing	Q13b_3_8_1<br>Ticked = 1<br>Not ticked = 0	Q13b_1_8_1<br>Ticked = 1<br>Not ticked = 0	Q13b_2_8<br>1: under supervision<br>2: shared responsibility<br>3: full autonomy
Inter / intraprofessional referrals	Q13b_3_9_1<br>Ticked = 1<br>Not ticked = 0	Q13b_1_9_1<br>Ticked = 1<br>Not ticked = 0	Q13b_2_9<br>1: under supervision<br>2: shared responsibility<br>3: full autonomy
facilitation of medication management	Q13b_3_11_1<br>Ticked = 1<br>Not ticked = 0	Q13b_1_11_1<br>Ticked = 1<br>Not ticked = 0	Q13b_2_11<br>1: under supervision<br>2: shared responsibility<br>3: full autonomy
selfcare support and therapeutic education	Q13b_3_14_1<br>Ticked = 1<br>Not ticked = 0	Q13b_1_14_1<br>Ticked = 1<br>Not ticked = 0	Q13b_2_14<br>1: under supervision<br>2: shared responsibility<br>3: full autonomy
advice (patient or other healthcare professional)	Q13b_3_15_1<br>Ticked = 1<br>Not ticked = 0	Q13b_1_15_1<br>Ticked = 1<br>Not ticked = 0	Q13b_2_15<br>1: under supervision<br>2: shared responsibility<br>3: full autonomy
transitional care communication, inter / intraprofessional collaboration / communication including reporting, advising, informing, alerting and discussing	Q13b_3_16_1<br>Ticked = 1<br>Not ticked = 0	Q13b_1_16_1<br>Ticked = 1<br>Not ticked = 0	Q13b_2_16<br>1: under supervision<br>2: shared responsibility<br>3: full autonomy
collegial mentoring	Q13b_3_17_1<br>Ticked = 1<br>Not ticked = 0	Q13b_1_17_1<br>Ticked = 1<br>Not ticked = 0	Q13b_2_17<br>1: under supervision<br>2: shared responsibility<br>3: full autonomy
other task within management of patient education and information:	Q13b_3_18_1<br>Ticked = 1<br>Not ticked = 0<br>Q13b_3_18_TEXT	Q13b_1_18_1<br>Ticked = 1<br>Not ticked = 0<br>Q13b_1_18_TEXT	Q13b_2_18<br>1: under supervision<br>2: shared responsibility<br>3: full autonomy<br>Q13b_2_18_TEXT

Q14b/ Responsibility 5: prescription management.

	**Task not relevant** within prescription management.	**Task not allowed** for nurses within prescription management.	**Task allowed** for nurses within prescription management. Specify the level of responsibility.
observation, documentation, registration, report	**Q14b_3_1_1**<br>Ticked = 1<br>Not ticked = 0	**Q14b_1_1_1**<br>Ticked = 1<br>Not ticked = 0	**Q14b_2_1**<br>1: under supervision<br>2: shared responsibility<br>3: full autonomy
assessing patients competences	**Q14b_3_2_1**<br>Ticked = 1<br>Not ticked = 0	**Q14b_1_2_1**<br>Ticked = 1<br>Not ticked = 0	**Q14b_2_2**<br>1: under supervision<br>2: shared responsibility<br>3: full autonomy
assessment and addressing patient/family needs	**Q14b_3_3_1**<br>Ticked = 1<br>Not ticked = 0	**Q14b_1_3_1**<br>Ticked = 1<br>Not ticked = 0	**Q14b_2_3**<br>1: under supervision<br>2: shared responsibility<br>3: full autonomy
recognising & preventing risks / complications / medication errors	**Q14b_3_4_1**<br>Ticked = 1<br>Not ticked = 0	**Q14b_1_4_1**<br>Ticked = 1<br>Not ticked = 0	**Q14b_2_4**<br>1: under supervision<br>2: shared responsibility<br>3: full autonomy
identification, reporting and addressing contra-indications, drug related problems	**Q14b_3_5_1**<br>Ticked = 1<br>Not ticked = 0	**Q14b_1_5_1**<br>Ticked = 1<br>Not ticked = 0	**Q14b_2_5**<br>1: under supervision<br>2: shared responsibility<br>3: full autonomy
follow-up	**Q14b_3_6_1**<br>Ticked = 1<br>Not ticked = 0	**Q14b_1_6_1**<br>Ticked = 1<br>Not ticked = 0	**Q14b_2_6**<br>1: under supervision<br>2: shared responsibility<br>3: full autonomy
evidence based practice	**Q14b_3_7_1**<br>Ticked = 1<br>Not ticked = 0	**Q14b_1_7_1**<br>Ticked = 1<br>Not ticked = 0	**Q14b_2_7**<br>1: under supervision<br>2: shared responsibility<br>3: full autonomy
decision making	**Q14b_3_8_1**<br>Ticked = 1<br>Not ticked = 0	**Q14b_1_8_1**<br>Ticked = 1<br>Not ticked = 0	**Q14b_2_8**<br>1: under supervision<br>2: shared responsibility<br>3: full autonomy
communication /discussion with patient/family	**Q14b_3_9_1**<br>Ticked = 1<br>Not ticked = 0	**Q14b_1_9_1**<br>Ticked = 1<br>Not ticked = 0	**Q14b_2_9**<br>1: under supervision<br>2: shared responsibility<br>3: full autonomy
Inter / intraprofessional referrals	**Q14b_3_10_1**<br>Ticked = 1<br>Not ticked = 0	**Q14b_1_10_1**<br>Ticked = 1<br>Not ticked = 0	**Q14b_2_10**<br>1: under supervision<br>2: shared responsibility<br>3: full autonomy
facilitation of medication management	**Q14b_3_12_1**<br>Ticked = 1<br>Not ticked = 0	**Q14b_1_12_1**<br>Ticked = 1<br>Not ticked = 0	**Q14b_2_12**<br>1: under supervision<br>2: shared responsibility<br>3: full autonomy

advice (patient or other healthcare professional)	Q14b_3_14_1<br>Ticked = 1<br>Not ticked = 0	Q14b_1_14_1<br>Ticked = 1<br>Not ticked = 0	Q14b_2_14<br>1: under supervision<br>2: shared responsibility<br>3: full autonomy
determination of type/dosage	Q14b_3_15_1<br>Ticked = 1<br>Not ticked = 0	Q14b_1_15_1<br>Ticked = 1<br>Not ticked = 0	Q14b_2_15<br>1: under supervision<br>2: shared responsibility<br>3: full autonomy
initiation of medication (reactive / proactive)	Q14b_3_16_1<br>Ticked = 1<br>Not ticked = 0	Q14b_1_16_1<br>Ticked = 1<br>Not ticked = 0	Q14b_2_16<br>1: under supervision<br>2: shared responsibility<br>3: full autonomy
adaptation of dose, dose titration	Q14b_3_17_1<br>Ticked = 1<br>Not ticked = 0	Q14b_1_17_1<br>Ticked = 1<br>Not ticked = 0	Q14b_2_17<br>1: under supervision<br>2: shared responsibility<br>3: full autonomy
decision on continuation /cessation of medication	Q14b_3_18_1<br>Ticked = 1<br>Not ticked = 0	Q14b_1_18_1<br>Ticked = 1<br>Not ticked = 0	Q14b_2_18<br>1: under supervision<br>2: shared responsibility<br>3: full autonomy
PRN (pro re nata, 'if needed' medication) / standing prescription renewal order	Q14b_3_19_1<br>Ticked = 1<br>Not ticked = 0	Q14b_1_19_1<br>Ticked = 1<br>Not ticked = 0	Q14b_2_19<br>1: under supervision<br>2: shared responsibility<br>3: full autonomy
medication reconciliation	Q14b_3_20_1<br>Ticked = 1<br>Not ticked = 0	Q14b_1_20_1<br>Ticked = 1<br>Not ticked = 0	Q14b_2_20<br>1: under supervision<br>2: shared responsibility<br>3: full autonomy
medication review	Q14b_3_21_1<br>Ticked = 1<br>Not ticked = 0	Q14b_1_21_1<br>Ticked = 1<br>Not ticked = 0	Q14b_2_21<br>1: under supervision<br>2: shared responsibility<br>3: full autonomy
intervention in case of emergency	Q14b_3_22_1<br>Ticked = 1<br>Not ticked = 0	Q14b_1_22_1<br>Ticked = 1<br>Not ticked = 0	Q14b_2_22<br>1: under supervision<br>2: shared responsibility<br>3: full autonomy
transitional care communication, inter / intraprofessional collaboration / communication including reporting, advising, informing, alerting and discussing	Q14b_3_23_1<br>Ticked = 1<br>Not ticked = 0	Q14b_1_23_1<br>Ticked = 1<br>Not ticked = 0	Q14b_2_23<br>1: under supervision<br>2: shared responsibility<br>3: full autonomy
collegial mentoring	Q14b_3_24_1<br>Ticked = 1<br>Not ticked = 0	Q14b_1_24_1<br>Ticked = 1<br>Not ticked = 0	Q14b_2_24<br>1: under supervision<br>2: shared responsibility<br>3: full autonomy
other task within prescription management:	Q14b_3_25_1<br>Ticked = 1<br>Not ticked = 0	Q14b_1_25_1<br>Ticked = 1<br>Not ticked = 0	Q14b_2_25<br>1: under supervision<br>2: shared responsibility

	Q14b_3_25_TEXT	Q14b_1_25_TEXT	3: full autonomy<br>Q14b_2_25_TEXT

Q17b/ Responsibility 6: medication safety management.

	**Task not relevant** within medication safety management.	**Task not allowed** for nurses within medication safety management.	**Task allowed** for nurses within medication safety management. Specify the level of responsibility.
observation, documentation, registration, report	Q17b_3_1_1<br>Ticked = 1<br>Not ticked = 0	Q17b_1_1_1<br>Ticked = 1<br>Not ticked = 0	Q17b_2_1<br>1: under supervision<br>2: shared responsibility<br>3: full autonomy
assessing patients competences	Q17b_3_2_1<br>Ticked = 1<br>Not ticked = 0	Q17b_1_2_1<br>Ticked = 1<br>Not ticked = 0	Q17b_2_2<br>1: under supervision<br>2: shared responsibility<br>3: full autonomy
assessment and addressing patient/family needs	Q17b_3_3_1<br>Ticked = 1<br>Not ticked = 0	Q17b_1_3_1<br>Ticked = 1<br>Not ticked = 0	Q17b_2_3<br>1: under supervision<br>2: shared responsibility<br>3: full autonomy
recognising & preventing risks / complications / medication errors	Q17b_3_4_1<br>Ticked = 1<br>Not ticked = 0	Q17b_1_4_1<br>Ticked = 1<br>Not ticked = 0	Q17b_2_4<br>1: under supervision<br>2: shared responsibility<br>3: full autonomy
identification, reporting and addressing contra-indications, drug related problems	Q17b_3_5_1<br>Ticked = 1<br>Not ticked = 0	Q17b_1_5_1<br>Ticked = 1<br>Not ticked = 0	Q17b_2_5<br>1: under supervision<br>2: shared responsibility<br>3: full autonomy
follow-up	Q17b_3_6_1<br>Ticked = 1<br>Not ticked = 0	Q17b_1_6_1<br>Ticked = 1<br>Not ticked = 0	Q17b_2_6<br>1: under supervision<br>2: shared responsibility<br>3: full autonomy
evidence based practice	Q17b_3_7_1<br>Ticked = 1<br>Not ticked = 0	Q17b_1_7_1<br>Ticked = 1<br>Not ticked = 0	Q17b_2_7<br>1: under supervision<br>2: shared responsibility<br>3: full autonomy
decision making	Q17b_3_8_1<br>Ticked = 1<br>Not ticked = 0	Q17b_1_8_1<br>Ticked = 1<br>Not ticked = 0	Q17b_2_8<br>1: under supervision<br>2: shared responsibility<br>3: full autonomy
communication /discussion with patient/family	Q17b_3_9_1<br>Ticked = 1<br>Not ticked = 0	Q17b_1_9_1<br>Ticked = 1<br>Not ticked = 0	Q17b_2_9<br>1: under supervision<br>2: shared responsibility<br>3: full autonomy

Inter / intraprofessional referrals	Q17b_3_10_1<br>Ticked = 1<br>Not ticked = 0	Q17b_1_10_1<br>Ticked = 1<br>Not ticked = 0	Q17b_2_10<br>1: under supervision<br>2: shared responsibility<br>3: full autonomy
facilitation of medication management	Q17b_3_12_1<br>Ticked = 1<br>Not ticked = 0	Q17b_1_12_1<br>Ticked = 1<br>Not ticked = 0	Q17b_2_12<br>1: under supervision<br>2: shared responsibility<br>3: full autonomy
selfcare support and therapeutic education	Q17b_3_15_1<br>Ticked = 1<br>Not ticked = 0	Q17b_1_15_1<br>Ticked = 1<br>Not ticked = 0	Q17b_2_15<br>1: under supervision<br>2: shared responsibility<br>3: full autonomy
advice (patient or other healthcare professional)	Q17b_3_16_1<br>Ticked = 1<br>Not ticked = 0	Q17b_1_16_1<br>Ticked = 1<br>Not ticked = 0	Q17b_2_16<br>1: under supervision<br>2: shared responsibility<br>3: full autonomy
intervention in case of emergency	Q17b_3_17_1<br>Ticked = 1<br>Not ticked = 0	Q17b_1_17_1<br>Ticked = 1<br>Not ticked = 0	Q17b_2_17<br>1: under supervision<br>2: shared responsibility<br>3: full autonomy
transitional care communication, inter / intraprofessional collaboration / communication including reporting, advising, informing, alerting and discussing	Q17b_3_18_1<br>Ticked = 1<br>Not ticked = 0	Q17b_1_18_1<br>Ticked = 1<br>Not ticked = 0	Q17b_2_18<br>1: under supervision<br>2: shared responsibility<br>3: full autonomy
collegial mentoring	Q17b_3_19_1<br>Ticked = 1<br>Not ticked = 0	Q17b_1_19_1<br>Ticked = 1<br>Not ticked = 0	Q17b_2_19<br>1: under supervision<br>2: shared responsibility<br>3: full autonomy
other task within medication safety management:	Q17b_3_20_1<br>Ticked = 1<br>Not ticked = 0<br>Q17b_3_20_TEXT	Q17b_1_20_1<br>Ticked = 1<br>Not ticked = 0<br>Q17b_1_20_TEXT	Q17b_2_20<br>1: under supervision<br>2: shared responsibility<br>3: full autonomy<br>Q17b_2_20_TEXT

Q18b/ Responsibility 7: (transition of) care coordination.

	**Task not relevant** within (transition of) care coordination.	**Task not allowed** for nurses within (transition of) care coordination.	**Task allowed** for nurses within (transition of) care coordination. Specify the level of responsibility.

observation, documentation, registration, report	Q18b_3_1_1<br>Ticked = 1<br>Not ticked = 0	Q18b_1_1_1<br>Ticked = 1<br>Not ticked = 0	Q18b_2_1<br>1: under supervision<br>2: shared responsibility<br>3: full autonomy
assessing patients competences	Q18b_3_2_1<br>Ticked = 1<br>Not ticked = 0	Q18b_1_2_1<br>Ticked = 1<br>Not ticked = 0	Q18b_2_2<br>1: under supervision<br>2: shared responsibility<br>3: full autonomy
assessment and addressing patient/family needs	Q18b_3_3_1<br>Ticked = 1<br>Not ticked = 0	Q18b_1_3_1<br>Ticked = 1<br>Not ticked = 0	Q18b_2_3<br>1: under supervision<br>2: shared responsibility<br>3: full autonomy
recognising & preventing risks / complications / medication errors	Q18b_3_4_1<br>Ticked = 1<br>Not ticked = 0	Q18b_1_4_1<br>Ticked = 1<br>Not ticked = 0	Q18b_2_4<br>1: under supervision<br>2: shared responsibility<br>3: full autonomy
identification, reporting and addressing contra-indications, drug related problems	Q18b_3_5_1<br>Ticked = 1<br>Not ticked = 0	Q18b_1_5_1<br>Ticked = 1<br>Not ticked = 0	Q18b_2_5<br>1: under supervision<br>2: shared responsibility<br>3: full autonomy
follow-up	Q18b_3_6_1<br>Ticked = 1<br>Not ticked = 0	Q18b_1_6_1<br>Ticked = 1<br>Not ticked = 0	Q18b_2_6<br>1: under supervision<br>2: shared responsibility<br>3: full autonomy
evidence based practice	Q18b_3_7_1<br>Ticked = 1<br>Not ticked = 0	Q18b_1_7_1<br>Ticked = 1<br>Not ticked = 0	Q18b_2_7<br>1: under supervision<br>2: shared responsibility<br>3: full autonomy
decision making	Q18b_3_8_1<br>Ticked = 1<br>Not ticked = 0	Q18b_1_8_1<br>Ticked = 1<br>Not ticked = 0	Q18b_2_8<br>1: under supervision<br>2: shared responsibility<br>3: full autonomy
communication /discussion with patient/family	Q18b_3_9_1<br>Ticked = 1<br>Not ticked = 0	Q18b_1_9_1<br>Ticked = 1<br>Not ticked = 0	Q18b_2_9<br>1: under supervision<br>2: shared responsibility<br>3: full autonomy
Inter / intraprofessional referrals	Q18b_3_10_1<br>Ticked = 1<br>Not ticked = 0	Q18b_1_10_1<br>Ticked = 1<br>Not ticked = 0	Q18b_2_10<br>1: under supervision<br>2: shared responsibility<br>3: full autonomy
facilitation of medication management	Q18b_3_12_1<br>Ticked = 1<br>Not ticked = 0	Q18b_1_12_1<br>Ticked = 1<br>Not ticked = 0	Q18b_2_12<br>1: under supervision<br>2: shared responsibility<br>3: full autonomy
selfcare support and therapeutic education	Q18b_3_15_1<br>Ticked = 1<br>Not ticked = 0	Q18b_1_15_1<br>Ticked = 1<br>Not ticked = 0	Q18b_2_15<br>1: under supervision<br>2: shared responsibility<br>3: full autonomy
advice (patient or other healthcare professional)	Q18b_3_16_1	Q18b_1_16_1	Q18b_2_16<br>1: under supervision

	Ticked = 1<br>Not ticked = 0	Ticked = 1<br>Not ticked = 0	2: shared responsibility<br>3: full autonomy
discharge planning, transition of care planning	**Q18b_3_17_1**<br>Ticked = 1<br>Not ticked = 0	**Q18b_1_17_1**<br>Ticked = 1<br>Not ticked = 0	**Q18b_2_17**<br>1: under supervision<br>2: shared responsibility<br>3: full autonomy
transitional care communication, inter / intraprofessional collaboration / communication including reporting, advising, informing, alerting and discussing	**Q18b_3_18_1**<br>Ticked = 1<br>Not ticked = 0	**Q18b_1_18_1**<br>Ticked = 1<br>Not ticked = 0	**Q18b_2_18**<br>1: under supervision<br>2: shared responsibility<br>3: full autonomy
collegial mentoring	**Q18b_3_19_1**<br>Ticked = 1<br>Not ticked = 0	**Q18b_1_19_1**<br>Ticked = 1<br>Not ticked = 0	**Q18b_2_19**<br>1: under supervision<br>2: shared responsibility<br>3: full autonomy
other task within (transition of) care coordination:	**Q18b_3_20_1**<br>Ticked = 1<br>Not ticked = 0<br>**Q18b_3_20_TEXT**	**Q18b_1_20_1**<br>Ticked = 1<br>Not ticked = 0<br>**Q18b_1_20_TEXT**	**Q18b_2_20**<br>1: under supervision<br>2: shared responsibility<br>3: full autonomy<br>**Q18b_2_20_TEXT**

Q20/

In your current context, on balance, are the following factors **barriers or enablers** of nurses' roles in interprofessional pharmaceutical care? Indicate your opinion on a scale from -5 to +5 (-5 = a great barrier, 0 = no influence on nurses' responsibilities or tasks, +5 = a great enabler).

	-5 -4 -3 -2 -1 0 1 2 3 4 5
Level of emergency	**Q20_1**
Level of nurse education	**Q20_2**
Quality of nurse education	**Q20_3**
Interprofessional education	**Q20_4**
Adequate reimbursement	**Q20_5**
Clarity of interprofessional team members’ roles	**Q20_6**
Availability of interprofessional team members	**Q20_7**
Collaborative approach between nurses, pharmacists and prescribers	**Q20_8**
Workload/time to care	**Q20_9**
Adequate nurse to patient ratio	**Q20_10**
Shared digital / electronic patient files and records	**Q20_11**
Legal framework in your country	**Q20_12**

(Self-)confidence of nurses	**Q20_13**
Readiness of healthcare workers and patients to accept nurse prescribing	**Q20_14**
Open, blame-free safety culture	**Q20_15**
Implementation strategy for nurse prescribing	**Q20_16**
Healthcare setting	**Q20_17**
Epidemiological context (e.g. demographics, patient needs, professional needs,…)	**Q20_18**
Ethical context (e.g. norms, values,…)	**Q20_19**
Political context	**Q20_20**
