# Supplementary material for: The NUPHAC-EU Framework for Nurses’ Role in Interprofessional Pharmaceutical Care: Cross-Sectional Evaluation in Europe
Source: Int J Environ Res Public Health. 2021 Jul 25;18(15):7862. doi: 10.3390/ijerph18157862 (PMC8345454; doi:10.3390/ijerph18157862)
Supplement: Supplementary file 1 [file ijerph-18-07862-s001.zip › Supplementary File_S3_table.pdf]

Supplementary Table S1: Percentages of healthcare professionals considering 26 tasks in seven pharmaceutical care domains as nurses' tasks in order to obtain best quality of care and patient outcomes, split up for 4 EQF* levels of nurse education.

**PHARMACEUTICAL CARE DOMAIN 1: MANAGEMENT OF THERAPEUTIC AND ADVERSE EFFECTS OF MEDICINES (n=731)**						
**Task within 'management of therapeutic and adverse effects of medicines'**	Degree of responsibility for nurses performing this task	**All nurses** (no distinction between levels of education)[a]	**level 5 nurses**	**level 6 nurses**	**level 7 nurses**	**level 8 nurses**
		% (n)	% (n)	% (n)	% (n)	% (n)
observation, documentation, registration, reporting	not allowed	3.6 (28)	1.4 (5)	0.3 (1)	0 (0)	0 (0)
	under supervision	19.4 (149)	11.9 (43)	11.4 (42)	10.1 (37)	10.0 (36)
	shared responsibility	31.8 (245)	29.6 (107)	28.8 (106)	28.8 (106)	28.3 (102)
	full autonomy	**45.2** (348)	**57.1** (206)	**59.5** (219)	**61.1** (225)	**61.8** (223)
assessing patients' competences	not allowed	5.1 (38)	0.9 (3)	0 (0)	0 (0)	0 (0)
	under supervision	17.5 (131)	9.2 (32)	8.2 (29)	6.2 (22)	6.3 (22)
	shared responsibility	36.1 (240)	38.0 (132)	35.5 (126)	37.9 (134)	35.4 (123)
	full autonomy	**41.2** (308)	**51.9** (180)	**56.3** (200)	**55.9** (198)	**58.2** (202)
assessment and addressing patient/family needs	not allowed	4.2 (31)	1.1 (4)	0 (0)	0 (0)	0 (0)
	under supervision	16.8 (124)	10.9 (328)	10.1 (36)	7.9 (28)	7.7 (27)
	shared responsibility	**39.7** (293)	40.9 (143)	38.3 (136)	40.3 (143)	28.7 (135)
	full autonomy	39.3 (290)	**47.1** (165)	**51.5** (183)	**51.8** (184)	**53.6** (187)
recognising & preventing risks / complications / medication errors	not allowed	5.4 (40)	2.0 (7)	0 (0)	0 (0)	0 (0)
	under supervision	21.5 (160)	15.5 (54)	14.9 (53)	11.4 (40)	11.2 (39)
	shared responsibility	**46.3** (345)	**45.4** (158)	**46.1** (164)	**45.7** (161)	43.8 (153)
	full autonomy	16.8 (200)	37.1 (129)	39.0 (139)	42.9 (151)	**45.0** (157)
identification, reporting and addressing contra-indications to administration, identification of drug related problems	not allowed	10.6 (79)	2.7 (9)	0.9 (3)	0 (0)	0 (0)
	under supervision	23.7 (177)	20.6 (69)	17.1 (59)	15.2 (52)	14.4 (48)
	shared responsibility	**47.6** (356)	**49.6** (166)	52.**2** (180)	**52.8** (180)	**51.2** (171)
	full autonomy	18.2 (136)	27.2 (91)	29.9 (103)	32.0 (109)	34.4 (115)
follow-up	not allowed	7.5 (56)	2.1 (7)	0.6 (2)	0.3 (1)	0 (0)
	under supervision	19.7 (147)	12.8 (43)	11.0 (38)	9.4 (32)	8.9 (30)
	shared responsibility	**41.5** (310)	**44.2** (149)	**45.4** (157)	**45.5** (155)	43.8 (147)
	full autonomy	31.3 (234)	40.9 (138)	43.1 (149)	44.9 (153)	**47.3** (159)
adhering to evidence based practice as described in clinical guidelines	not allowed	8.4 (60)	5.3 (17)	2.1 (7)	0.6 (2)	0 (0)
	under supervision	23.9 (171)	18.0 (58)	15.9 (53)	12.4 (41)	11.7 (38)
	shared responsibility	**38.5** (275)	**40.7** (131)	**42.6** (142)	42.1 (139)	40.7 (132)
	full autonomy	29.1 (208)	36.0 (116)	39.3 (131)	**44.8** (148)	**47.5** (154)
decision making	not allowed	17.9 (133)	4.3 (13)	0.6 (2)	0.3 (1)	0.3 (1)
	under supervision	27.4 (203)	23.6 (71)	22.8 (72)	19.8 (62)	19.2 (59)

	shared responsibility<br>full autonomy	**40.4** (299)<br>14.3 (106)	**52.2** (157)<br>19.9 (60)	**52.5** (166)<br>24.1 (76)	**52.7** (165)<br>27.2 (85)	**51.6** (159)<br>28.9 (89)
communication /discussion with patient/family	not allowed<br>under supervision<br>shared responsibility<br>full autonomy	7.6 (58)<br>19.6 (149)<br>**40.0** (304)<br>32.8 (249)	2.0 (7)<br>13.6 (47)<br>**42.5** (147)<br>41.9 (145)	0 (0)<br>12.4 (44)<br>42.3 (150)<br>45.**4** (161)	0 (0)<br>10.3 (36)<br>42.7 (150)<br>**47.0** (165)	0 (0)<br>9.5 (33)<br>43.4 (151)<br>**47.1** (164)
inter / intraprofessional referrals	not allowed<br>under supervision<br>shared responsibility<br>full autonomy	14.3 (104)<br>23.9 (174)<br>**43.5** (316)<br>18.3 (133)	4.5 (14)<br>21.0 (65)<br>**47.7** (148)<br>26.8 (83)	2.8 (9)<br>19.4 (62)<br>**48.4** (155)<br>29.4 (94)	0.6 (2)<br>16.8 (53)<br>49.8 (157)<br>32.7 (103)	0 (0)<br>16.3 (50)<br>**49.5** (152)<br>34.2 (105)
facilitation of medication management	not allowed<br>under supervision<br>shared responsibility<br>full autonomy	21.7 (159)<br>20.5 (150)<br>**36.5** (267)<br>21.3 (156)	4.7 (15)<br>24.8 (79)<br>**46.5** (148)<br>23.9 (76)	1.8 (6)<br>21.4 (70)<br>**48.6** (159)<br>28.1 (92)	0 (0)<br>18.9 (61)<br>**49.8** (161)<br>31.3 (101)	0 (0)<br>18.7 (59)<br>**47.9** (151)<br>33.3 (105)
self-care support and education of patients	not allowed<br>under supervision<br>shared responsibility<br>full autonomy	11.0 (82)<br>15.9 (118)<br>35.4 (263)<br>**37.7** (280)	1.5 (5)<br>12.8 (43)<br>42.4 (143)<br>**43.3** (146)	0.3 (1)<br>11.2 (39)<br>41.7 (145)<br>**46.8** (163)	0 (0)<br>10.2 (35)<br>39.8 (137)<br>**50.0** (172)	0 (0)<br>9.7 (33)<br>38.4 (131)<br>**51.9** (177)
advice (to patient or other healthcare professional)	not allowed<br>under supervision<br>shared responsibility<br>full autonomy	9.0 (64)<br>22.4 (160)<br>**42.4** (302)<br>26.2 (187)	4.4 (14)<br>17.8 (57)<br>**44.1** (141)<br>33.8 (108)	1.2 (4)<br>15.4 (51)<br>**47.0** (156)<br>36.4 (121)	0.3 (1)<br>13.0 (42)<br>**46.3** (150)<br>40.4 (131)	0.3 (1)<br>11.5 (37)<br>**45.2** (145)<br>43.0 (138)
transitional care communication, inter / intraprofessional collaboration / communication including reporting, advising, informing, alerting & discussing	not allowed<br>under supervision<br>shared responsibility<br>full autonomy	10.4 (75)<br>21.1 (153)<br>**44.1** (319)<br>24.4 (177)	4.6 (15)<br>18.2 (60)<br>**44.4** (146)<br>32.8 (108)	0.6 (2)<br>17.2 (58)<br>**45.1** (152)<br>37.1 (125)	0 (0)<br>13.5 (45)<br>**47.6** (159)<br>38.9 (130)	0 (0)<br>12.7 (42)<br>**44.7** (148)<br>42.6 (141)
mentoring colleagues	not allowed<br>under supervision<br>shared responsibility<br>full autonomy	7.7 (53)<br>21.5 (148)<br>**38.9** (268)<br>31.9 (220)	6.7 (21)<br>15.9 (50)<br>**43.3** (136)<br>34.1 (107)	1.8 (6)<br>16.1 (53)<br>**43.9** (145)<br>38.2 (126)	0.3 (1)<br>12.4 (40)<br>41.6 (134)<br>**45.7** (147)	0 (0)<br>11.7 (37)<br>40.4 (128)<br>**47.9** (152)
**PHARMACEUTICAL CARE DOMAIN 2: MANAGEMENT OF MEDICINES ADHERENCE (n=796)**						
**Task within ‘management of medicines adherence’**	Degree of responsibility for nurses performing this task	**All nurses** (no distinction between levels of education)	**level 5 nurses**	**level 6 nurses**	**level 7 nurses**	**level 8 nurses**
		% (n)	% (n)	% (n)	% (n)	% (n)
observation, documentation, registration, report	not allowed<br>under supervision<br>shared responsibility<br>full autonomy	4.0 (32)<br>15.1 (120)<br>35.8 (285)<br>**45.1** (359)	0.3 (1)<br>12.4 (48)<br>30.3 (117)<br>**57.0** (220)	0 (41)<br>10.5 (119)<br>30.5 (230)<br>**59.0** (390)	0.3 (1)<br>9.3 (36)<br>30.0 (116)<br>**60.5** (234)	0 (0)<br>9.3 (36)<br>29.5 (114)<br>**61.1** (236)

assessing patients competences	not allowed<br>under supervision<br>shared responsibility<br>full autonomy	4.3 (33)<br>15.6 (119)<br>38.3 (293)<br>**41.8** (320)	0.8 (3)<br>11.3 (41)<br>36.3 (132)<br>**51.6** (188)	0 (0)<br>10.5 (39)<br>37.0 (137)<br>**52.4** (194)	0.3 (1)<br>8.7 (32)<br>37.2 (137)<br>**53.8** (198)	0 (0)<br>9.0 (33)<br>35.3 (129)<br>**55.6** (203)
assessment and addressing patient/family needs	not allowed<br>under supervision<br>shared responsibility<br>full autonomy	3.0 (23)<br>13.2 (101)<br>**45.6** (349)<br>38.2 (292)	1.4 (5)<br>98 (36)<br>41.6 (153)<br>**47.3** (174)	0 (0)<br>9.7 (36)<br>41.6 (155)<br>**48.8** (182)	0.3 (1)<br>7.3 (27)<br>43.3 (161)<br>**49.2** (183)	0 (0)<br>7.0 (26)<br>41.6 (154)<br>**51.4** (190)
recognising & preventing risks / complications / medication errors	not allowed<br>under supervision<br>shared responsibility<br>full autonomy	4.8 (37)<br>15.1 (116)<br>**52.2** (401)<br>27.9 (214)	1.6 (6)<br>14.3 (53)<br>**46.2** (171)<br>37.8 (140)	0.3 (1)<br>13.2 (50)<br>**45.6** (173)<br>40.9 (155)	0.5 (2)<br>10.7 (40)<br>**47.2** (176)<br>41.6 (155)	0.3 (1)<br>10.3 (38)<br>**46.2** (171)<br>43.2 (160)
identification, reporting and addressing contra-indications, drug related problems	not allowed<br>under supervision<br>shared responsibility<br>full autonomy	8.6 (65)<br>18.5 (140)<br>**53.5** (404)<br>19.3 (146)	3.8 (13)<br>17.9 (62)<br>**50.0** (173)<br>28.3 (98)	0.8 (3)<br>17.3 (62)<br>**50.3** (180)<br>31.6 (113)	0.6 (2)<br>15.1 (53)<br>**50.1** (176)<br>34.2 (120)	0.3 (1)<br>14.3 (50)<br>**50.3** (176)<br>35.1 (123)
follow-up	not allowed<br>under supervision<br>shared responsibility<br>full autonomy	5.8 (44)<br>16.5 (125)<br>**44.4** (337)<br>33.3 (253)	2.2 (8)<br>15.3 (55)<br>39.0 (140)<br>**43.5** (156)	0.8 (3)<br>13.2 (48)<br>40.3 (147)<br>**45.8** (167)	0.3 (1)<br>11.6 (42)<br>40.5 (147)<br>**47.7** (173)	0 (0)<br>10.4 (37)<br>39.8 (142)<br>**49.9** (178)
evidence based practice	not allowed<br>under supervision<br>shared responsibility<br>full autonomy	7.7 (55)<br>16.1 (115)<br>**45.2** (323)<br>31.0 (221)	3.6 (12)<br>17.1 (57)<br>**41.6** (139)<br>37.7 (126)	2.0 (7)<br>15.0 (52)<br>**43.1** (149)<br>39.9 (38)	0.3 (1)<br>12.9 (44)<br>41.6 (142)<br>**45.2** (154)	0 (0)<br>12.1 (41)<br>40.5 (137)<br>**47.3** (160)
decision making	not allowed<br>under supervision<br>shared responsibility<br>full autonomy	15.0 (109)<br>20.3 (148)<br>**47.0** (342)<br>17.7 (129)	6.9 (22)<br>20.2 (65)<br>**48.3** (155)<br>24.6 (79)	3.0 (10)<br>21.6 (72)<br>**47.0** (157)<br>28.4 (95)	1.8 (6)<br>18.5 (61)<br>**50.0** (165)<br>29.7 (98)	0.6 (2)<br>17.5 (57)<br>**50.6** (165)<br>31.3 (102)
communication /discussion with patient/family	not allowed<br>under supervision<br>shared responsibility<br>full autonomy	5.8 (44)<br>15.2 (116)<br>**45.9** (351)<br>33.2 (254)	1.9 (7)<br>14.4 (52)<br>40.8 (147)<br>**42.8** (154)	0 (0)<br>13.4 (50)<br>41.8 (156)<br>**44.8** (167)	0.3 (1)<br>10.5 (39)<br>41.4 (153)<br>**47.8** (177)	0 (0)<br>9.6 (35)<br>41.0 (150)<br>**49.5** (181)
detection of non-adherence, drug abuse/misuse	not allowed<br>under supervision<br>shared responsibility<br>full autonomy	4.6 (35)<br>14.1 (108)<br>**46.4** (356)<br>34.9 (268)	1.6 (6)<br>13.5 (49)<br>**43.7** (159)<br>41.2 (150)	0 (0)<br>10.9 (41)<br>**47.1** (177)<br>42.0 (158)	0.3 (1)<br>9.9 (37)<br>**45.3** (169)<br>44.5 (166)	0 (0)<br>9.7 (36)<br>43.8 (163)<br>**46.5** (173)
motivational interviewing	not allowed<br>under supervision<br>shared responsibility<br>full autonomy	4.2 (31)<br>13.9 (103)<br>**42.4** (313)<br>39.5 (292)	2.5 (9)<br>13.7 (50)<br>**43.2** (158)<br>40.7 (149)	0.3 (1)<br>12.3 (47)<br>**46.2** (177)<br>41.3 (158)	0.3 (1)<br>10.0 (38)<br>44.5 (169)<br>**45.3** (172)	0 (0)<br>9.3 (35)<br>44.2 (167)<br>**46.6** (176)

Inter / intraprofessional referrals	not allowed<br>under supervision<br>shared responsibility<br>full autonomy	11.4 (82)<br>18.9 (136)<br>**49.6** (356)<br>20.1 (144)	6.5 (21)<br>20.5 (66)<br>**47.2** (152)<br>25.8 (83)	1.2 (4)<br>21.6 (72)<br>**49.7** (166)<br>27.5 (92)	0.3 (1)<br>19.3 (64)<br>**49.5** (164)<br>30.8 (102)	0 (0)<br>18.3 (60)<br>**48.5** (159)<br>33.1 (109)
facilitation of medication management	not allowed<br>under supervision<br>shared responsibility<br>full autonomy	18.2 (131)<br>16.1 (116)<br>**39.0** (281)<br>26.7 (192)	3.0 (10)<br>20.1 (66)<br>16.5 (153)<br>**30.4** (100)	1.5 (5)<br>19.0 (64)<br>**47.6** (160)<br>31.8 (107)	0.6 (2)<br>15.7 (53)<br>**49.0** (165)<br>34.7 (117)	0.3 (1)<br>15.3 (51)<br>**46.1** (154)<br>38.3 (128)
selfcare support and therapeutic education	not allowed<br>under supervision<br>shared responsibility<br>full autonomy	4.0 (30)<br>13.4 (101)<br>**41.1** (310)<br>41.6 (314)	2.2 (8)<br>12.9 (47)<br>36.9 (134)<br>**47.9** (174)	0.5 (2)<br>12.1 (45)<br>37.8 (141)<br>**49.6** (185)	0.3 (1)<br>10.2 (38)<br>37.9 (141)<br>**51.6** (192)	0 (0)<br>9.2 (34)<br>38.1 (141)<br>**52.7** (195)
advice (patient or other healthcare professional)	not allowed<br>under supervision<br>shared responsibility<br>full autonomy	7.0 (51)<br>16.6 (122)<br>**45.7** (335)<br>30.7 (225)	3.5 (12)<br>17.2 (59)<br>**40.4** (139)<br>39.0 (134)	0.8 (3)<br>15.0 (53)<br>**43.8** (155)<br>40.4 (143)	0.6 (2)<br>13.6 (48)<br>41.5 (147)<br>**44.4** (157)	0 (0)<br>12.0 (42)<br>41.4 (145)<br>**46.6** (163)
transitional care communication, inter / intraprofessional collaboration / communication including reporting, advising, informing, alerting and discussing	not allowed<br>under supervision<br>shared responsibility<br>full autonomy	7.1 (53)<br>15.9 (118)<br>**51.5** (383)<br>25.4 (189)	2.9 (10)<br>14.7 (50)<br>**48.4** (164)<br>33.9 (115)	0.9 (3)<br>15.5 (54)<br>**47.9** (167)<br>35.8 (125)	0.3 (1)<br>13.7 (48)<br>**48.1** (169)<br>37.9 (133)	0 (0)<br>12.1 (42)<br>**47.4** (165)<br>40.5 (141)
mentoring colleagues	not allowed<br>under supervision<br>shared responsibility<br>full autonomy	7.3 (52)<br>16.8 (119)<br>**44.6** (316)<br>31.2 (221)	5.8 (19)<br>17.2 (56)<br>**39.7** (129)<br>37.2 (121)	1.2 (4)<br>16.5 (55)<br>**42.9** (143)<br>39.3 (131)	0.6 (2)<br>13.6 (46)<br>41.2 (139)<br>**44.5** (150)	0 (0)<br>12.5 (42)<br>39.4 (132)<br>**48.1** (161)
**PHARMACEUTICAL CARE DOMAIN 3: MANAGEMENT OF PATIENT MEDICATION SELF-MANAGEMENT (n=726)**						
**Task within 'management of patient medication self-management'**	Degree of responsibility for nurses performing this task	**All nurses** (no distinction between levels of education)	**level 5 nurses**	**level 6 nurses**	**level 7 nurses**	**level 8 nurses**
		% (n)	% (n)	% (n)	% (n)	% (n)
observation, documentation, registration, report	not allowed<br>under supervision<br>shared responsibility<br>full autonomy	3.6 (24)<br>9.2 (62)<br>33.5 (226)<br>**53.8** (363)	0.3 (1)<br>12.3 (46)<br>32.9 (123)<br>**54.5** (204)	0 (0)<br>11.2 (42)<br>34.1 (128)<br>**54.7** (205)	0.3 (1)<br>10.8 (40)<br>33.3 (124)<br>**55.6** (207)	0 (0)<br>10.8 (40)<br>32.5 (120)<br>**56.6** (209)
assessing patients competences	not allowed<br>under supervision<br>shared responsibility<br>full autonomy	3.1 (23)<br>8.8 (66)<br>43.9 (330)<br>**44.3** (333)	0.6 (2)<br>12.1 (44)<br>37.5 (136)<br>**49.9** (181)	0 (0)<br>9.3 (34)<br>38.3 (140)<br>**52.5** (192)	0.3 (1)<br>8.8 (32)<br>37.0 (134)<br>**53.9** (195)	0 (0)<br>8.7 (31)<br>36.5 (130)<br>**54.8** (15)
assessment and addressing patient/family needs	not allowed<br>under supervision	3.1 (23)<br>7.8 (59)	0.8 (3)<br>10.3 (37)	0 (0)<br>9.2 (33)	0.3 (1)<br>8.5 (30)	0 (0)<br>8.0 (28)

	shared responsibility	**46.8** (352)	39.7 (142)	40.3 (145)	39.5 (140)	39.1 (136)
	full autonomy	42.3 (318)	**49.2** (176)	**50.6** (182)	**51.7** (183)	**52.9** (184)
recognising & preventing risks / complications / medication errors	not allowed	4.3 (32)	1.1 (4)	0.3 (1)	0.6 (2)	0 (0)
	under supervision	10.9 (81)	15.2 (55)	12.4 (45)	11.2 (40)	11.4 (40)
	shared responsibility	**54.2** (404)	**44.2** (160)	**45.2** (164)	**45.3** (162)	**44.9** (158)
	full autonomy	30.7 (229)	39.5 (143)	42.1 (153)	43.0 (154)	43.8 (154)
identification, reporting and addressing contra-indications, drug related problems	not allowed	7.3 (54)	1.8 (6)	0 (0)	0.6 (2)	0.3 (1)
	under supervision	12.4 (92)	15.5 (53)	14.4 (49)	12.1 (41)	12.3 (41)
	shared responsibility	**56.1** (417)	**49.3** (168)	**50.9** (173)	**51.2** (173)	**49.4** (164)
	full autonomy	24.2 (180)	33.4 (114)	34.7 (118)	36.1 (122)	38.0 (126)
follow-up	not allowed	5.6 (42)	2.6 (9)	0.3 (1)	0.6 (2)	0.3 (1)
	under supervision	10.8 (80)	12.8 (45)	11.6 (41)	10.3 (36)	10.5 (36)
	shared responsibility	**49.2** (366)	41.2 (145)	43.5 (153)	43.4 (151)	42.3 (145)
	full autonomy	34.4 (256)	**43.5** (153)	**44.6** (157)	**45.7** (159)	**46.9** (161)
evidence based practice	not allowed	7.5 (53)	3.1 (10)	0.9 (3)	0.3 (1)	0 (0)
	under supervision	11.7 (83)	18.2 (59)	14.4 (47)	13.8 (45)	12.8 (41)
	shared responsibility	**46.3** (329)	35.5 (115)	39.1 (128)	36.7 (120)	35.9 (115)
	full autonomy	34.5 (245)	**43.2** (140)	**45.6** (149)	**49.2** (164)	**51.2** (164)
decision making	not allowed	14.2 (102)	2.3 (7)	1.0 (3)	0.3 (1)	0 (0)
	under supervision	14.9 (107)	21.4 (66)	18.8 (59)	17.9 (56)	17.0 (52)
	shared responsibility	**49.4** (356)	**44.7** (138)	**47.3** (148)	**46.0** (144)	**45.9** (140)
	full autonomy	21.5 (155)	31.7 (98)	32.9 (103)	35.8 (112)	37.0 (113)
communication /discussion with patient/family	not allowed	5.1 (39)	0.8 (3)	0 (0)	0.6 (2)	0 (0)
	under supervision	8.4 (64)	11.4 (41)	10.0 (36)	9.7 (35)	9.4 (33)
	shared responsibility	**50.7** (386)	43.7 (157)	44.9 (162)	43.5 (157)	42.5 (149)
	full autonomy	35.7 (272)	**44.0** (158)	**45.2** (163)	**46.3** (167)	**48.1** (169)
motivational interviewing	not allowed	5.5 (41)	1.2 (4)	0.6 (2)	0.6 (2)	0.3 (1)
	under supervision	8.2 (61)	12.8 (44)	10.6 (37)	8.6 (30)	9.1 (31)
	shared responsibility	**43.8** (327)	33.9 (117)	35.7 (125)	34.7 (121)	33.8 (115)
	full autonomy	42.6 (318)	**52.2** (180)	**53.1** (186)	**56.2** (196)	**56.8** (193)
Inter / intraprofessional referrals	not allowed	12.1 (86)	3.3 (10)	1.6 (5)	1.0 (3)	0 (0)
	under supervision	12.6 (90)	20.8 (64)	18.6 (58)	16.3 (51)	16.6 (51)
	shared responsibility	**51.8** (369)	**43.0** (132)	**44.4** (138)	**44.9** (140)	**44.0** (135)
	full autonomy	23.6 (168)	32.9 (101)	35.4 (110)	37.8 (118)	39.4 (121)
facilitation of medication management	not allowed	13.4 (95)	2.7 (8)	1.0 (3)	0.3 (1)	0 (0)
	under supervision	13.4 (95)	17.0 (51)	14.5 (44)	13.6 (41)	14.4 (43)
	shared responsibility	**44.9** (318)	**47.0** (141)	**50.3** (153)	**48.3** (146)	**47.5** (142)
	full autonomy	28.3 (201)	33.0 (100)	34.2 (104)	37.7 (114)	38.1 (114)
selfcare support and therapeutic education	not allowed	4.8 (36)	1.4 (5)	0.3 (1)	0.3 (1)	0 (0)
	under supervision	9.3 (70)	12.9 (46)	10.1 (36)	9.4 (33)	9.8 (34)

	shared responsibility	**46.9** (352)	40.2 (143)	42.5 (152)	40.6 (143)	40.3 (140)
	full autonomy	38.9 (292)	**45.5** (162)	**47.2** (169)	**49.7** (175)	**49.9** (173)
advice (patient or other healthcare professional)	not allowed	9.0 (64)	1.3 (4)	0.3 (1)	0.3 (1)	0 (0)
	under supervision	11.5 (82)	17.9 (57)	13.1 (42)	12.3 (39)	12.4 (39)
	shared responsibility	**49.0** (350)	**41.7** (133)	**47.7** (153)	**44.0** (140)	**46.6** (136)
	full autonomy	30.6 (219)	39.2 (125)	38.9 (125)	43.4 (138)	44.3 (139)
transitional care communication, inter / intraprofessional collaboration / communication including reporting, advising, informing, alerting and discussing	not allowed	7.2 (53)	1.8 (6)	0.3 (1)	0.3 (1)	0 (0)
	under supervision	11.9 (88)	17.0 (57)	14.5 (49)	12.5 (42)	12.6 (42)
	shared responsibility	**55.4** (410)	**46.1** (155)	**47.8** (161)	**47.6** (160)	**45.9** (153)
	full autonomy	25.5 (189)	35.1 (118)	37.4 (126)	39.6 (133)	41.4 (138)
mentoring colleagues	not allowed	8.0 (56)	4.1 (13)	0.6 (2)	0.6 (2)	0.3 (1)
	under supervision	10.3 (72)	15.6 (50)	13.0 (42)	10.9 (35)	11.1 (35)
	shared responsibility	**45.7** (319)	37.5 (120)	39.2 (127)	36.9 (118)	35.1 (111)
	full autonomy	36.0 (251)	**42.8** (137)	**47.2** (153)	**51.6** (165)	**53.5** (169)
**PHARMACEUTICAL CARE DOMAIN 4: MANAGEMENT OF PATIENT EDUCATION AND INFORMATION (n=731)**						
**Task within 'management of patient education and information**	Degree of responsibility for nurses performing this task	**All nurses** (no distinction between levels of education) % (n)	**level 5 nurses** % (n)	**level 6 nurses** % (n)	**level 7 nurses** % (n)	**level 8 nurses** % (n)
observation, documentation, registration, report	not allowed	4.2 (32)	1.1 (4)	0 (0)	0 (0)	0 (0)
	under supervision	9.8 (75)	11.0 (39)	9.8 (35)	9.1 (32)	9.1 (32)
	shared responsibility	37.8 (289)	28.8 (102)	29.3 (105)	28.8 (101)	27.8 (98)
	full autonomy	**48.2** (369)	**59.0** (209)	**60.9** (218)	**62.1** (218)	**63.1** (222)
assessing patients competences	not allowed	4.8 (36)	0.6 (2)	0 (0)	0 (0)	0 (0)
	under supervision	9.0 (68)	9.3 (32)	7.2 (25)	7.0 (24)	6.7 (23)
	shared responsibility	42.4 (320)	38.0 (131)	38.7 (135)	36.6 (126)	36.2 (124)
	full autonomy	**43.8** (331)	**52.2** (180)	**54.2** (189)	**56.4** (194)	**57.1** (196)
assessment and addressing patient/family needs	not allowed	3.7 (28)	0.6 (2)	0 (0)	0 (0)	0 (0)
	under supervision	7.8 (59)	10.3 (36)	9.3 (33)	8.9 (31)	8.1 (28)
	shared responsibility	**44.9** (338)	39.7 (138)	40.2 (142)	38.7 (135)	38.6 (134)
	full autonomy	43.5 (327)	**49.4** (172)	**50.4** (178)	**52.4** (183)	**53.3** (185)
follow-up	not allowed	5.7 (43)	2.1 (7)	0 (0)	0 (0)	0 (0)
	under supervision	9.4 (71)	11.9 (40)	11.4 (39)	9.9 (33)	8.7 (29)
	shared responsibility	**46.7** (351)	37.7 (127)	38.7 (132)	38.2 (128)	38.3 (128)
	full autonomy	38.2 (287)	**48.4** (16)	**49.9** (170)	**51.9** (174)	**53.0** (177)
evidence based practice	not allowed	7.7 (55)	4.1 (13)	0.6 (2)	0 (0)	0 (0)
	under supervision	12.0 (85)	15.7 (50)	13.7 (45)	13.5 (43)	11.3 (36)
	shared responsibility	**47.5** (338)	**40.3** (128)	41.8 (137)	39.2 (125)	39.6 (126)
	full autonomy	32.8 (233)	39.9 (127)	**43.9** (144)	**47.3** (151)	**49.1** (156)

decision making	not allowed<br>under supervision<br>shared responsibility<br>full autonomy	12.8 (93)<br>15.0 (109)<br>**49.4** (358)<br>22.8 (165)	3.3 (10)<br>21.1 (64)<br>**46.1** (140)<br>29.6 (90)	0 (0)<br>19.6 (61)<br>**48.1** (150)<br>32.4 (101)	0 (0)<br>16.7 (51)<br>**48.2** (147)<br>35.1 (107)	0 (0)<br>15.8 (48)<br>**47.2** (143)<br>37.0 (112)
communication /discussion with patient/family	not allowed<br>under supervision<br>shared responsibility<br>full autonomy	5.7 (43)<br>8.7 (66)<br>**51.1** (387)<br>34.5 (261)	0.9 (3)<br>12.0 (41)<br>42.6 (146)<br>**44.6** (153)	0 (0)<br>10.5 (37)<br>42.2 (149)<br>**47.3** (167)	0 (0)<br>8.7 (30)<br>42.9 (147)<br>**48.4** (166)	0 (0)<br>8.5 (29)<br>42.0 (144)<br>**49.6** (170)
motivational interviewing	not allowed<br>under supervision<br>shared responsibility<br>full autonomy	5.9 (44)<br>7.6 (56)<br>43.2 (320)<br>**43.3** (321)	1.5 (5)<br>10.3 (35)<br>36.6 (124)<br>**51.6** (175)	0.3 (1)<br>8.3 (29)<br>37.6 (131)<br>**53.7** (187)	0 (0)<br>7.0 (24)<br>37.2 (127)<br>**55.7** (190)	0 (0)<br>5.9 (20)<br>35.6 (120)<br>**58.5** (197)
Inter / intraprofessional referrals	not allowed<br>under supervision<br>shared responsibility<br>full autonomy	13.1 (94)<br>13.1 (94)<br>**51.7** (371)<br>22.1 (159)	4.1 (12)<br>17.6 (52)<br>**46.8** (138)<br>31.5 (93)	0.7 (2)<br>17.8 (53)<br>**48.0** (143)<br>33.6 (100)	0 (0)<br>14.9 (44)<br>**49.5** (146)<br>35.6 (105)	0 (0)<br>15.0 (44)<br>**47.3** (139)<br>37.8 (111)
facilitation of medication management	not allowed<br>under supervision<br>shared responsibility<br>full autonomy	14.9 (105)<br>14.3 (101)<br>**47.8** (337)<br>23.0 (162)	2.4 (7)<br>21.5 (63)<br>**45.7** (134)<br>30.4 (89)	1.3 (4)<br>19.1 (57)<br>**48.3** (144)<br>31.2 (93)	0.3 (1)<br>17.3 (51)<br>**46.9** (138)<br>35.4 (104)	0.3 (1)<br>17.4 (51)<br>**46.4** (136)<br>35.8 (105)
selfcare support and therapeutic education	not allowed<br>under supervision<br>shared responsibility<br>full autonomy	4.4 (33)<br>7.6 (57)<br>**48.1** (359)<br>39.8 (297)	1.1 (4)<br>11.7 (41)<br>38.7 (135)<br>**48.4** (169)	0.6 (2)<br>9.9 (35)<br>39.2 (139)<br>**50.4** (179)	0 (0)<br>9.1 (32)<br>38.5 (135)<br>**52.4** (184)	0 (0)<br>8.9 (31)<br>38.1 (133)<br>**53.0** (185)
advice (patient or other healthcare professional)	not allowed<br>under supervision<br>shared responsibility<br>full autonomy	8.1 (59)<br>10.8 (78)<br>**49.7** (360)<br>31.4 (228)	3.4 (11)<br>15.8 (51)<br>**41.9** (135)<br>38.8 (125)	0.3 (1)<br>13.5 (44)<br>**44.8** (146)<br>41.4 (135)	0 (0)<br>12.7 (41)<br>41.6 (134)<br>**45.7** (147)	0 (0)<br>12.1 (39)<br>40.2 (129)<br>**47.7** (153)
transitional care communication, inter / intraprofessional collaboration / communication including reporting, advising, informing, alerting and discussing	not allowed<br>under supervision<br>shared responsibility<br>full autonomy	8.7 (63)<br>10.6 (77)<br>**55.7** (405)<br>25.0 (182)	2.2 (7)<br>19.4 (62)<br>**42.9** (137)<br>35.4 (113)	0.3 (1)<br>15.8 (51)<br>**45.7** (147)<br>38.2 (123)	0 (0)<br>14.8 (47)<br>**44.0** (140)<br>41.2 (131)	0 (0)<br>13.6 (43)<br>**43.8** (139)<br>42.6 (135)
mentoring colleagues	not allowed<br>under supervision<br>shared responsibility<br>full autonomy	7.0 (49)<br>11.8 (82)<br>**47.2** (329)<br>34.0 (237)	4.8 (15)<br>16.3 (51)<br>**39.6** (124)<br>39.3 (123)	0.6 (2)<br>15.0 (48)<br>40.3 (129)<br>**44.1** (141)	0 (0)<br>13.1 (41)<br>38.2 (120)<br>**48.7** (153)	0 (0)<br>11.9 (37)<br>36.9 (115)<br>**51.3** (160)
**RESPONSIBILITY 5: PRESCRIPTION MANAGEMENT (n=669)**						
**Task within ‘prescription management’**	Degree of responsibility for	**All nurses** (no distinction between levels of education)	**level 5 nurses**	**level 6 nurses**	**level 7 nurses**	**level 8 nurses**

	nurses performing this task	% (n)	% (n)	% (n)	% (n)	% (n)
observation, documentation, registration, report	not allowed	31.0 (215)	5.0 (14)	1.4 (4)	0.7 (2)	0..4 (1)
	under supervision	11.0 (76)	18.9 (53)	19.3 (55)	17.1 (49)	15.4 (43)
	shared responsibility	27.1 (188)	30.7 (85)	34.0 (97)	34.3 (98)	34.4 (96)
	full autonomy	**30.9** (214)	**45.4** (127)	**45.3** (129)	**47.9** (137)	**49.8** (139)
assessing patients competences	not allowed	28.8 (192)	4.0 (11)	1.8 (5)	0.4 (1)	0.4 (1)
	under supervision	8.7 (58)	15.2 (42)	15.1 (43)	12.1 (34)	11.2 (31)
	shared responsibility	**32.3** (215)	38.3 (106)	39.4 (112)	40.1 (113)	39.9 (111)
	full autonomy	30.2 (201)	**42.6** (118)	**43.7** (124)	**47.5** (134)	**48.6** (135)
assessment and addressing patient/family needs	not allowed	18.9 (126)	2.5 (7)	2.1 (6)	0.4 (1)	0.4 (1)
	under supervision	16.8 (112)	15.7 (44)	13.8 (40)	10.9 (31)	10.6 (30)
	shared responsibility	**35.2** (235)	**44.1** (124)	**46.0** (133)	**47.0** (134)	**45.4** (128)
	full autonomy	29.1 (194)	37.7 (106)	38.1 (110)	41.8 (119)	43.6 (123)
recognising & preventing risks / complications / medication errors	not allowed	21.9 (151)	4.2 (12)	2.1 (6)	0 (0)	0 (0)
	under supervision	18.1 (125)	20.9 (60)	17.2 (50)	14.8 (43)	13.4 (38)
	shared responsibility	**38.4** (265)	**42.9** (123)	**47.2** (137)	**47.9** (139)	**48.6** (138)
	full autonomy	21.6 (149)	32.1 (92)	33.4 (97)	37.2 (108)	38.0 (108)
identification, reporting and addressing contra-indications, drug related problems	not allowed	21.6 (148)	4.0 (11)	2.1 (6)	0.4 (1)	0 (0)
	under supervision	20.6 (141)	21.6 (59)	19.6 (55)	17.0 (47)	16.2 (44)
	shared responsibility	**40.9** (280)	**48.0** (131)	**51.1** (143)	**51.1** (141)	**50.7** (138)
	full autonomy	16.8 (115)	26.4 (72)	27.1 (76)	31.5 (87)	33.1 (90)
follow-up	not allowed	20.5 (141)	4.0 (11)	1.4 (4)	0 (0)	0 (0)
	under supervision	19.6 (135)	18.7 (51)	17.6 (49)	14.3 (40)	13.5 (37)
	shared responsibility	**35.3** (243)	**40.7** (111)	**43.9** (122)	**46.2** (129)	**44.2** (121)
	full autonomy	24.6 (169)	36.6 (100)	37.1 (103)	39.4 (110)	42.3 (116)
evidence based practice	not allowed	25.2 (164)	6.4 (16)	2.3 (6)	0 (0)	0 (0)
	under supervision	17.5 (114)	16.9 (42)	15.9 (41)	12.7 (33)	10.8 (27)
	shared responsibility	**33.3** (217)	**39.8** (99)	**43.8** (113)	43.2 (112)	43.0 (108)
	full autonomy	24.0 (156)	36.9 (92)	38.0 (98)	**44.0** (114)	**46.2** (116)
decision making	not allowed	35.1 (233)	10.0 (23)	3.4 (8)	0.8 (2)	0.4 (1)
	under supervision	20.7 (137)	25.1 (58)	26.7 (63)	23.0 (55)	21.1 (49)
	shared responsibility	**32.0** (212)	**45.0** (104)	**50.4** (119)	**51.9** (124)	**52.6** (122)
	full autonomy	12.2 (81)	19.9 (46)	19.5 (46)	24.3 (58)	25.9 (60)
communication /discussion with patient/family	not allowed	20.0 (136)	4.6 (13)	1.4 (4)	0 (0)	0 (0)
	under supervision	16.6 (113)	13.2 (37)	11.5 (33)	9.8 (28)	8.5 (24)
	shared responsibility	**36.3** (247)	**45.2** (127)	**49.5** (142)	**48.1** (138)	**48.2** (136)
	full autonomy	27.2 (185)	37.0 (104)	37.6 (108)	42.2 (121)	43.3 (122)
Inter / intraprofessional referrals	not allowed	29.7 (191)	6.9 (16)	4.1 (10)	0.4 (1)	0 (0)
	under supervision	16.5 (106)	19.7 (46)	18.7 (45)	17.0 (41)	14.4 (34)

	shared responsibility	**36.0** (232)	**45.1** (105)	**48.1** (116)	**47.3** (114)	**48.3** (114)
	full autonomy	17.9 (115)	28.3 (66)	29.0 (70)	35.3 (85)	37.3 (88)
facilitation of medication management	not allowed	31.5 (210)	6.3 (16)	1.9 (5)	0.4 (1)	0 (0)
	under supervision	17.5 (117)	26.7 (68)	25.3 (65)	20.1 (51)	20.2 (50)
	shared responsibility	**31.8** (212)	**43.1** (110)	**45.5** (117)	**48.8** (124)	**47.4** (117)
	full autonomy	19.2 (128)	23.9 (61)	27.2 (70)	30.7 (78)	32.4 (80)
advice (patient or other healthcare professional)	not allowed	27.7 (184)	8.6 (22)	3.4 (9)	0 (0)	0 (0)
	under supervision	18.8 (125)	23.7 (61)	23.3 (62)	19.4 (51)	18.5 (48)
	shared responsibility	**36.3** (241)	**43.2** (111)	**47.4** (126)	**49.8** (131)	**50.0** (130)
	full autonomy	17.2 (114)	24.5 (63)	25.9 (69)	30.8 (81)	31.5 (82)
determination of type/dosage	not allowed	**52.4** (356)	16.7 (31)	9.8 (19)	3.6 (7)	1.1 (2)
	under supervision	17.2 (117)	**32.3** (60)	**34.2** (66)	30.6 (59)	32.8 (62)
	shared responsibility	19.3 (131)	29.0 (54)	32.6 (63)	**37.3** (72)	**35.4** (67)
	full autonomy	11.2 (76)	22.0 (41)	23.3 (45)	28.5 (55)	30.7 (58)
initiation of medication (reactive / proactive)	not allowed	**50.5** (344)	17.9 (36)	8.7 (18)	3.9 (8)	2.0 (4)
	under supervision	18.2 (124)	**34.8** (70)	**35.4** (73)	32.9 (98)	33.5 (68)
	shared responsibility	20.4 (139)	25.4 (51)	33.5 (69)	**34.3** (71)	**34.0** (69)
	full autonomy	10.9 (74)	21.9 (44)	22.3 (46)	29.0 (60)	30.5 (62)
adaptation of dose, dose titration	not allowed	**51.6** (349)	19.8 (38)	10.9 (22)	4.0 (8)	2.5 (5)
	under supervision	18.0 (122)	**33.9** (65)	**37.8** (76)	34.2 (68)	32.7 (65)
	shared responsibility	19.8 (134)	25.5 (49)	28.4 (57)	**34.7** (69)	**35.2** (70)
	full autonomy	10.5 (71)	20.8 (40)	22.9 (46)	27.1 (54)	29.6 (59)
decision on continuation /cessation of medication	not allowed	**51.3** (344)	15.6 (30)	9.9 (20)	3.0 (6)	1.5 (3)
	under supervision	15.6 (105)	30.7 (59)	32.7 (66)	28.5 (57)	26.9 (53)
	shared responsibility	22.7 (152)	**33.9** (65)	**36.1** (73)	**43.0** (86)	**43.7** (86)
	full autonomy	10.4 (70)	19.8 (38)	21.3 (43)	25.5 (51)	27.9 (55)
PRN (pro re nata, 'if needed' medication) / standing prescription renewal order	not allowed	40.3 (273)	10.7 (24)	4.7 (11)	0.9 (2)	1.3(3)
	under supervision	16.7 (113)	30.2 (68)	29.7 (69)	26.0 (60)	23.1(53)
	shared responsibility	**26.1** (177)	**32.4** (73)	**32.8** (76)	35.5 (82)	26.7(84)
	full autonomy	16.8 (114)	26.7 (60)	**32.8** (76)	**37.7** (87)	**28.9** (89)
medication reconciliation	not allowed	37.5 (252)	8.6 (20)	4.2 (10)	0.8 (2)	0.9 (2)
	under supervision	15.3 (103)	28.8 (37)	24.9 (59)	22.2 (53)	19.3 (45)
	shared responsibility	**31.8** (214)	**39.5** (92)	**45.1** (107)	**44.8** (107)	**45.5** (106)
	full autonomy	15.3 (103)	23.2 (54)	25.7 (61)	32.2 (77)	34.3 (80)
medication review	not allowed	41.7 (285)	10.8 (25)	6.4 (15)	1.7 (4)	1.3 (3)
	under supervision	17.7 (121)	29.9 (69)	28.6 (67)	27.9 (65)	24.7 (57)
	shared responsibility	**27.8** (190)	**35.9** (83)	**39.3** (92)	**39.9** (93)	**41.6** (96)
	full autonomy	12.9 (88)	23.4 (54)	25.6 (60)	30.5 (71)	32.5 (75)
intervention in case of emergency	not allowed	29.0 (200)	6.4 (18)	2.8 (8)	0 (0)	0.4 (1)
	under supervision	17.6 (121)	26.1 (73)	23.4 (67)	21.9 62)	21.0 (59)

	shared responsibility	**34.4** (237)	**41.8** (117)	**46.2** (132)	**46.3** (131)	**45.9** (129)
	full autonomy	19.0 (131)	25.7 (72)	27.6 (79)	31.8 (90)	32.7 (92)
transitional care communication, inter / intraprofessional collaboration / communication including reporting, advising, informing, alerting and discussing	not allowed	26.7 (177)	5.3 (14)	3.0 (8)	0 (0)	0 (0)
	under supervision	12.2 (81)	15.6 (41)	16.6 (41)	12.6 (34)	11.9 (32)
	shared responsibility	**42.0** (279)	**51.0** (134)	**51.7** (116)	**54.1** (146)	**54.1** (146)
	full autonomy	19.1 (127)	28.1 (74)	28.8 (93)	33.3 (90)	34.1 (92)
mentoring colleagues	not allowed	29.0 (193)	8.3 (21)	4.6 (2)	0.4 (1)	0 (0)
	under supervision	11.4 (76)	16.7 (42)	15.6 (49)	14.7 (38)	12.2 (31)
	shared responsibility	**33.4** (222)	**42.1** (106)	**44.3** (98)	**44.2** (114)	**44.1** (112)
	full autonomy	26.2 (174)	32.9 (83)	35.5 (137)	40.7 (105)	43.7 (111)

PHARMACEUTICAL CARE DOMAIN 6: MEDICATION SAFETY MANAGEMENT (n=738)

**Task within 'medication safety management'**	Degree of responsibility for nurses performing this task	**All nurses** (no distinction between levels of education)	**level 5 nurses**	**level 6 nurses**	**level 7 nurses**	**level 8 nurses**
		% (n)	% (n)	% (n)	% (n)	% (n)
observation, documentation, registration, report	not allowed	10.1 (79)	0.8 (3)	0.3 (1)	0 (0)	0 (0)
	under supervision	13.5 (105)	11.5 (42)	9.7 (36)	10.2 (37)	9.7 (35)
	shared responsibility	35.8 (279)	35.6 (130)	36.4 (135)	35.5 (128)	35.0 (126)
	full autonomy	**40.6** (316)	**52.1** (190)	**53.6** (199)	**54.3** (196)	**55.3** (199)
assessing patients competences	not allowed	10.7 (81)	0.6 (2)	0 (0)	0 (0)	0 (0)
	under supervision	13.9 (105)	12.5 (44)	9.9 (35)	10.3 (36)	9.6 (33)
	shared responsibility	**38.8** (293)	40.2 (141)	41.9 (148)	41.1 (143)	40.6 (140)
	full autonomy	36.6 (277)	**46.7** (164)	**48.2** (170)	**48.6** (169)	**49.9** (172)
assessment and addressing patient/family needs	not allowed	4.6 (35)	0.3 (1)	0 (0)	0 (0)	0 (0)
	under supervision	14.7 (111)	10.0 (35)	8.1 (29)	7.4 (26)	6.6 (23)
	shared responsibility	**44.2** (334)	44.0 (154)	44.7 (159)	44.6 (156)	44.2 (153)
	full autonomy	36.5 (276)	**45.7** (160)	**47.2** (168)	**48.0** (168)	**49.1** (170)
recognising & preventing risks / complications / medication errors	not allowed	5.9 (45)	1.1 (4)	0.5 (2)	0.6 (2)	0.3 (1)
	under supervision	17.0 (129)	13.3 (47)	11.8 (43)	10.1 (36)	9.7 (34)
	shared responsibility	**48.9** (372)	**45.8** (162)	**47.4** (173)	**48.2** (171)	**47.6** (167)
	full autonomy	28.3 (215)	39.8 (141)	40.3 (147)	41.1 (146)	42.5 (149)
identification, reporting and addressing contra-indications, drug related problems	not allowed	7.8 (58)	1.5 (5)	0.9 (3)	0.3 (1)	0.3 (1)
	under supervision	18.6 (139)	17.1 (58)	14.3 (50)	12.6 (43)	12.1 (41)
	shared responsibility	**50.1** (374)	**48.2** (164)	**50.1** (175)	**51.6** (176)	**51.2** (173)
	full autonomy	23.6 (176)	33.2 (113)	34.7 (121)	35.5 (121)	36.4 (123)
follow-up	not allowed	6.1 (46)	1.4 (5)	0.3 (1)	0 (0)	0 (0)
	under supervision	17.2 (129)	13.1 (46)	10.8 (38)	9.8 (34)	9.3 (32)
	shared responsibility	**47.7** (359)	**46.0** (161)	**48.7** (172)	**48.4** (168)	**47.2** (163)
	full autonomy	29.0 (218)	39.4 (138)	40.2 (142)	41.8 (145)	43.5 (150)

evidence based practice	not allowed<br>under supervision<br>shared responsibility<br>full autonomy	8.9 (64)<br>20.0 (144)<br>**41.7** (301)<br>29.4 (212)	3.4 (11)<br>16.4 (53)<br>**40.1** (130)<br>40.0 (130)	2.7 (9)<br>14.8 (49)<br>**42.0** (139)<br>40.5 (134)	0 (0)<br>13.8 (45)<br>41.7 (136)<br>**44.5** (145)	0 (0)<br>12.8 (41)<br>40.5 (13)<br>**46.7** (150)
decision making	not allowed<br>under supervision<br>shared responsibility<br>full autonomy	16.5 (120)<br>23.8 (173)<br>**44.4** (323)<br>15.3 (111)	3.7 (11)<br>24.4 (72)<br>**49.8** (147)<br>22.0 (65)	1.3 (4)<br>24.2 (73)<br>**52.6** (159)<br>21.9 (66)	0.7 (2)<br>20.3 (61)<br>**54.5** (164)<br>24.6 (74)	0.3 (1)<br>19.5 (58)<br>**52.2** (155)<br>27.9 (83)
communication /discussion with patient/family	not allowed<br>under supervision<br>shared responsibility<br>full autonomy	5.6 (42)<br>15.9 (119)<br>**47.3** (354)<br>31.2 (234)	1.2 (4)<br>13.6 (47)<br>**46.5** (161)<br>38.7 134)	0 (0)<br>12.2 (43)<br>**49.6** (175)<br>38.2 (135)	0 (0)<br>10.1 (35)<br>**48.4** (167)<br>41.4 (143)	0 (0)<br>10.2 (35)<br>**46.4** (159)<br>43.4 (149)
Inter / intraprofessional referrals	not allowed<br>under supervision<br>shared responsibility<br>full autonomy	13.0 (94)<br>19.1 (138)<br>**48.8** (352)<br>19.0 (137)	4.0 (12)<br>16.1 (48)<br>**53.8** (161)<br>26.1 (78)	0.7 (2)<br>15.6 (47)<br>**57.6** (174)<br>26.2 (79)	0 (0)<br>14.3 (43)<br>**55.7** (167)<br>30.0 (90)	0 (0)<br>13.5 (40)<br>**54.4** (161)<br>32.1 (95)
facilitation of medication management	not allowed<br>under supervision<br>shared responsibility<br>full autonomy	15.5 (112)<br>18.4 (133)<br>**42.8** (309)<br>23.3 (168)	3.5 (11)<br>18.9 (59)<br>**47.8** (149)<br>29.8 (93)	0.6 (2)<br>17.9 (57)<br>**51.9** (165)<br>29.6 (94)	0 (0)<br>16.3 (51)<br>**51.4** (161)<br>32.3 (101)	0 (0)<br>15.9 (49)<br>**49.8** (154)<br>34.3 (106)
selfcare support and therapeutic education	not allowed<br>under supervision<br>shared responsibility<br>full autonomy	5.7 (43)<br>14.6 (109)<br>**45.4** (340)<br>34.3 (257)	2.0 (7)<br>10.9 (38)<br>**43.9** (152)<br>43.6 (152)	0.6 (2)<br>10.7 (38)<br>44.2 (157)<br>**44.5** (158)	0 (0)<br>8.6 (30)<br>44.3 (154)<br>**47.1** (164)	0 (0)<br>8.1 (28)<br>43.2 (149)<br>**48.7** (168)
advice (patient or other healthcare professional)	not allowed<br>under supervision<br>shared responsibility<br>full autonomy	8.7 (63)<br>18.3 (133)<br>**46.0** (334)<br>27.0 (196)	1.8 (6)<br>17.1 (56)<br>**44.6** (146)<br>36.4 (119)	0 (0)<br>14.6 (49)<br>**47.5** (159)<br>37.9 (127)	0 (0)<br>13.1 (43)<br>**45.9** (151)<br>41.0 (135)	0 (0)<br>13.0 (42)<br>**43.5** (141)<br>**43.5** (141)
intervention in case of emergency	not allowed<br>under supervision<br>shared responsibility<br>full autonomy	6.5 (49)<br>21.8 (164)<br>**48.1** (361)<br>23.6 (177)	3.2 (11)<br>22.5 (78)<br>**43.9** (152)<br>30.3 (105)	0.6 (2)<br>22.5 (79)<br>**45.9** (161)<br>31.1 (109)	0 (0)<br>21.0 (73)<br>**46.1** (160)<br>32.9 (114)	0 (0)<br>19.9 (68)<br>**45.5** (155)<br>34.6 (118)
transitional care communication, inter / intraprofessional collaboration / communication (including reporting, advising, informing, alerting and discussing	not allowed<br>under supervision<br>shared responsibility<br>full autonomy	8.0 (58)<br>16.3 (118)<br>**54.1** (393)<br>21.6 (157)	2.2 (7)<br>14.2 (46)<br>**51.1** (165)<br>32.5 (105)	0.3 (1)<br>13.4 (44)<br>**52.9** (174)<br>33.4 (110)	0.3 (1)<br>11.5 (38)<br>**52.7** (174)<br>35.5 (117)	0.3 (1)<br>11.1 (36)<br>**51.1** (166)<br>37.5 (122)
mentoring colleagues	not allowed<br>under supervision<br>shared responsibility<br>full autonomy	7.9 (57)<br>16.3 (117)<br>**44.5** (319)<br>31.2 (224)	3.7 (12)<br>16.4 (53)<br>**43.2** (140)<br>36.7 (119)	0.9 (3)<br>13.5 (44)<br>**46.2** (150)<br>39.4 (128)	0.3 (1)<br>10.6 (34)<br>**46.0** (148)<br>43.2 (139)	0.3 (1)<br>10.3 (33)<br>43.6 (139)<br>**45.8** (146)

PHARMACEUTICAL CARE DOMAIN 7: (TRANSITION OF) CARE COORDINATION (n=711)						
**Task within ‘(transition of) care coordination’**	Degree of responsibility for nurses performing this task	**All nurses** (no distinction between levels of education)	**level 5 nurses**	**level 6 nurses**	**level 7 nurses**	**level 8 nurses**
		% (n)	% (n)	% (n)	% (n)	% (n)
observation, documentation, registration, report	not allowed<br>under supervision<br>shared responsibility<br>full autonomy	11.0 (82)<br>9.4 (70)<br>39.2 (293)<br>**40.5** (303)	0.9 (3)<br>13.3 (45)<br>32.2 (109)<br>**53.7** (182)	0.6 (2)<br>11.6 (40)<br>33.9 (117)<br>**53.9** (186)	0 (0)<br>10.9 (37)<br>34.6 (118)<br>**54.5** (186)	0.3 (1)<br>9.8 (33)<br>33.8 (114)<br>**56.1** (189)
assessing patients competences	not allowed<br>under supervision<br>shared responsibility<br>full autonomy	10.9 (80)<br>9.3 (68)<br>**43.4** (319)<br>36.5 (268)	0.3 (1)<br>11.7 (39)<br>39.5 (132)<br>**48.5** (162)	0.9 (3)<br>9.8 (33)<br>40.8 (137)<br>**48.5** (163)	0.3 (1)<br>9.7 (32)<br>39.6 (131)<br>**50.5** (167)	0.6 (2)<br>8.8 (29)<br>39.3 (129)<br>**51.2** (168)
assessment and addressing patient/family needs	not allowed<br>under supervision<br>shared responsibility<br>full autonomy	5.2 (38)<br>8.7 (64)<br>**47.3** (347)<br>38.8 (285)	0.3 (1)<br>11.1 (37)<br>41.4 (138)<br>**47.1** (157)	0.9 (3)<br>9.5 (32)<br>42.1 (142)<br>**47.5** (160)	0.3 (1)<br>8.2 (27)<br>42.0 (139)<br>**49.5** (164)	0.6 (2)<br>7.9 (26)<br>40.9 (134)<br>**50.6** (166)
recognising & preventing risks / complications / medication errors	not allowed<br>under supervision<br>shared responsibility<br>full autonomy	6.8 (49)<br>12.3 (89)<br>**51.0** (369)<br>29.9 (216)	1.9 (6)<br>13.8 (44)<br>41.7 (133)<br>**42.6** (136)	0.6 (2)<br>12.3 (40)<br>**44.8** (145)<br>42.3 (137)	0.3 (1)<br>11.6 (37)<br>**44.8** (143)<br>43.3 (138)	0.6 (2)<br>10.5 (33)<br>**44.4** (140)<br>**44.4** (140)
identification, reporting and addressing contra-indications, drug related problems	not allowed<br>under supervision<br>shared responsibility<br>full autonomy	9.1 (65)<br>14.1 (101)<br>**51.7** (371)<br>25.1 (180)	1.9 (6)<br>17.0 (53)<br>**41.8** (130)<br>39.2 (122)	0.9 (3)<br>14.6 (46)<br>**45.6** (144)<br>38.9 (123)	0.3 (1)<br>12.3 (38)<br>**46.1** (143)<br>41.3 (128)	0.7 (2)<br>11.4 (35)<br>**45.0** (138)<br>43.0 (132)
follow-up	not allowed<br>under supervision<br>shared responsibility<br>full autonomy	7.4 (53)<br>12.1 (87)<br>**53.3** (384)<br>27.3 (197)	1.9 (6)<br>13.4 (43)<br>41.3 (133)<br>**43.5** (140)	0.6 (2)<br>11.2 (36)<br>**44.5** (143)<br>43.6 (140)	0 (0)<br>9.7 (31)<br>44.7 (142)<br>**45.6** (145)	0.3 (1)<br>8.9 (28)<br>44.3 (139)<br>**46.5** (146)
evidence based practice	not allowed<br>under supervision<br>shared responsibility<br>full autonomy	8.4 (58)<br>11.6 (80)<br>**47.2** (326)<br>32.9 (227)	3.0 (9)<br>16.8 (51)<br>35.0 (106)<br>**45.2** (137)	1.6 (5)<br>13.4 (41)<br>39.9 (122)<br>**45.1** (138)	0.7 (2)<br>11.8 (36)<br>38.2 (117)<br>**49.3** (151)	1.0 (3)<br>11.0 (33)<br>37.7 (113)<br>**50.3** (151)
decision making	not allowed<br>under supervision<br>shared responsibility<br>full autonomy	16.9 (119)<br>16.1 (113)<br>**47.9** (337)<br>19.1 (134)	4.6 (13)<br>20.6 (58)<br>**44.0** (124)<br>30.9 (87)	2.8 (8)<br>18.3 (53)<br>**49.1** (142)<br>29.8 (86)	0.7 (2)<br>17.4 (50)<br>**49.5** (142)<br>32.4 (93)	1.1 (3)<br>15.4 (44)<br>**48.8** (139)<br>34.7 (99)
communication /discussion with patient/family	not allowed<br>under supervision	6.7 (49)<br>12.2 (89)	1.2 (4)<br>13.2 (46)	0.3 (1)<br>11.7 (39)	0 (0)<br>10.4 (34)	0.3 (1)<br>9.6 (31)

	shared responsibility	**50.3** (368)	41.4 (135)	43.5 (145)	**43.6 (142)**	42.6 (138)
	full autonomy	30.8 (225)	**44.2** (144)	**44.4** (148)	**46.0** (150)	**47.5** (154)
Inter / intraprofessional referrals	not allowed	13.6 (96)	4.8 (14)	2.0 (6)	1.0 (3)	0.7 (2)
	under supervision	13.7 (97)	18.2 (53)	17.1 (50)	15.1 (44)	13.6 (39)
	shared responsibility	**51.6** (364)	**42.3** (123)	**46.1** (135)	**46.7** (136)	**47.2** (135)
	full autonomy	21.1 (149)	34.7 (101)	34.8 (102)	37.1 (108)	38.5 (110)
facilitation of medication management	not allowed	18.0 (125)	3.8 (11)	1.0 (3)	0.3 (1)	0.7 (2)
	under supervision	15.4 (107)	19.9 (58)	19.3 (57)	17.2 (50)	16.3 (47)
	shared responsibility	**40.5** (282)	**39.5** (115)	**43.7** (129)	**43.8** (127)	**42.9** (124)
	full autonomy	26.1 (182)	36.8 (107)	35.9 (106)	38.6 (112)	40.1 (116)
selfcare support and therapeutic education	not allowed	6.3 (45)	1.3 (4)	1.2 (4)	0 (0)	0.3 (1)
	under supervision	9.5 (68)	11.6 (37)	9.8 (32)	8.7 (28)	7.5 (24)
	shared responsibility	**47.7** (343)	40.0 (128)	42.2 (137)	42.5 (137)	41.2 (131)
	full autonomy	36.6 (263)	**47.2** (151)	**46.8** (152)	**48.8** (157)	**50.9** (162)
advice (patient or other healthcare professional)	not allowed	8.5 (59)	3.4 (10)	0.7 (2)	0.3 (1)	0.7 (2)
	under supervision	11.3 (78)	14.8 (44)	13.9 (42)	12.5 (38)	10.1 (30)
	shared responsibility	**53.8** (372)	**42.8** (127)	**46.5** (141)	**44.2** (134)	**44.9** (133)
	full autonomy	26.4 (183)	39.1 (116)	38.9 (118)	42.9 (130)	44.3 (131)
discharge planning, transition of care planning	not allowed	13.0 (92)	5.9 (17)	2.7 (8)	1.0 (3)	1.0 (3)
	under supervision	14.9 (106)	20.1 (58)	17.7 (53)	15.8 (46)	13.8 (40)
	shared responsibility	**50.4** (358)	**41.7** (120)	**47.8** (143)	**49.0** (143)	**48.8** (41)
	full autonomy	21.7 (154)	32.3 (93)	31.8 (95)	34.2 (100)	36.3 (105)
transitional care communication, inter / intraprofessional collaboration / communication including reporting, advising, informing, alerting and discussing	not allowed	8.9 (63)	3.9 (12)	1.0 (3)	0 (0)	0.3 (1)
	under supervision	11.4 (81)	14.8 (46)	11.8 (37)	11.6 (36)	9.4 (29)
	shared responsibility	**55.3** (393)	**45.2** (140)	**49.2** (154)	**49.7** (154)	**50.0** (154)
	full autonomy	24.5 (174)	36.1 (112)	38.0 (119)	38.7 (120)	40.3 (124)
mentoring colleagues	not allowed	8.3 (57)	4.7 (14)	1.3 (4)	0 (0)	0.3 (1)
	under supervision	10.3 (71)	15.4 (46)	14.1 (42)	12.6 (37)	11.3 (33)
	shared responsibility	**49.8** (343)	**40.3** (120)	**43.3** (129)	41.8 (123)	41.0 (120)
	full autonomy	31.6 (218)	39.6 (118)	41.3 (123)	**45.6** (134)	**47.4** (139)

EQF = European qualification framework

[a]n(%) in this column represents opinions of both respondents who were not able to distinguish between levels of education and respondents, who were able to distinguish between levels of education but indicated that for the given task the same degree of responsibility was applicable for all levels of nurse education.

Bold numbers represent the most prevelant level of responsibility (mode) per task, per level of nurse education.
